# Submicrometre sampling of living cells by macrophages

**DOI:** 10.1038/s41586-026-10435-5

**Published:** 2026-04-29

**Authors:** Amy C. Fan, Rukman R. Thota, Nina Serwas, Vivasvan S. Vykunta, Kyle Marchuk, Megan K. Ruhland, Lauren Liu, Grace Johnson, Austin Edwards, Matthew F. Krummel

**Affiliations:** 1 Department of Pathology, University of California, San Francisco, San Francisco, CA USA; 2 Biomedical Sciences Graduate Program, University of California, San Francisco, San Francisco, CA USA; 3 Medical Scientist Training Program, University of California, San Francisco, San Francisco, CA USA; 4 Parnassus Advanced Light Microscopy CoLab, University of California, San Francisco, San Francisco, CA USA; 5 Present Address: Arcus Biosciences, Hayward, CA USA; 6 Present Address: Department of Cell, Developmental and Cancer Biology, Oregon Health & Science University, Portland, OR USA

**Keywords:** Antigen-presenting cells, Organelles, Cellular imaging, Immune tolerance, Imaging the immune system

## Abstract

An effective immune system must sample and develop healthy self-identity to prevent autoimmunity and to discern pathogenic insults^1–3^. Self-proteins are presented to T cells in the thymus during immune cell development^2,3^ and must be presented throughout the body to maintain regulatory T cell populations^4–6^ and to provide tonic signals to sustain conventional T cells over time^7–9^. Observations of continuous apoptosis in some organs together with the ingestion of that material by myeloid populations has led to a conventional understanding of ongoing cell death as a major source of self-antigens^10^. Here we used a series of companion imaging and vesicular labelling technologies to reveal an alternative process undertaken by macrophages that results in non-destructive, direct sampling of living cells. This process requires cell–cell contact, does not require caspase activation and occurs via trogocytosis-like stretching of the target cell into the macrophage, which leads to the generation of submicrometre-sized vesicles that contain cytoplasm. Using a high-dimensional flow-based method for labelling vesicles, we demonstrate that live-sampled material is distinctly processed and is poorly subjected to fusion with lysosomes. The material also produces differential effects on the presentation of antigen to CD4 T cells compared with CD8 T cells. Disruption of this trafficking by redirecting antigen to the lysosome significantly reduced the associated macrophage-mediated priming of CD8 T cells. These results demonstrate an important and substantial sampling of living cells by the immune system, with clear consequences for maintaining the border of immunity.

## Abstract (web summary)

Macrophages can sample antigens from living cells through a trogocytosis-like mechanism that routes ingested material away from degradation, a finding that delineates a previously unknown pathway for antigen presentation to CD8 T cells.

## Main

**Fig. 1:**
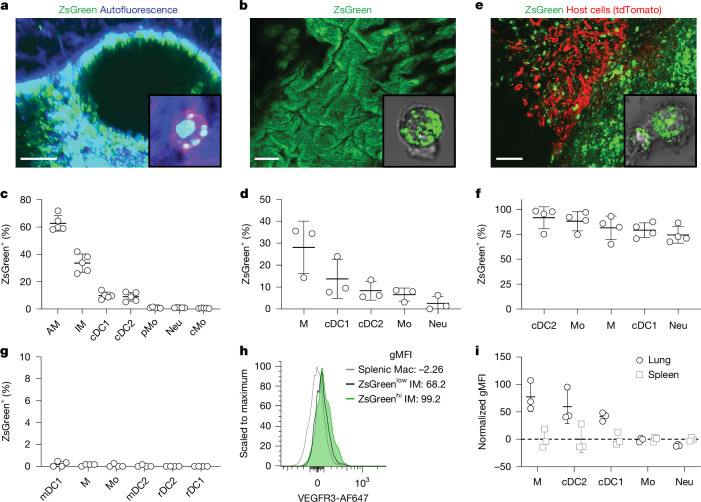
Myeloid cells sample proteins from healthy and tumour tissues. **a**,**b**, Images of example lung (**a**) and skin (**b**) samples taken from mice expressing ZsGreen under the *Scgb1a1*^*creERT2*^ (**a**) or *K14*^*cre*^ (**b**) promoter. Insets show images of in situ macrophages labelled with CD45 (**a**) or sorted CD45^+^ macrophages (**b**) showing ZsGreen puncta inside the macrophages. **c**,**d**, Quantification of isolated lung myeloid cells from *Scgb1a1*^*creERT2*^;*ZsGreen* mice (**c**, *n* = 5 mice) or skin myeloid cells from *K14*^*cre*^;*ZsGreen* mice (**d**, *n* = 3 mice) shows specific uptake in tissues, prominently in macrophages (M). Lymphocytes were routinely negative for ZsGreen. AM, alveolar macrophage; cDC1, type 1 conventional dendritic cell; cDC2, type 2 conventional dendritic cell; cMo, conventional monocyte; IM, interstitial macrophage; Mo, monocyte; Neu, neutrophil; pMo, patrolling monocyte. **e**, Example image of B16 melanoma cells expressing ZsGreen transplanted into mice ubiquitously expressing tdTomato. The inset is an example image of CD45^+^ macrophages. **f**, Quantification of isolated tumour-associated myeloid cells shows broad uptake of ZsGreen across myeloid cell subsets. *n* = 4 mice. **g**, Quantification of isolated inguinal LN myeloid cells from *Scgb1a1*^*creERT2*^;*ZsGreen* mice shows no uptake in distant LNs. *n* = 4 mice. mDC1, migratory cDC1; mDC2, migratory cDC2; rDC1, residential cDC1; rDC2, residential cDC2. **h**, Intracellular staining for airway-specific VEGFR3 protein in lung (black) compared with splenic (grey) myeloid cell populations with ZsGreen^hi^ lung macrophages (green) further enriching for the VEGFR3 geometric mean fluorescence intensity (gMFI) signal. **i**, VEGFR3 gMFI in lung and splenic myeloid cell populations normalized to the average gMFI of splenic myeloid cells. *n* = 3 mice. Representative of 3 experiments, *n* = 3–5 mice per experiment. Shown are mean ± s.d. Scale bars, 40 µm (**a**), 50 µm (**b**) or 100 µm (**e**). Source data

**Extended Data Fig. 1:**
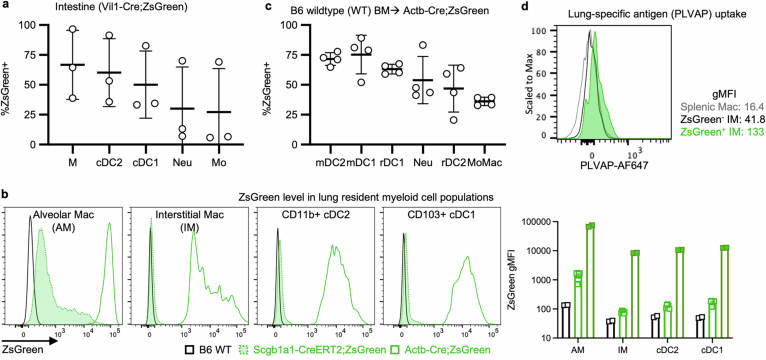
ZsGreen uptake from tissues is not due to Cre mis-expression. **a**, Gut taken from mice expressing ZsGreen under the Villin promoter. Isolation of myeloid cells from Vil1-Cre;ZsGreen mice shows uptake in gut in macrophages as well as DCs. n = 3 mice. **b**, Representative flow (left) or quantification of ZsGreen (right) in lung myeloid cell populations in B6 wildtype mice, Scgb1a1-CreERT2;ZsGreen mice with lung-specific ZsGreen expression, and Actb-Cre-ZsGreen mice. n = 2 B6 WT, 6 Scgb1a1-CreERT2;Ai6, 2 Actb-Cre;Ai6. **c**, Bone marrow transplant of B6 wildtype mice into Actb-Cre;ZsGreen expressing mice. Myeloid cells isolated from skin-draining inguinal lymph nodes show ZsGreen sampling. n = 4 mice. **d**, Intracellular staining for airway specific PLVAP protein in lung (black) compared to splenic (gray) myeloid cell populations with ZsGreen+ lung macrophages (green) enriching for PLVAP signal. Shown are mean +/− standard deviation. Representative of 3 experiments, n = 3-6 mice per experiment. M, macrophage; Neu, neutrophil; Mo, monocyte; mDC2, migratory cDC2; mDC1, migratory cDC1; rDC1, residential cDC1; rDC2, residential cDC2; MoMac, Monocyte/Macrophage. Source data

Antigen-presenting cells (APCs) must continuously survey tissues through the ingestion and processing of antigens for presentation to mediate antigen-specific T cell responses^11^. Previously, in the course of studying a selection of tissue-specific sites of tolerance, including in tumours, we expressed the fluorescent protein ZsGreen in multiple non-inflammatory settings across a range of tissues with slow turnover rates (for example, *Scbg1a1*^*creERT2*^ airway epithelium)^12^ or faster turnover (for example, *K14*^*cre*^ skin cells, tumours and *Vil1*^*cre*^ intestinal epithelium). ZsGreen is both bright and stable, which facilitates long-lived tracking^13,14^. In this study, cytosolic ZsGreen expressed under the *Scbg1a1*^*creERT2*^ and *K14*^*cre*^ promoters all routinely illuminated substantial CD45^+^ immune populations that contained numerous submicrometre-sized vesicular puncta of ZsGreen (Fig. 1a,b), a finding consistent with ingestion of tissue-associated protein. When CD45^+^ cells containing ZsGreen were isolated from these healthy tissues, various myeloid cells were highlighted (Fig. 1c,d, Extended Data Fig. 1a,b and Supplementary Fig. 2), including dendritic cells and neutrophils. However, macrophages were the most consistently loaded. These loading frequencies also mirrored previous characterizations of myeloid sampling of skin^13^ and tumour cytoplasts^14^.

These observations do not result from misexpression of transgenes in host tissues, as demonstrated by the following series of observations. First, we compared the ZsGreen fluorescence intensity of lung myeloid cells in mice expressing ubiquitous ZsGreen (*Actb*^*cre*^;*ZsGreen*), lung-specific ZsGreen (*Scgb1a1*^*creERT2*^;*ZsGreen*) or no ZsGreen (C57BL/6 wild-type (B6 WT)). In each myeloid cell population, ZsGreen fluorescence intensity in *Scgb1a1*^*creERT2*^;*ZsGreen* mice was greater than in B6 WT mice but less than in *Actb*^*cre*^;*ZsGreen* mice (Extended Data Fig. 1b). This result is consistent with the accumulation of exogenous ZsGreen protein as opposed to cell-autonomous ZsGreen expression. Second, transplantation of normal bone marrow into mice expressing ZsGreen gave rise to donor-derived myeloid cell populations that were bright for ZsGreen (Extended Data Fig. 1c and Supplementary Fig. 3a). Third, in mice ubiquitously expressing tdTomato and subcutaneously transplanted with B16-F10 tumours expressing ZsGreen (B16-ZsGreen), host CD45^+^ immune cells contained tumour-derived vesicular ZsGreen puncta. Moreover, a significant proportion of tumour-associated myeloid cells ingested ZsGreen (Fig. 1e,f and Supplementary Fig. 3b). Finally, whereas we had previously observed trafficking of tissue-specific ZsGreen to tissue-draining lymph nodes (LNs)^13^, ZsGreen was not detected in the non-draining LNs of *Scgb1a1*^*creERT2*^;*ZsGreen* mice (Fig. 1g), which indicated tissue-specific uptake.

For tumour antigens, it was previously found that tumour antigens are co-packaged in macrophage vesicles together with tumour-derived ZsGreen tracer^13^. To determine whether other non-tumour self-antigens in healthy tissue with low turnover, such as epithelium, are similarly taken up in these myeloid cell populations, we examined the levels of VEGFR3 and PLVAP proteins, which are specifically expressed on the cell surface of non-haematopoietic cells in the lung. Intracellular flow cytometry analyses of cellular components from the lungs of *Scgb1a1*^*creERT2*^;*ZsGreen* mice showed that local myeloid cell populations from the lung, but not distant splenic myeloid cells, contained these self-proteins (Fig. 1h,i, Extended Data Fig. 1d and Supplementary Fig. 3c). Furthermore, ZsGreen^hi^ myeloid cells were brighter for VEGFR3 and PLVAP stains compared with ZsGreen^low^ myeloid cells (Fig. 1h and Extended Data Fig. 1d). Therefore, tissue-associated myeloid cells regularly sample tissue-associated self-proteins, both engineered tracer proteins and those normally expressed by tissues.

### Macrophages can sample from live cells

**Fig. 2:**
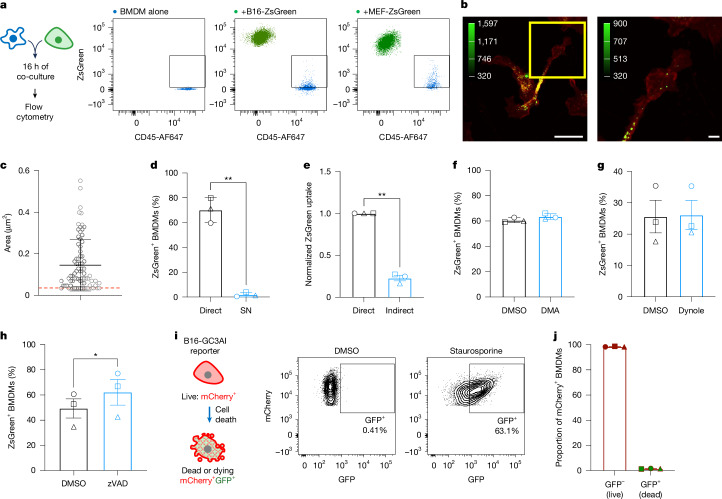
Live cells can be sampled in a cell-contact-dependent manner without caspase activation. **a**, BMDMs and ZsGreen-target cells were co-cultured for 16 h (left) before evaluation for ZsGreen uptake by flow cytometry (right). **b**, Images of sorted ZsGreen^+^ BMDMs with membrane tdTomato showcasing ZsGreen^+^ puncta (representative of *n* = 2 experiments). Right image is the magnification of the yellow square on the left. Colour bar: grey units. Scale bars, 20 µm (left) or 5 µm (right). **c**, Area of individual ZsGreen^+^ vesicles (mean = 0.145 μm^2^). *n *= 77 vesicles quantified from 23 cells. Shown are mean ± s.d. **d**,**e**, BMDMs were co-cultured directly with B16 cells (Direct), with supernatant from B16 cell culture (**d**, SN; *P* = 0.0052) or with a Transwell insert containing B16 cells (**e**, Indirect); *P* = 0.0018). **f**–**h**, In vitro co-cultures were treated with DMSO (vehicle control) or with an exosome and microparticle inhibitor (DMA; **f**), an endocytosis inhibitor (Dynole; **g**) or a caspase inhibitor (zVAD; **h**; *P* = 0.0378). **i**, Schematic (left) and plots (right) of induction of GFP expression in B16-GC3AI cells treated with DMSO or staurosporine. **j**, Co-culture of BMDMs with B16-GC3AI cells demonstrates that uptake is predominantly from live mCherry^+^GFP^−^ target cells. For **d**, **h** and **j**, *n* = 3 biological replicates. Each point represents one biological replicate (mean of *n* = 3 technical replicates). Shown are the mean of biological replicates ± s.e.m. Two-sided paired *t*-test. *P < 0.05, **P < 0.01. Source data

**Extended Data Fig. 2:**
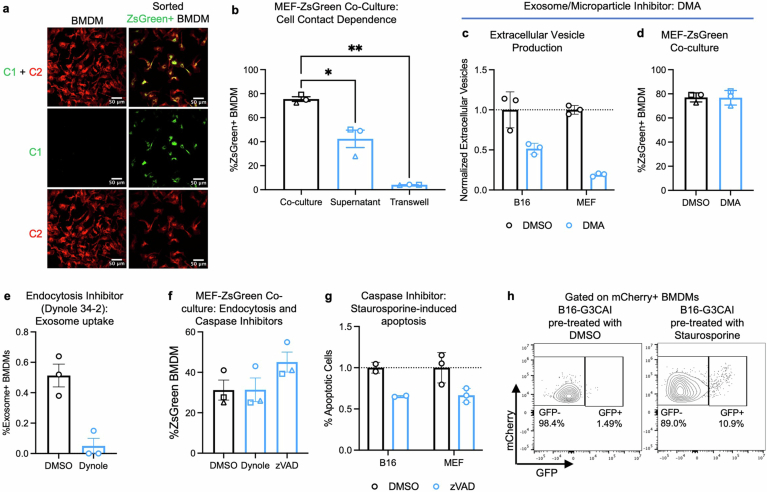
Live cells can be sampled in a cell-contact dependent manner without caspase activation. **a**, Visualization of ZsGreen (C1) puncta ingested from target cells within membrane Tomato (C2) labeled BMDMs. ZsGreen^+^ BMDMs were sorted and imaged after co-culture with B16-ZsGreen cells (representative of 2 experiments). **b**, BMDMs were co-cultured directly with MEFs, with MEF supernatant, or with a transwell insert containing MEFs. p = 0.0451 for Co-culture vs. Supernatant; p = 0.0011 for Co-culture vs. Transwell. One-way ANOVA. *p < 0.05, ** p < 0.01. **c**,**d**, Target cells (**c**) or BMDM-MEF co-cultures (**d**) were treated with DMA or DMSO vehicle control. Extracellular vesicle production in supernatant (**c**) or ZsGreen uptake (**d**) was measured by flow cytometry. **e**, Effect of Dynole on endocytosis of ZsGreen^+^ exosomes by BMDMs. **f**, BMDM-MEF co-cultures were treated with indicated inhibitors or DMSO vehicle control. **g**, Target cells were treated with staurosporine and/or zVAD and AnnexinV^+^DAPI^+/−^ apoptotic cells were measured with flow cytometry. **h**, BMDM co-culture with B16-F10 expressing GFP caspase 3 activity indicator (B16-GC3AI) demonstrates uptake is predominantly from live cells (representative of 2 experiments). n = 3 biological replicates. Each point represents one biological replicate (mean of n = 3 technical replicates). Shown are mean of biological replicates +/− s.e.m. Source data

Several mechanisms of uptake might contribute to myeloid cell sampling from tissues in vivo. The most heavily studied so far is phagocytosis of dead cells and endocytosis of exosomes. To examine in detail how myeloid cells can obtain intracellular material from nearby cells, we established an in vitro assay to measure cell sampling using donor cell populations, grown at around 99% viability, in log phase. We co-cultured bone-marrow-derived macrophages (BMDMs) with cells expressing ZsGreen, focusing on two model target cells: B16-ZsGreen melanoma cells (which, in these conditions, minimally produce exosomes) and primary mouse embryonic fibroblasts (MEFs) isolated from mice ubiquitously expressing ZsGreen (MEF-ZsGreen). From these co-culture experiments, we detected significant ZsGreen^+^ uptake from both target cells into BMDMs. Moreover, the intensity of ZsGreen fluorescence was hundreds of times lower than the intensities of intact donor cells, a result consistent with partial sampling as opposed to complete engulfment (Fig. 2a and Supplementary Fig. 4a). When ZsGreen^+^ BMDMs were sorted for imaging by high-resolution spinning disc confocal microscopy, internalized submicrometre-sized ZsGreen^+^ puncta were readily visualized (Fig. 2b,c and Extended Data Fig. 2a).

We then evaluated whether antigen sampling from live cells in this setting is mediated through the uptake of soluble particles and/or depends on cell contact, whereby the former in particular is expected for the ingestion of free exosomes and perhaps apoptotic blebs. Culture of BMDMs with either B16-ZsGreen-derived supernatant containing a limited number of exosomes produced in a 48-h period or B16-ZsGreen cells that were separated from BMDMs by a Transwell insert both significantly reduced uptake (Fig. 2d,e and Extended Data Fig. 2b). Moreover, treatment of cultures with an inhibitor of exosome and microparticle release or with an inhibitor of endocytosis did not reduce uptake (Fig. 2f,g, Extended Data Fig. 2c–f and Supplementary Fig. 4b,c). Therefore, although endocytosis of soluble material such as exosomes and microparticles may modestly contribute to this feature, this result suggests that there is a distinct dominant mechanism of uptake of live cell-associated material in this setting, which requires cell contact.

An existing hypothesis suggests that phagocytosis of apoptotic bodies, termed efferocytosis but here we will use ‘phagocytosis’, is the major mechanism by which tissues donate material to surveilling APCs^15–17^. We therefore sought to test whether cell death is necessary for the substantial cell sampling observed in our system. When we treated co-cultures with the caspase inhibitor zVAD, there was no effect on ZsGreen uptake (Fig. 2h, Extended Data Fig. 2f,g and Supplementary Fig. 4d). This result confirms that this uptake mechanism does not rely on cell death. To study more directly whether the material ingested into macrophages comes from cells undergoing apoptosis, we used a B16-F10 model cell line that expresses constitutive mCherry and a split GFP caspase-3 activity indicator that fluoresces only when cleaved, for example, during apoptosis^18^ (B16-GC3AI) (Fig. 2i). Use of this model confirmed that B16-GC3AI cells in our assays were highly viable (0.12% GFP^+^), whereas apoptotic cell death induced by staurosporine treatment resulted in GFP reporter fluorescence (>63% GFP, Fig. 2i). When these reporter-expressing cells were co-cultured with BMDMs, the majority of the mCherry^+^ cells (those that had taken up material from the donor cells) lacked GFP expression compared with uptake following apoptosis (1% for live sampling versus 11% after staurosporine treatment; Fig. 2j and Extended Data Fig. 2h). Together, these results provide further support that an alternative sampling pathway, beyond those involving apoptosis, exists to obtain material from live cells.

### Live imaging of live sampling

Given the small size of particles we found in macrophages, we adopted high-resolution methods to directly image the time course of B16-ZsGreen cells interacting with BMDMs expressing membrane tdTomato in co-culture. We used lattice light-sheet imaging (which has an isotropic resolution of approximately 220 nm) and a Nikon spatial array confocal (NSPARC) detector system (which uses an ultralow noise detector array with lateral and axial resolutions of approximately 212 nm and 424 nm, respectively). Note that both technologies can sample live full-cell volumes over time with reduced phototoxicity.

**Fig. 3:**
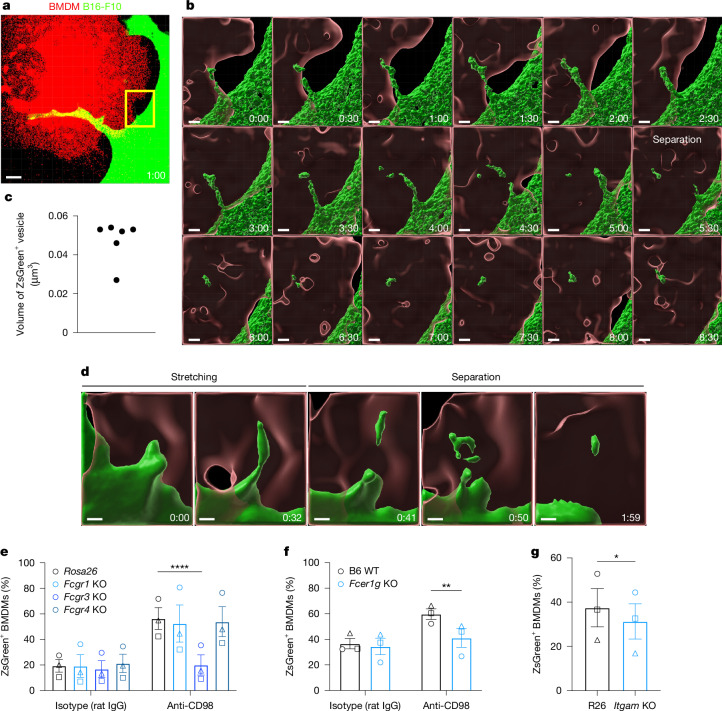
Trogocytosis-like sampling enables macrophages to ingest cytosolic protein from live cells. **a**,**b**, Confocal live imaging of interactions between tdTomato^+^ BMDMs and B16-ZsGreen cells using the NSPARC detector system. Time shown as min:s (representative of three experiments). **a**, Two-colour volume rendering displaying interactions between tdTomato^+^ BMDMs and B16-ZsGreen cells. The yellow box highlights the region of interest displayed in **b**. **b**, Three-dimensional surface rendering from the region of interest in **a** visualizing the formation and separation of a vesicle from a B16-ZsGreen cell (green) into a tdTomato^+^ BMDM (red). *z*-stacks within series were captured in 30 s intervals. **c**, Volume measurements of separated ZsGreen^+^ vesicles from frames 13–18 in **b** using Imaris object statistics. **d**, Lattice light-sheet imaging of tdTomato^+^ BMDMs and B16-ZsGreen cells capturing the stretching and separation of a ZsGreen^+^ vesicle from the target cell. Time shown as min:s (representative of two experiments). **e**,**f**, Antibody-opsonized B16-ZsGreen target cells were co-cultured with BMDMs edited using CRISPR–Cas9 and single-guide RNA targeting the *Rosa26* control locus or the indicated receptor locus (**e**) or with BMDMs isolated from B6 WT mice (WT) or *Fcer1g* KO mice (**f**). *P* = 0.000003973 in *Fcgr3* KO versus *Rosa26* for anti-CD98 in **e**; *P* = 0.0056 in WT versus *Fcer1g* KO for anti-CD98 in **f**. **g**, BMDMs edited for the *Rosa26* control locus (R26) or the *Itgam* locus (which encodes CD11b) were co-cultured with B16-ZsGreen target cells. *P* = 0.0412. *n* = 3 biological replicates. Each point represents one biological replicate (mean of *n* = 3 technical replicates). Shown are the mean of biological replicates ± s.e.m. *P* values were calculated using two-way analysis of variance (ANOVA) with Šidák’s multiple comparison (**e**), mixed-effects model with Dunnett’s multiple comparison (**f**) or two-sided paired *t*-test (**g**). *P < 0.05, **P < 0.01, ****P < 0.0001. Scale bars, 3 µm (**a**), 0.7 µm (**b**) or 0.5 µm (**d**). Source data

**Extended Data Fig. 3:**
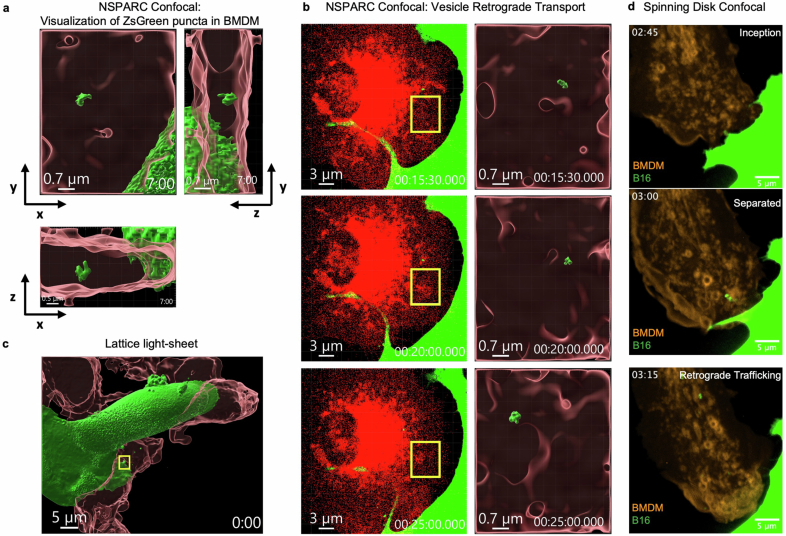
A trogocytosis-like process can sample target cell protein. **a**, Three-dimensional surface renderings from the YZ (top right) and XZ (bottom left) directions to identify intracellular localization of separated vesicle from same ROI as Fig. 3a,b. **b**, Two-color volume rendering showing interactions between TdTomato+ BMDM and ZsGreen+ B16(left) captured immediately after the series in Fig. 3b. Three-dimensional surface renderings(right) of ROIs show separated vesicle trafficking towards the nucleus. Time shown as hh:mm:ss. **a**,**b**, representative of 3 experiments **c**, Three-dimensional surface rendering from Lattice light-sheet imaging of TdTomato^+^ BMDM and ZsGreen^+^ B16 (representative of 2 experiments) **d**, Spinning-disk confocal imaging of ZsGreen+ B16 and TdTomato+ BMDM showing ingested ZsGreen+ puncta within BMDMs. Time shown as hh:mm.(representative of 6 experiments).

Using NSPARC microscopy, we observed target cell–BMDM interactions that spontaneously resulted in the stretching of a protrusion of the target cell into BMDMs. This was frequently followed by the separation of a distinct ZsGreen^+^ vesicle (Fig. 3b). In a representative video (Fig. 3b and Supplementary Video 1), this entire process took <10 min from imaging of the initial ZsGreen^+^ protrusion until the time when the protrusion was no longer visible from the target cell. However, we registered the actual abscission of the vesicle within a single frame (30 s; Fig. 3a,b) followed by complete loss of the remaining tether either into the vesicle or back into the donor cell. We confirmed at the end of the process that minute amounts of target cell material were physically inside the BMDM by analysing the same data in *yz* and *xz* projections (Extended Data Fig. 3a). Consistent with the size ranges observed in vivo^13^, these separated vesicles were small, ranging from diffraction-limited sizes (about <0.02 μm^3^) to about 0.05 μm^3^ (Fig. 3c). In some cases, we also saw a protrusion pulled into the BMDM but then retracted without detectable pinching of a vesicle, which might either have led to extremely tiny ingestion or was simply abortive. After ingestion, ZsGreen^+^ puncta moved within the cytoplasm (Extended Data Fig. 3b). These data suggest that live-cell cytosolic material can be sampled in a contact-dependent, trogocytosis-like manner.

To confirm that this process is not an artefact of the detection method and to take advantage of faster frame rates, we leveraged lattice light-sheet microscopy^19^ because of its isotropic high resolution. This system also captured the stretching and separation of ZsGreen^+^ vesicles, again visible across a single frame (in lattice light-sheet microscopy about 8 s; Fig. 3d, Extended Data Fig. 3c and Supplementary Video 2). Although ingested vesicles were occasionally identified using spinning disc confocal microscopy (Extended Data Fig. 3d), it is possible that the small size of these vesicles (which limits the total fluorescent yield) and the lower sampling rate of this ingestion method hinders our ability to capture this biology with older technologies. This limitation provides a possible explanation for why this process had not yet been described or extensively studied when using conventional microscopy methods.

### Receptor-mediated uptake from live cells

**Extended Data Fig. 4:**
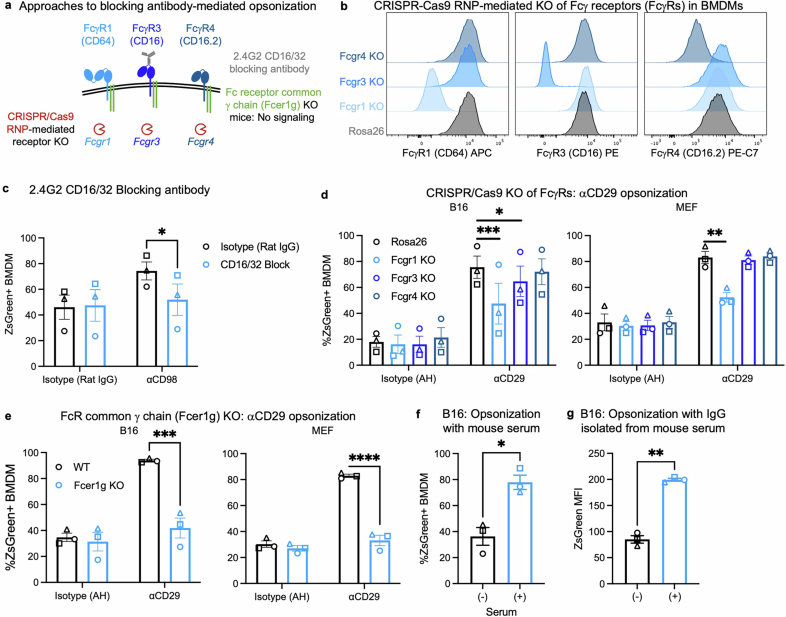
Antibody opsonization amplifies live-cell sampling. **a**, Schematic outlining methods used to block or knock-out (KO) FcγR binding and signaling. **b**, BMDMs targeted at the Rosa26 control locus or receptor loci using CRISPR-Cas9 RNP (representative of 5 experiments). **c**, BMDMs were pre-incubated with 10 ug/mL 2.4G2 CD16/32 blocking antibody or Rat IgG2b prior to co-culture with antibody-opsonized B16-ZsGreen target cells Two-way ANOVA with Sidak’s multiple comparisons test. p = 0.0136. **d**,**e**, CRISPR-targeted BMDMs (**d**) or BMDMs isolated from B6 wildtype or FcγR KO (**e**) mice were co-cultured with B16-ZsGreen or MEF-ZsGreen target cells pre-coated with antibody. p = 0.0002 (R26 vs. Fcgr1 KO αCD29 B16); p = 0.0281 (R26 vs Fcgr3 KO αCD29 B16); p = 0.0093 (R26 vs. Fcgr1 KO αCD29 MEF) in **d**. p = 0.0003 (WT vs Fcer1g KO αCD29 B16); p = 0.0000006538 (WT vs Fcer1g KO αCD29 B16) in **e**. **f**,**g**, BMDMs co-cultured with B16-ZsGreen pre-coated with serum (**f**, p = 0.0375), or IgG isolated from mouse serum (**g**, p = 0.0032). n = 3 biological replicates. Each point represents one biological replicate (mean of n = 3 technical replicates). Shown are mean of biological replicates +/− s.e.m. Two-tailed paired t-test. *p < 0.05, **p < 0.01. Source data

**Extended Data Fig. 5:**
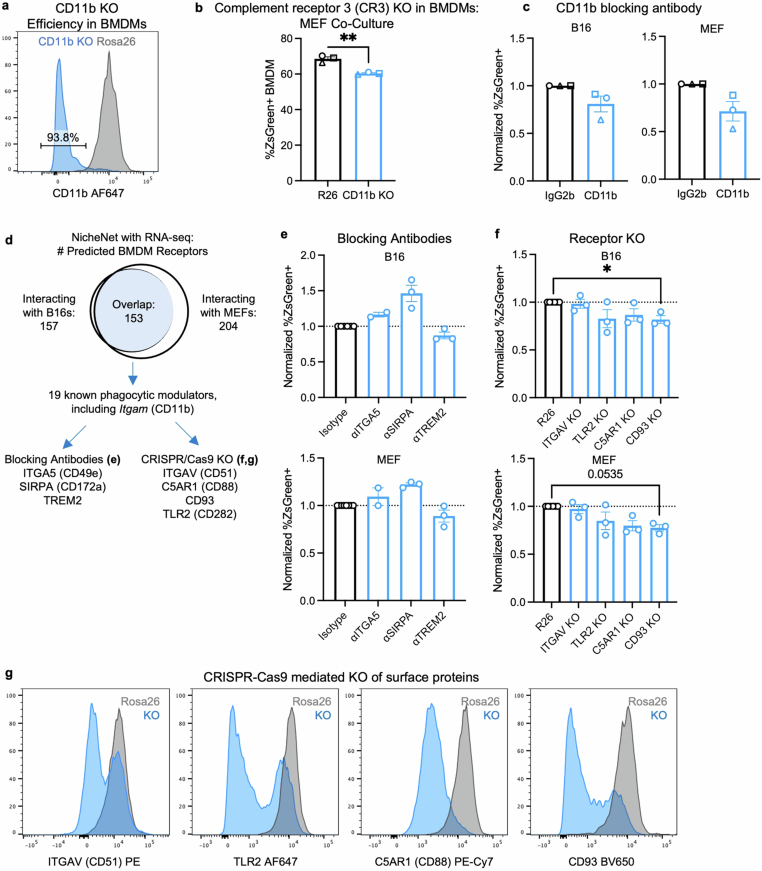
Knockout of CD11b and CD93 decreases live sampling. **a**, Representative protein knockdown from 2 experiments 4 days after CRISPR/Cas9-mediated gene targeting. **b**, ZsGreen uptake in KO cells compared to Rosa26 (R26) controls. p = 0.0092. **c**, BMDMs were pre-treated with 1ug/uL IgG2b or CD11b-blocking antibody for 1 h before 16-hour co-culture antigen transfer assays. **d**, Schematic outlining strategy to identify potential modulators of live sampling. **e**, BMDMs were co-cultured with B16-ZsGreen or MEF-ZsGreen target cells for 16 h with 5 ug/mL blocking antibody or the appropriate isotype control prior to flow cytometric analysis of ZsGreen uptake. n = 2 biological replicates for αITGA5 and 3 biological replicates for αSIRPA and αTREM2. Data normalized to Isotype and compiled over 2 experiments for B16 co-cultures and 3 experiments for MEF co-cultures **f**,**g** BMDMs targeted at the *Rosa26* control locus or receptor loci using CRISPR-Cas9 RNP and co-cultured with B16-ZsGreen or MEF-ZsGreen target cells for 16 h prior to flow cytometric analysis of ZsGreen uptake (**f**, p = 0.0415 for B16 CD93 KO) and receptor expression (**g**). Data normalized to R26 controls and compiled over 2 experiments. n = 3 biological replicates for **b**, **c**, **f**. Shown are mean +/− s.e.m. Kruskal-Wallis test with Dunn’s multiple comparisons test. *p < 0.05, **p < 0.01. Source data

Although multiple receptor–ligand interactions are likely to contribute to this process, we first asked whether previously described mechanisms, such as antibody opsonization or complement receptor 3 (CR3; composed of the CD11b–CD18 heterodimer) engagement, facilitate sampling from live cells. To this end, pre-incubation with antibodies against the surface protein CD98 increased ZsGreen uptake (Fig. 3e). Consistent with previously reported binding specificity of rat IgG1κ to Fcγ receptor 3 (FcγR3, also known as CD16)^20^, this enhancement required FcγR3 binding and signalling (Fig. 3e,f and Extended Data Fig. 4a–c). Pre-incubation with antibodies against another abundant surface protein, CD29, similarly increased uptake in a FcγR-dependent manner (Extended Data Fig. 4d,e). Moreover, pre-incubation of ZsGreen target cells with normal mouse serum or isolated IgG before co-culture with BMDMs increased ZsGreen uptake (Extended Data Fig. 4f,g). This result suggests that even weakly cross-reactive collections of antibodies amplify sampling from live cells. Furthermore, disruption of complement receptor component CD11b in BMDMs decreased the frequency and intensity of ZsGreen uptake in B16 cells and MEFs (Fig. 3g and Extended Data Fig. 5a–c). This result is similar to previous reports of synaptic pruning by microglia involving these receptors^21^.

Next, to identify other surface proteins beyond CD11b and FcγR involved in this process, we used NicheNet^22^ to predict ligand–receptor interactions between our two model target cells and BMDMs (Extended Data Fig. 5d). Because CD11b also has established roles in phagocytosis, we prioritized shared receptors with known phagocytic functions^23^, confirmed their protein expression and evaluated their contribution to ZsGreen uptake. Of the eight candidate receptors that we examined, only knockout (KO) of *Cd93*, which encodes a C-type lectin receptor, showed a modest but significant decrease in ZsGreen uptake (Extended Data Fig. 5e–g). These findings indicate that multiple surface interactions contribute to uptake, and CD11b and CD93 have partial roles.

**Extended Data Fig. 6:**
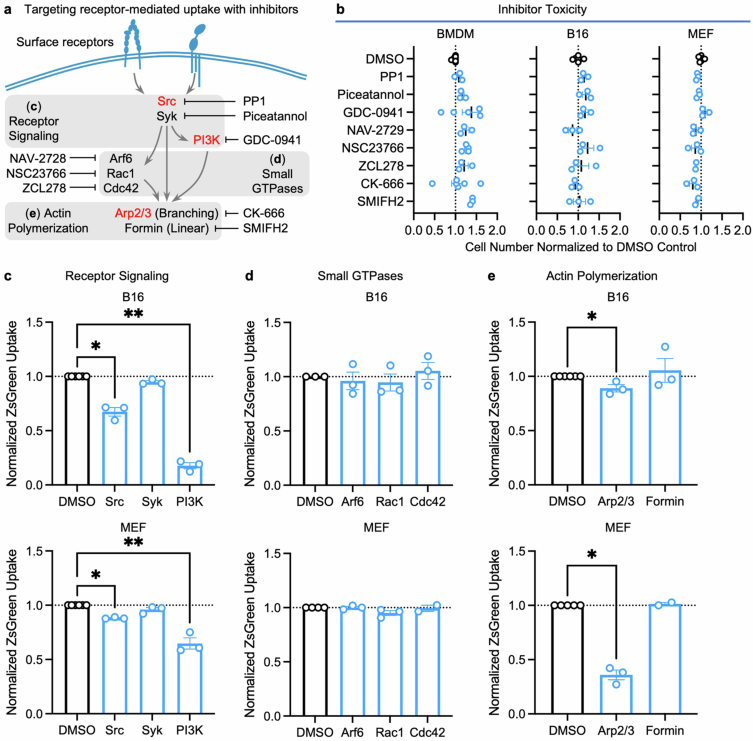
Src, PI3K, and Arp2/3 inhibition decreases live sampling. **a**, Schematic illustrating inhibitors used to target components of receptors-mediated uptake. **b**–**e**, BMDMs, B16-ZsGreen target cells, and MEF-ZsGreen target cells were cultured alone or co-culture for antigen transfer assay and treated with DMSO control, 10 uM PP1, 5 uM Piceatannol, 10 uM GDC-0941, 1.35 uM NAV-2729, 25 uM NSC23766, 10 uM ZCL278, 200 uM CK-666, or 5 uM SMIFH2 for 16 h prior to flow cytometry analysis for cell number (**b**) or ZsGreen uptake (**c**–**e**). (**c**) p = 0.0429 (DMSO vs Src B16); p = 0.0018 (DMSO vs PI3K B16); p = 0.0429 (DMSO vs Src MEF); p = 0.0018 (DMSO vs PI3K MEF). (**e**) p = 0.0197 (DMSO vs Arp2/3 B16); p = 0.0437 (DMSO vs Arp2/3 MEF) Data are normalized to DMSO control and compiled over 5 experiments for BMDM inhibitor toxicity in **b**, 3 experiments for B16 and MEF toxicity in **b**, and 2 experiments for **c**–**e**. n = 3 biological replicates for each inhibitor. Shown are mean +/− s.e.m. Kruskal Wallis test and Dunn’s multiple comparisons. *p < 0.05; **p < 0.01. Source data

We next examined signalling pathways that link receptor engagement to uptake using small-molecule inhibitors. We targeted signalling downstream of CD11b (SRC, SYK and PI3K), small GTPases important for vesicle trafficking (ARF6, CDC42 and RAC) and actin nucleation proteins (ARP2 and ARP3 (ARP2/3) and formins) (Extended Data Fig. 6a,b). Inhibition of SRC signalling (with PP1), PI3K signalling (with GDC-0941) and ARP2/3 (with CK-666) decreased but did not completely abrogate uptake (Extended Data Fig. 6c–e). These results suggest that receptor-mediated activation of SRC and PI3K, which leads to ARP2/3-driven branched actin assembly, supports vesicle formation. By contrast, SYK, small GTPases and formin-driven linear actin may be dispensable or redundant in our system. Altogether, these data indicate that pleiotropic mechanisms contribute to this contact-dependent sampling process that nevertheless relies on local activation cues at the cell–cell interface.

### Alternative trafficking of live sampling

**Fig. 4:**
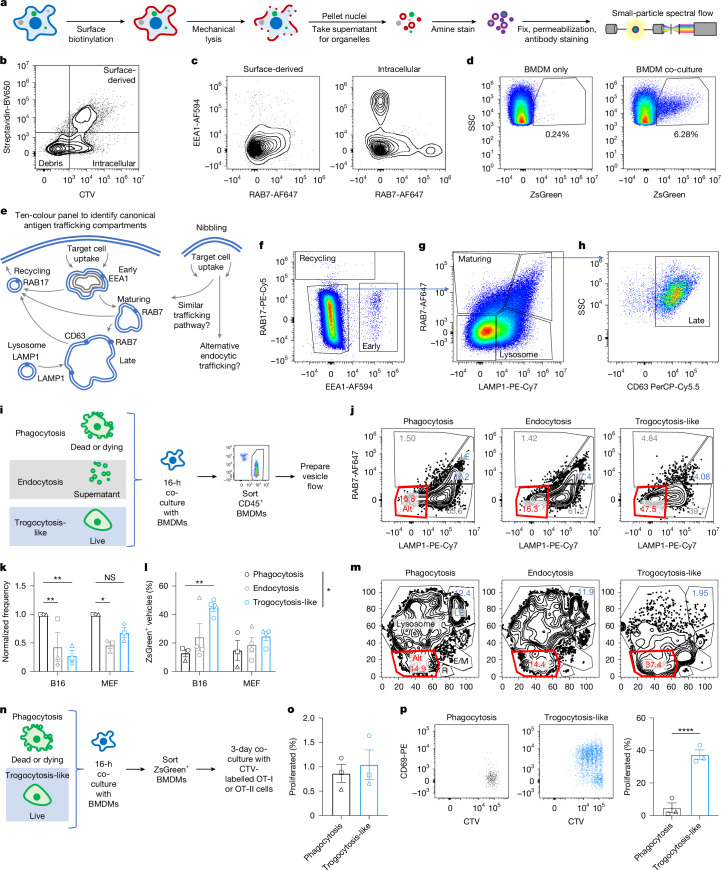
Multiparameter intracellular vesicle flow cytometry reveals that live-cell-associated protein fills a discrete vesicular compartment. **a**, Schematic of multiparameter organelle flow cytometry. Surface-biotinylated cells are lysed and centrifuged at low speed. Supernatant containing the organelles was collected, and the organelles were stained with CellTraceViolet (CTV), fixed, permeabilized and stained with antibodies. **b**, Discrimination of streptavidin-negative CTV^+^ intracellular, protein-containing particles. **c**, Vesicle-staining antibodies primarily stain intracellular particles. **d**, Discrimination of ZsGreen^+^ vesicles containing target-cell-derived protein. SSC, side scatter. **e**–**h**, Identification of vesicle compartments. Schematic (**e**) of how a ten-colour panel was used to identify canonical antigen-trafficking compartments. Gating strategy used to identify recycling and early endosomes (**f**), maturing endoscopes and lysosomes (**g**) and late endosomes (**h**). **i**, Schematic of the evaluation of trafficking downstream of different sampling mechanisms (related to **j**–**m** and Extended Data Figs. 7 and 8). **j**–**m**, Representative flow plots gated on ZsGreen^+^ vesicles (**j**) or tSNE generated from concatenated ZsGreen^+^ vesicles (**m**) derived from phagocytosis, endocytosis or trogocytosis-like mechanisms, with quantification of the proportion of ZsGreen^+^ vesicles in late endosome (**k**) and an alternative compartment (Alt; **l**). *n* = 3 biological replicates for phagocytosis, *n* = 4 biological replicates for endocytosis and trogocytosis-like. For **k**, *P* = 0.0087 for B16 phagocytosis versus endocytosis; *P* = 0.0016 for B16 phagocytosis versus trogocytosis-like; *P* = 0.0121 for MEF phagocytosis versus endocytosis. For **l**, *P* = 0.0053 for B16 phagocytosis versus trogocytosis-like. E/M, early/maturing endosome; LE, late endosome; R, recycling endosome. **n**, Schematic of evaluation of T cell activation downstream of different sampling mechanisms. ZsGreen^+^ BMDMs were sorted after co-culture with live or apoptotic B16-ZsGreen-minOVA target cells and co-cultured with CTV-labelled OT-I CD8^+^ or OT-II CD4^+^ T cells (1:3 ratio of BMDMs to T cells) for 3 days (related to **o** and **p** and Extended Data Fig. 9). **o**, OT-II CD4 T cell proliferation assessed by loss of CTV after co-culture with ZsGreen^+^ BMDMs that ingested material using phagocytosis (black) or trogocytosis-like (blue) mechanisms. **p**, Activation (CD69 expression) and proliferation of OT-I CD8 T cells after co-culture with ZsGreen^+^ BMDMs from phagocytosis or trogocytosis-like conditions. *P* = 0.000002615. *n* = 3 biological replicates for **o** and **p**, Each point represents one biological replicate (*n* = 1 technical replicate for **k** and **l**; *n *= 3 technical replicates for **o** and **p**). Shown are the mean of biological replicates ± s.e.m. *P* values were calculated using two-way ANOVA with Dunnett’s multiple comparisons test (**k** and **l**) or two-sided paired *t*-test (**p**). *P < 0.05, **P < 0.01, ****P < 0.0001; NS, not significant. Source data

We sought to determine whether this mechanism has any distinct functional downstream consequences for the vesicle or cargo by first interrogating its fate compared with material obtained through phagocytosis or endocytosis. To facilitate a high-dimensional analysis of the organelles that were derived from different modes of cell sampling, we developed a ten-parameter flow-based organelle profiling method that is loosely based on a previous phagoFACS method^24^. This new method is capable of immunophenotyping of multiple intracellular compartments (Fig. 4a and Methods). To focus our analysis on protein-associated intracellular organelles, we incorporated a cell-surface biotinylation step before lysis to ensure definitive discrimination of surface-derived membranes and stained preparations with amine-binding CellTraceViolet (Fig. 4b, Methods and Supplementary Fig. 5a). Markers for intracellular organelles, such as early endosome antigen 1 (EEA1) and RAB7, were exclusively detected in the streptavidin-negative material (Fig. 4c). In CellTraceViolet^+^ intracellular vesicles isolated from BMDMs, we were able to distinguish ZsGreen^+^ antigen-containing vesicles from ZsGreen^−^ antigen-free vesicles, and these were exclusively found in BMDMs that had been co-cultured with ZsGreen-expressing target cells (Fig. 4d and Supplementary Fig. 5b).

**Extended Data Fig. 7:**
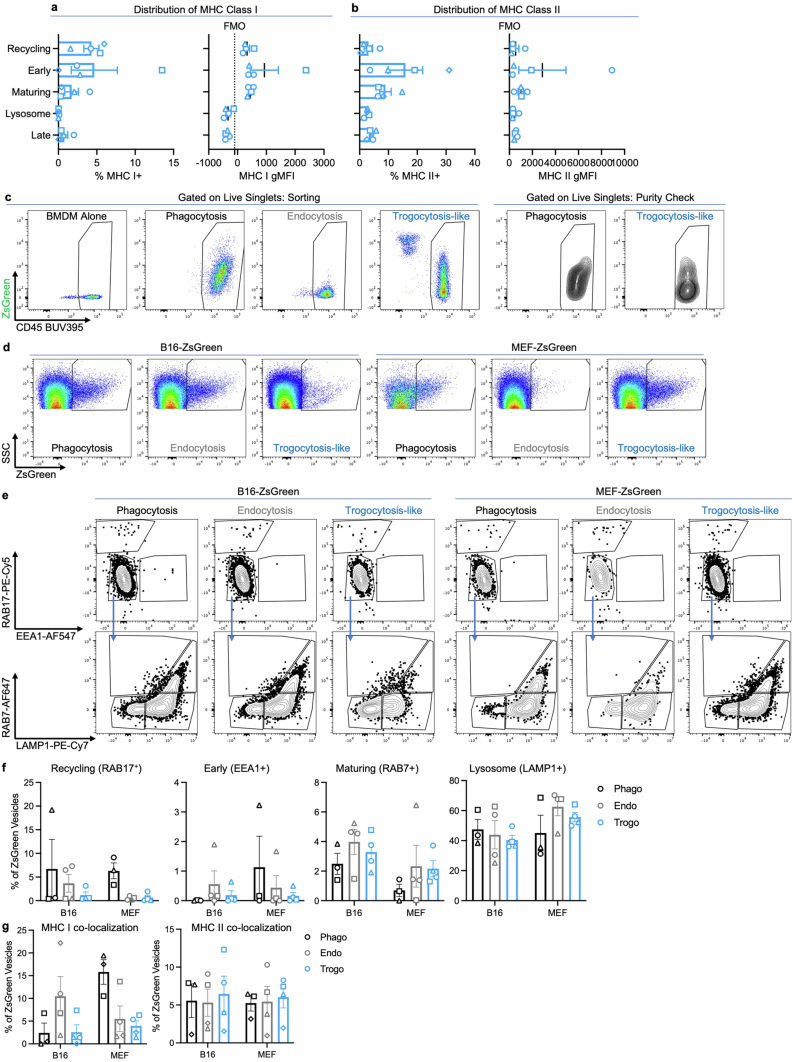
Live-cell associated antigen fills a discrete vesicular compartment. **a**,**b**, Distribution of MHC I (**a**) and MHC II (**b**) in identified vesicular compartments inclusive of both ZsGreen^+^ and ZsGreen^−^ vesicles. **c**, Representative sorting gate and purity check for CD45^+^ BMDMs from 16-hour co-culture. **d**, Representative ZsGreen^+^ gating from different vesicle flow preps gated on CellTraceViolet+Streptavidin- intracellular vesicles. **e**, Representative flow cytometry of vesicle populations from phagocytosis, endocytosis, and trogocytosis of B16-ZsGreen or MEF-ZsGreen target cells. **f**, Summary quantification of proportion of ZsGreen^+^ vesicles in different vesicular compartments. **g**, Proportion of ZsGreen+ vesicles that were either MHC I^+^ or MHC II^+^. **c**–**e** Representative of 3 experiments for Phagocytosis (“Phago”) and 4 experiments for endocytosis (“Endo”) and trogocytosis (“Trogo”). n = 3 biological replicates for phagocytosis (“Phago”), 4 biological replicates for endocytosis (“Endo”) and trogocytosis (“Trogo”). Shown are mean of biological replicates +/− s.e.m. Each point represents one biological replicate (n = 1 technical replicate). Shown are mean of biological replicates +/− s.e.m. Source data

Vesicles derived from internalization are understood to flow through a pathway that consists of progressively more degradative compartments. That is, early endosomes mature into late endosomes that fuse with lysosomes to form phagolysosomes^10,25^. We designed our panel to identify known vesicular compartments, including RAB17^+^ recycling endosomes, EEA1^+^ early endosomes, LAMP1^+^ lysosomes, RAB7^+^ maturing endosomes and RAB7^+^LAMP1^+^CD63^+^ late endosomes (Fig. 4e–h). By further incorporating antibodies that recognize the antigen-presentation molecules major histocompatibility complex (MHC) class I (MHC-I) and class II (MHC-II), we could identify compartments consistent with antigen loading. Consistent with previous reports^10^, MHC-I was primarily excluded from LAMP1^+^ vesicles, whereas MHC-II was detected across multiple vesicular compartments (Extended Data Fig. 7a,b). Together, these results highlight the suitability of this high-dimensional vesicle analytical method to study the composition and identity of vesicular compartments.

We then applied this organelle immunophenotyping method to compare the intracellular trafficking of ZsGreen^+^ antigen-containing vesicles after trogocytosis-like live sampling to other sampling mechanisms, including endocytosis and phagocytosis (Fig. 4i). Although endocytosis of soluble material derived from supernatant resulted in very low levels of ZsGreen uptake, sufficient ZsGreen vesicles were nevertheless detectable for downstream analyses (Extended Data Fig. 7c,d). In a conventional gating analysis, both phagocytosis and endocytosis resulted in distributions of proteins across the vesicle-maturation spectrum. However, it revealed decreased trafficking of vesicles resulting from trogocytosis-like sampling to the late endosome (Fig. 4j,k and Extended Data Fig. 7e,f) and target-cell-dependent changes in the colocalization of these with MHC-I but not MHC-II (Extended Data Fig. 7g). Notably, although around 90% of ZsGreen^+^ vesicles resulting from phagocytosis were associated with a conventional vesicle marker, a substantial proportion of ZsGreen^+^ vesicles resulting from live sampling did not associate with these canonical endocytic markers (Fig. 4l). This result suggests that there is sequestration of live sampled material into an alternative endocytic trafficking pathway rather than those associated with degradation.

**Extended Data Fig. 8:**
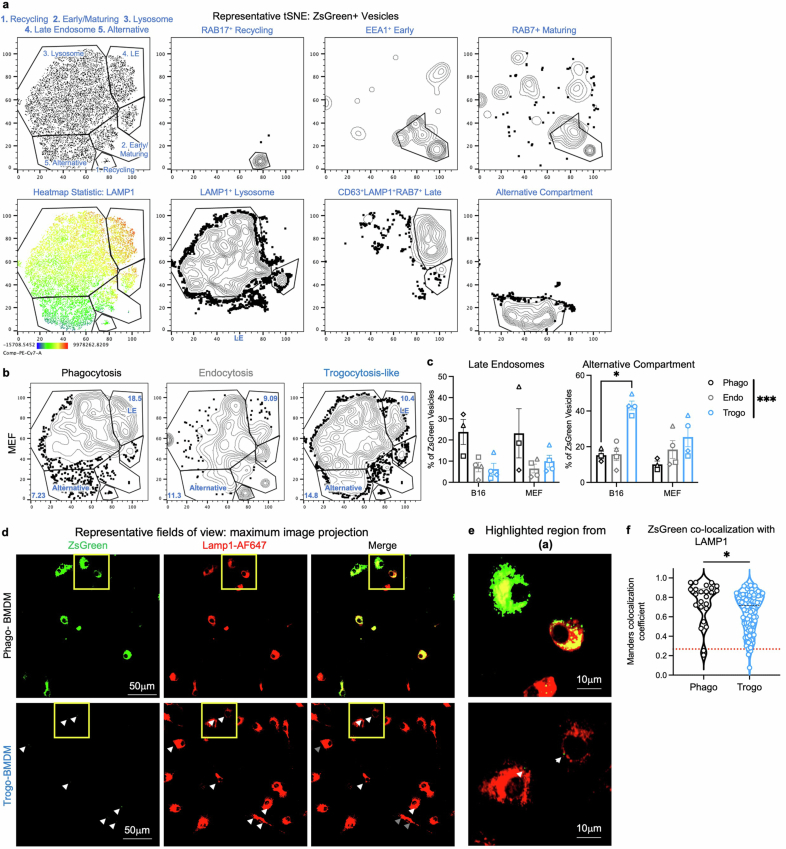
Live-cell associated antigen fills a LAMP1-negative vesicular compartment. **a**, tSNE of concatenated ZsGreen^+^ vesicles from one experiment demonstrating gating of different vesicular compartments. **b**, Distribution of ZsGreen^+^ vesicles in derived from different sampling mechanism. **c**, Summary quantification of proportion of ZsGreen^+^ vesicles in late endosome and alternative vesicular compartment gates by gating on tSNE populations. **a**,**b** Representative of 3 experiments for Phagocytosis (“Phago”) and 4 experiments for endocytosis (“Endo”) and trogocytosis (“Trogo”). n = 3 biological replicates for phagocytosis (“Phago”), 4 biological replicates for endocytosis (“Endo”) and trogocytosis (“Trogo”). (n = 1 technical replicate per biological replicate). Shown are mean of biological replicates +/− s.e.m. Mixed effects model with Dunnett’s multiple comparisons test. p = 0.0002 fixed effect size of sampling method (Phago, Trogo, Endo); p = 0.0146 for Phago vs. Trogo B16 Alternative Compartment. **d**–**f**, Sorted ZsGreen+ BMDMs after co-culture with killed B16-ZsGreen (Phago-BMDM) and live B16-ZsGreen (Trogo-BMDM) were fix, permeabilized, and stained with LAMP1-AF647. Representative images from 2 experiments are shown in (**d**,**e**) and quantification per field of view is shown in (**f**). Phago, n = 24; Trogo, n = 96. Two-sided student’s t-test. p = 0.0370. *p < 0.05, ***p < 0.001. Source data

To analyse vesicle populations on the basis of their intensity for each of our markers and in an unbiased manner to previous classifications, we displayed the concatenated ZsGreen^+^ vesicles in a *t*-distributed stochastic neighbour embedding (tSNE) plot using only the vesicle protein marker intensities as parameters (Extended Data Fig. 8a,b). This approach demonstrated that although vesicles derived from each sampling method overlapped, there was a distinct shift in the distribution of vesicles when comparing live sampling (trogocytosis-like) with conventional phagocytosis of apoptotic cells (Fig. 4m and Extended Data Fig. 8b). When we overlayed intensities of markers such as LAMP1, and consistent with the conventional flow-gating strategy, vesicles resulting from live sampling were relatively depleted in late endosomes and enriched in an alternative vesicular compartment (Fig. 4m and Extended Data Fig. 8c).

To independently assess lysosomal delivery, we performed confocal microscopy and quantitative colocalization analyses. The results confirmed that ZsGreen acquired through live sampling had significantly lower overlap with LAMP1 than with antigen acquired from phagocytosis (Extended Data Fig. 8d–f). This finding provides further support that live-cell-derived antigen has a distinct vesicular fate.

### Live sampling biases T cell activation

**Extended Data Fig. 9:**
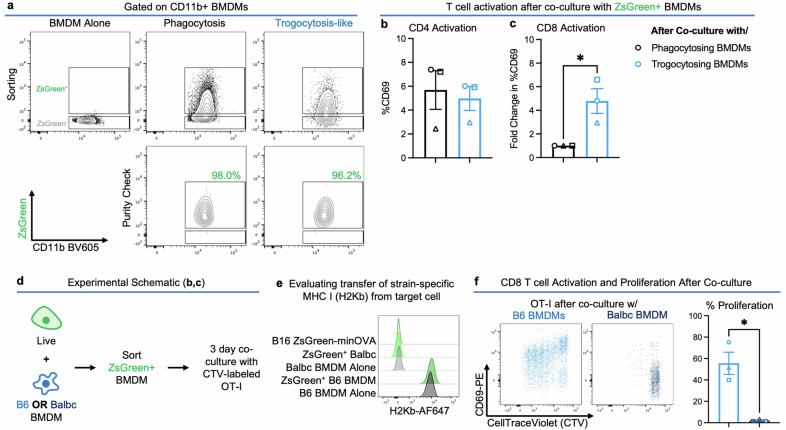
Macrophages process live-cell associated antigen to affect CD8 T cell activation. **a**, Representative flow plots from 3 experiments showing sorting and purity of ZsGreen+ BMDMs after co-culture with live or apoptotic B16F10-ZsGreen-minOVA target cells. **b**,**c**, Activation and proliferation of OT-I CD8 T cells (**b**) and OT-II CD4 T cells (**c**) as read out by CD69 expression after 3 days of co-culture with ZsGreen+ BMDMs from phagocytosis (black) or trogocytosis (blue) conditions. Two-tailed unpaired t-test. p = 0.0226. **d**, Experimental schematic to evaluate strain specific MHC-I transfer and T cell activation by ZsGreen^+^ B6 or Balbc-derived BMDMs. **e**, H2-Kb expression on B16 ZsGreen-minOVA target cells; ZsGreen+ BMDMs after co-culture with B16 ZsGreen-minOVA cels, and BMDMs cultured alone (representative of 3 experiments). **f**, OT-I CD8 T cell proliferation after 3-day co-culture with FACS-isolated ZsGreen+ B6 or Balb/c BMDMs. p = 0.0368. n = 3 biological replicates. Each point represents one biological replicate (mean of n = 3 technical replicates). Shown are mean of biological replicates +/− s.e.m. Two-sided paired t-test. *p < 0.05. Source data

Immunologically, the nature of sampling may have repercussions for how internalized antigens are presented to T cells on MHC-I (cross-presentation) compared with MHC-II. To examine how sampling through this trogocytosis-like mechanism affects antigen presentation, we isolated ZsGreen^+^ BMDMs after co-culture with DMSO-treated or staurosporine-treated B16-F10 cells expressing ZsGreen fused to OT-I and OT-II ovalbumin (OVA) peptides (B16-ZsGreen-minOVA cells)^13^ (Fig. 4n and Extended Data Fig. 9a). We detected only weak and indistinguishable activation in OT-II CD4 T cell activation after co-culture with BMDMs that had acquired antigen through phagocytosis (Phago-BMDMs) compared with those that had sampled live cells (Trogo-BMDMs) (Fig. 4o and Extended Data Fig. 9b). By contrast, Trogo-BMDMs but not Phago-BMDMs induced significant OT-I CD8 T cell proximal activation (CD69 upregulation) and proliferation (Fig. 4p and Extended Data Fig. 9c). This result is consistent with the absence of trafficking of live-sampled material to the degradative late endosome and lysosomal compartments and suggests that there is a bias for cross-presentation.

Trogocytosis that leads to transcellular membrane transfer (cross-dressing) and T cell activation has been described in dendritic cells^26^. To determine whether this process is occurring in our system, we performed antigen transfer and T cell-stimulation assays using BMDMs derived from BALB/c mice (Extended Data Fig. 9d). The MHC-I allele *H2Kb* was undetectable on both BALB/c-derived BMDMs cultured alone and on ZsGreen^+^ BALB/c BMDMs that had ingested material from B16-ZsGreen-minOVA target cells (Extended Data Fig. 9e). Consistent with this finding, ZsGreen^+^ BALB/c-derived BMDMs induced significantly less antigen-specific CD8 T cell proliferation compared with ZsGreen^+^ B6 controls (Extended Data Fig. 9f). Thus, although limited peptide–MHC transfer may occur, the majority of CD8 T cell activation must be due to BMDM processing and presentation of ingested cytosolic antigen.

### Diverted routing tied to less priming

**Fig. 5:**
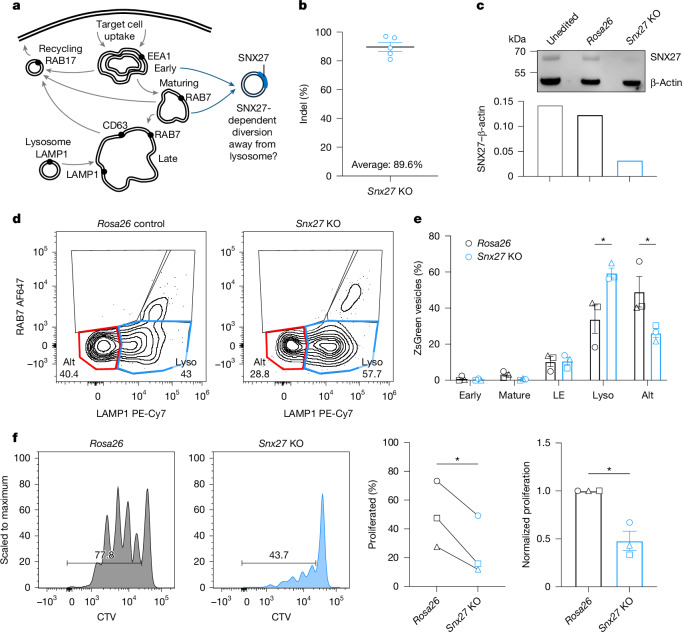
*Snx27* KO in macrophages increases antigen trafficking to lysosomes and decreases CD8 T cell activation. **a**, Schematic illustrating SNX27-dependent diversion of material away from lysosomal maturation into an alternative endocytic pathway. **b**,**c**, Validation of *Snx27* KO in BMDMs through indel quantification by inference of CRISPR edits (ICE) analysis (**b**; *n* = 5 biological replicates, *n* = 1 technical replicate per biological replicate, mean ± s.d.) and western blotting (**c**; image representative of two experiments). β-Actin was probed on the same gel as a loading control. For gel source data, see Supplementary Fig. 1. **d**,**e**, CRISPR–Cas9-edited BMDMs were co-cultured with ZsGreen target cells (B16) to evaluate ZsGreen trafficking after *Snx27* KO. Flow cytometry (gated on ZsGreen^+^ intracellular vesicles; **d**) and quantification of ZsGreen^+^ vesicles (**e**). *n* = 3 biological replicates (*n* = 1 technical replicate per biological replicate). *P* = 0.0011 for lysosomes (Lyso); *P* = 0.0032 for Alt. Shown are the mean of biological replicates ± s.e.m. Two-way ANOVA with Šidák’s multiple comparison. *P < 0.05. **f**, Effect of *Snx27* KO in BMDMs on CD8 T cell activation. Flow plots (left) and quantification (right) of OT-I CD8 T cell proliferation after 3 days of co-culture with FACS-isolated ZsGreen^+^ *Rosa26*-targeted and *Snx27* KO BMDMs that live-sampled B16-ZsGreen-minOVA target cells. Horizontal bars (flow plots) show percentage of proliferated CD8 T cells. *n *= 3 biological replicates. *P *= 0.0359 for percentage proliferated. *P* = 0.0351 for normalized proliferation. Each point represents one biological replicate (mean of *n* = 3 technical replicates). Shown are the mean of biological replicates ± s.e.m. Two-tailed paired *t*-test. *P < 0.05. Source data

**Extended Data Fig. 10:**
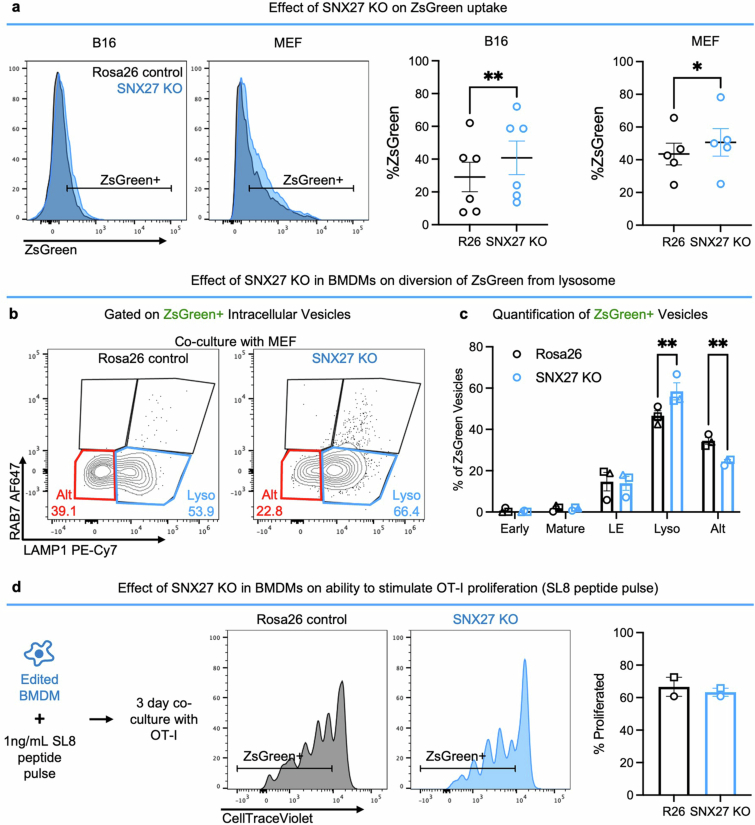
SNX27 KO reduces antigen diversion from the lysosome but not uptake or presentation capacity. **a**, ZsGreen uptake in SNX27 KO BMDMs compared to Rosa26 (R26) controls. n = 6 biological replicates for B16, n = 5 biological replicates for MEF. Each point represents one biological replicate (n = 1 technical replicate per biological replicate). Two-tailed paired t test; p = 0.0028 for B16, p = 0.0237 for MEF. **b**,**c** ZsGreen vesicle distribution in SNX27 KO BMDMs compared to Rosa26 (R26) controls after co-culture with MEFs. n = 3 biological replicates for (n = 1 technical replicate per biological replicate). Each point represents one biological replicate. Two-way ANOVA with Sidak’s multiple comparisons test; p = 0.0011 for Lyso; p = 0.0032 for Alt. **d**, OT-I proliferation after 3-day co-culture with peptide-pulsed SNX27 KO and Rosa26 control BMDMs. n = 2 biological replicates. Each point represents one biological replicate (mean of n = 3 technical replicates). Shown are mean of biological replicates +/− s.e.m. *p < 0.05, **p < 0.01. Source data

Material ingested through clathrin-independent, dynamin-independent-mediated endocytosis is diverted away from lysosomal degradation^27^, and the SNX27–retromer complex can prevent lysosomal delivery of ingested material^28^ (Fig. 5a). Because live sampling in our system is likewise independent of dynamin and leads to non-degradative trafficking, we examined how disruption of SNX27 affects antigen trafficking and subsequent T cell responses. CRISPR–Cas9-mediated targeting of the *Snx27* locus in BMDMs introduced insertions and deletions (indels) and decreased protein expression at high efficiency (Fig. 5b,c). *Snx27* KO did not impair ZsGreen sampling (Extended Data Fig. 10a). However, compared with *Rosa26*-targeted controls, *Snx27* KO increased ZsGreen trafficking to LAMP1^+^ lysosomes and reduced the fraction of ZsGreen localized to the alternative compartment (Fig. 5d,e and Extended Data Fig. 10b,c). This result is consistent with live-sampled material being diverted from the lysosome downstream of live sampling.

To determine whether these trafficking changes are associated with loss of cross-presentation, we co-cultured *Snx27* KO or control BMDMs with B16-ZsGreen-minOVA target cells and isolated ZsGreen^+^ BMDMs for co-culture with OT-I CD8 T cells. Consistent with increased antigen degradation, *Snx27* KO BMDMs induced less T cell proliferation (Fig. 5f). Notably, *Snx27* KO did not impair T cell proliferation when BMDMs were pulsed with SL8 peptide (Extended Data Fig. 10d). This outcome confirmed that the observed effect is due to altered antigen processing rather than general defects in APC functionality.

## Discussion

Together, these findings demonstrate that there is substantial sampling of live cells that takes place via a trogocytosis-like mechanism and fills a distinct vesicular compartment that is particularly underrepresented in late endosomes^10^. That macrophages can constitutively ingest small amounts of material from live cells is in itself unsurprising. Indeed, a pruning function for macrophages has been demonstrated to be important to activate stem cells^29^ and to optimize brain circuitry via small synaptic pruning^30^. A key distinction here is that we revealed the occurrence of very low-volume trogocytosis-like sampling that may be ongoing at steady state in a variety of cells and may be a significant source of self-antigen from healthy cells. This process provides macrophages with a distinct endocytic trafficking mechanism with which to present healthy self-antigens, notably to CD8 T cells. We also note a single previous observation of trogocytosis by dendritic cells that focused on a more conventional ‘ripping’ mechanism that leads to direct transfer of pre-formed peptide–MHC from the donor cell membrane^26^. By contrast, using previously unavailable vesicle cytometry, our results reveal the maintenance of sampled material in a depot that can be processed by the APC for presentation. A more stable depot of self-antigens provides a means of integrating and presenting the self-identity of many sampled cells, perhaps over days or longer. The longer retention of material sampled by this trogocytosis-like mechanism may contribute to a transferable pool of these vesicles, which has been observed to be exchanged among myeloid cells^13^. We note that we used model antigen and transgenic T cells in our study to reveal the capability of macrophages to cross-present live-cell-associated antigen by stimulating T cell proliferation. Therefore, the functional role of this cross-presentation mechanism for maintaining or tuning homeostatic T cell populations that have undergone thymic self-tolerance requires further investigation. Ultimately, this work provides a framework to understand how the immune system can accumulate information about our healthy self. This system can tune T cells to the normal concentrations of self-protein; however, it may also be exploited by tumour cells to promote pathological outcomes.

## Methods

No sample size calculations were performed before the study. Sample sizes were determined to be acceptable based on the magnitude of effect size from previous or preliminary experiments. Experiments were not randomized. Investigators were not blinded to group allocation during data collection.

### Mice

Mice were housed and bred under specific pathogen-free conditions, maintained on a 12–12 h light–dark cycle, controlled temperature of 20–26 °C and humidity of 30–70%, at the University of California, San Francisco (UCSF) Laboratory Animal Research Center. All experiments conformed to ethical principles and guidelines approved by the UCSF Institutional Animal Care and Use Committee, the National Institutes of Health and the American Association of Laboratory Animal Care. C57BL/6 (RRID: MGI:2159769), Ai6 (ref. ^31^) (mTmG), B6;129P2-*Fcer1g*^*tm1Rav*^/J (*Fcer1g* KO, RRID: MGI:2162818)^32^ and BALB/c (RRID: MGI:2161072) mice were purchased from The Jackson Laboratory or bred in-house. Both male and female mice ranging from 6 to 20 weeks old were used for experiments. Food and water were provided ad libitum.

To generate ZsGreen reporter mice, Ai6 mice were crossbred with *K14*^*cre*^, *Scgb1a1*^*creERT2*^, *Vil1*^*cre*^ or *Actb*^*cre*^ mice. To induce ZsGreen expression in *Scgb1a1*^*creERT2*^;Ai6 mice, mice were fed tamoxifen-containing chow ad libitum for 2 weeks.

For tumour studies, B16-F10 melanoma cancer cells were resuspended in PBS and mixed at a 1:1 (v:v) ratio with growth-factor-reduced Matrigel matrix (BD Biosciences), and 100,000 cells in 50 µl volume were transplanted into the subcutaneous region of the mouse flank. On day 14 after tumour challenge, when tumours reached a size or volume of approximately 0.5 cm^3^, mice were euthanized, tumours were excised and processed for downstream analyses in accordance with the UCSF Institutional Animal Care and Use Committee.

### Tumour cell lines

B16-F10 cells were purchased from the American Type Culture Collection (CRL-6475). B16-ZsGreen and B16-ZsGreen-minOVA cell lines have been previously described^13^. In brief, to make these cell lines, B16-F10 melanoma parental cells were genetically engineered through viral transduction with a ZsGreen or ZsGreen-minOVA construct. B16-GC3AI cell lines were genetically engineered to stably express the GFP-CASP3-activity indicator through viral transduction with an Addgene construct (78910). Cell lines were not authenticated. Cell lines were confirmed negative for mycoplasma using a MycoAlert Mycoplasma Detection kit (Lonza, LT07-118). Adherent cell lines were cultured at 37 °C in 5% CO_2_ in DMEM (Invitrogen), 10% FCS (Benchmark) and 100 U ml^–1^ penicillin, 100 mg ml^–1^ streptomycin and 2 mM l-glutamine, (pen–strep–glut; Invitrogen).

### MEFs

MEFs were derived from *Actb*^*cre*^;Ai6 mice that were generated by crossing *Actb*^*cre*^ mice (strain 033984, The Jackson Laboratory) to Ai6 mice (strain 007906, The Jackson Laboratory) as previously described^33^. In brief, day 13.5 embryos were collected from pregnant females, and the embryos were minced and digested with trypsin. The retrieved cells were washed and plated in DMEM (Invitrogen), 15% FCS (Benchmark) and pen–strep–glut (Invitrogen) for overnight culture at 37 °C in 5% CO_2_. The medium was aspirated after 24 h to remove any cells remaining in suspension and replaced with fresh medium. Cells were then grown to 70–80% confluency and cryopreserved.

### Tissue digest and flow cytometry staining

#### Lung

Lungs were collected from mice after euthanasia by an overdose of 2.5% Avertin. Lungs were placed in 3 ml DMEM (Gibco) in C-Tubes (Miltenyi) and briefly processed with a GentleMACS dissociator (Miltenyi). Next, 2 ml DMEM with 0.26 U ml^−1^ Liberase (Roche) and 0.25 mg ml^–1^ DNase I (Roche) were subsequently added and samples were incubated at 37 °C in a shaker for 30 min and dissociated to single-cell suspensions by GentleMACS. Tissue homogenate was then passed through a 100 mm filter. Red blood cells were lysed with 3 ml RBC lysis buffer (155 mM NH_4_Cl, 12 mM NaHCO_3_ and 0.1 mM EDTA) per lung for 5 min at room temperature. Samples were then washed with FACS buffer (2% FBS and 2 mM EDTA in PBS) and resuspended in appropriate buffer for staining for flow cytometry or FACS.

#### Tumour

For tumour digests, tumours from mice were collected 14 days after injection. Tumours were minced and incubated in digestion buffer (100 U ml^–1^ collagenase type I (Roche), 500 U ml^–1^ collagenase type IV (Roche) and 200 mg ml^−1^ DNAse I (Roche) in RPMI-1640 (Gibco)) for 30 min on a shaker at 37 °C. Digestion mixtures were then pipetted repeatedly, followed by another 15-min incubation at 37 °C. Cells were quenched with RPMI-1640 (Gibco) plus 10% FCS, washed with FACS buffer and filtered through a 100 mm cell strainer before staining for flow cytometry.

#### LNs

Inguinal LNs were dissected from mice, cleaned of fat and digested as previously described^14^. In brief, LNs were pierced and torn with sharp forceps in 24-well plates and incubated for 15 min at 37 °C in 1 ml digestion buffer (100 U ml^–1^ collagenase type I (Roche), 500 U ml^−1^ collagenase type IV (Roche) and 20 μg ml^–1^ DNAse I (Roche) in RPMI-1640 (Gibco)). Cells were pipetted up and down repeatedly, followed by another 15-min incubation at 37 °C. After digestion, LNs were washed with RPMI-1640 (Gibco) plus 10% FCS, washed with FACS buffer and filtered through 70 μm Nytex filters before staining for flow cytometry.

### Imaging sample preparation, image acquisition and image analysis

#### Two-photon imaging of mouse lung, skin, tumour and gut slices

Imaging of lung, skin, tumour and gut slices were performed using a custom-built two-photon setup equipped with two infrared lasers: MaiTai (Spectra Physics) and Chameleon (Coherent). The Chameleon laser was set to 950 nm for excitation of ZsGreen. Emitted light was detected using a ×25, 1.2 NA water lens (Zeiss) coupled to a 6-colour detector array (custom, using Hamamatsu H9433MOD detectors). Emission filters used were blue 475/23, green 510/42, yellow 542/27, red 607/70 and far-red 675/67. The microscope was controlled using the MicroManager software suite, and time-lapse *z*-stack images were acquired every 90 s with fivefold averaging and a *z*-step of 4 mm. Data analysis was performed with Imaris software (Bitplane).

For lung slices, mice were euthanized by anaesthetic overdose with 1 ml 2.5% Avertin and then intubated by tracheotomy with the sheath from an 18-gauge intravenous catheter. Lungs were subsequently inflated with 1 ml of 2% low-melting agarose (BMA) in sterile PBS at 37 °C. Agarose was then solidified by flooding the chest cavity with 4 °C PBS. Inflated lungs were excised, and the left lobe was cut into 300 mm sections using a vibratome. For skin, tumour and gut slices, mice were euthanized using CO_2_, obstructing fat was removed and tissue sections were embedded in 4% low-melting agarose in PBS before sectioning. Sections were mounted on plastic coverslips and imaged by two-photon microscopy at 37 °C in RPMI-1640 medium (Gibco, without Phenol Red) perfused with carbogen (5% CO_2_ and 95% O_2_) in a heated chamber.

#### Spinning disc confocal microscopy

Glass-bottom 96-well plates were coated in fibronectin and washed as described above. BMDMs, sorted ZsGreen^+^ BMDMs after antigenic transfer and unsorted antigenic transfer assays were imaged. Live imaging by spinning disc confocal microscopy was performed at 37 °C with 488 nm and 561 nm lasers at 40% and 50% laser power, respectively. For LAMP1 staining, cells were fixed with 4% paraformaldehyde at room temperature for 15 min, permeabilized with 0.5% saponin at room temperature for 15 min, incubated with blocking buffer (1% BSA, 0.1% saponin and 5% normal rat serum) at room temperature for 60 min and incubated with LAMP1-AF647 at 1:250 dilution in blocking buffer at 4 °C overnight. Fixed cells were imaged with 488 nm and 640 nm lasers at 25% laser power.

#### NSPARC imaging

Glass-bottom 96-well flat-bottom plates were coated with 50 µg ml^–1^ fibronectin in H_2_O at 37 °C for 1 h, or at 4 °C overnight, before use. Fibronectin-coated wells were washed twice with PBS before use. Next, 8 × 10^3^ tdTomato^+^ BMDMs were plated with 1.2 × 10^4^ ZsGreen^+^ B16-F10 cells or ZsGreen^+^ MEFs and spun at 1,500*g* for 5 min. Cells were incubated at 37 °C for 2 h before imaging. NSPARC imaging was performed at 37 °C with 488 nm and 560 nm lasers with 1.0% and 2.0% laser power, respectively.

#### Lattice light-sheet microscopy

In brief, 5 mm round coverslips were cleaned using a plasma cleaner and coated with 2 μg ml^–1^ fibronectin in H_2_O at 37 °C for 1 h, or at 4 °C overnight, before use. Fibronectin-coated coverslips were washed twice with PBS before use. Cells were plated onto fibronectin-coated coverslips 20 min before imaging with a 10-min spin at 1,400 rpm and 4 °C. Coverslip was immediately loaded into the sample bath with warmed imaging medium and secured. Imaging was performed at 37 °C with 488 nm and 560 nm lasers (MPBC). The exposure time was 10 ms per frame, which led to a temporal resolution of around 4.5. The lattice light-sheet microscope used was a homebuilt clone of a previously described microscope^34^ with a Nikon CFI Apo LWD ×25 W 1.1 NA 2 mm working distance objective, a Hamamatsu Orca flash 4.0 (v.2) camera and custom LabView acquisition software. This method was derived from previous work^19^.

#### Image analysis

All computational image analyses were performed in Imaris (v.9.9.1 or v.10.2.0, Bitplane) and Fiji (v.2.16.0/1.54p). Particle area analysis was performed using the Analyze particles function in Fiji with the following parameters: size (.027- infinity) and circularity (0.00–1.00). Post-processing raw data from lattice light-sheet images were deconvoluted using iterative Richardson–Lucy deconvolution as implemented in LLSpy. In brief, images were deconvolved with a known point spread function that was recorded for each colour before the experiment, as previously described^19^. A typical sample area underwent 15–20 iterations of deconvolution. For live-imaging experiments, photobleaching correction was applied in Fiji using the histogram-matching method. The tdTomato channel was scaled by a factor of 100 using channel arithmetic in Imaris before surface generation for NSPARC image analysis. Particle volume was measured using the object-object statistics function in Imaris from the separated vesicle in frames 13–18.

For ZsGreen colocalization analysis with LAMP1, Mander’s coefficients were calculated using the JACoP plugin in ImageJ^35^. Otsu thresholding was used to identify optimal thresholding for the phagocytosis condition. To account for the low fluorescence intensities of ZsGreen vesicles in the live-sampling condition, we opted for a manual threshold level of 550. Thresholding results were confirmed by comparing to ZsGreen^–^ BMDMs.

### Flow cytometry and FACS

A Zombie NIR Fixable Viability kit (423106; BioLegend), DAPI or propidium iodide was used for exclusion of dead cells. Surface staining was performed with anti-mouse Fc receptor antibody (clone 2.4G2, UCSF Hybridoma Core) in FACS buffer for 30 min on ice. Supplementary Table 1 lists all the antibodies referenced for flow cytometry and imaging experiments. Apoptotic cells were detected by staining with Annexin V AF647 (BioLegend, 649012) and 1 μg ml^–1^ DAPI in Annexin V binding buffer (BioLegend, 422201). For all experiments that involved intracellular staining, BD Cytofix/Cytoperm (554722) was used. Following fixation and permeabilization, cells were incubated with Fc block for 10 min on ice before the addition of intracellular stain. Flow cytometry was performed on a BD Fortessa instrument, and sorting was performed on BD FACSAria or BD FACSAria Fusion instruments. FacsDiva (v.9.0) and SpectroFlo (v.3.3) were used for collecting flow cytometry data, and FlowJo (v.10 software, BD Biosciences) was used for all analyses.

### Generation of BMDMs

In brief, 6–12-week-old C57BL/6 mice were euthanized and their femurs and tibiae were excised. Bone marrow was crushed using a mortar and pestle. After pelleting the bone marrow, the red blood cells were lysed using RBC lysis buffer for 5 min at room temperature. Cells were washed with FACS buffer and filtered through a 100 mm cell strainer before seeding at 1 × 10^6^ cells per ml on a low-adherent cell culture dish in BMDM medium (DMEM supplemented with 10% FBS (Benchmark), 50 mM β-mercaptoethanol, pen–strep–glut (Invitrogen) and 20 ng ml^−1^ M-CSF (Peprotech)). Fresh BMDM medium was added on day 3–4 of culture. On day 6–7 of culture, BMDMs were collected and used for experiments.

### In vitro antigen transfer assay

BMDMs were isolated as described above and co-cultured with target B16-ZsGreen cells or MEF-ZsGreen cells at a 1:1 ratio for 16 h before assays unless otherwise noted. Cells were plated in BMDM medium in tissue-culture-treated 96-well flat-bottom plates before staining and analysis by flow cytometry.

### Drug inhibitor antigenic transfer assay

Antigenic transfer assays were performed as described above. For all experiments for which apoptosis was induced, B16-F10 cells or MEFs were treated with 1 μM staurosporine (Tocris Bioscience, 1285) and washed with PBS before antigenic transfer assays. For exosome and microparticle inhibitory experiments, 5-(*N*,*N*-dimethyl)amiloride hydrochloride (DMA, Sigma Aldrich, A4562) was plated at 0 h to a final concentration of 10 μM. For endocytic inhibition experiments, dynole 34-2 (Cayman Chemical, 34073) was plated at 0 h to a final concentration of 5 μM. Caspase inhibition was performed using zVAD-FMK (Invivogen, tlrl-vad) at a final concentration of 20 μM for B16-ZsGreen or 30 μM for MEF-ZsGreen at 0 h. Signalling and cytoskeleton inhibition were performed using 10 μM PP1 (Cayman,14244), 5 μM piceatannol (MedChemExpress, HY-13518), 10 μM GDC-0941 (Selleck Chemicals, S1065), 1.35 μM NAV-2729 (MedChemExpress, HY-112473), 25 μM NSC23766 (MedChemExpress, HY-15723), 10 μM ZCL278 (MedChemExpress, HY-13518), 200 μM CK-666 (Sigma-Aldrich, SML0006-5MG) or 5 μM SMIFH2 (MedChemExpress, HY-16931).

### Drug inhibitor functional validation and cell toxicity validation

For exosome and microparticle inhibitor validation, target cells were cultured with DMA and supernatant was collected to quantify ZsGreen^+^ vesicles by small-particle flow cytometry. For endocytosis inhibitor validation, exosomes were isolated from confluent B16-ZsGreen cultures using an ExoQuick kit (System Biosciences, EXOA5A-1), and BMDMs were cultured with exosomes and dynole 34-2 or DMSO control. Exosome endocytosis was quantified by flow cytometry. For apoptosis inhibition studies, B16-ZsGreen or MEF-ZsGreen target cells were pre-treated with zVAD for 1 h before staurosporine treatment. The percentage of AnnexinV^+^DAPI^+/−^ apoptotic cells was evaluated by flow cytometry. For toxicity studies, cells were treated with drug or vehicle control for 16 h and quantified by flow cytometry with CountBright beads (Invitrogen, C36950).

### Supernatant–Transwell antigenic transfer assay

Medium was replaced for B16-ZsGreen cells and MEF-ZsGreen cells after reaching 80% confluency. After 48 h, supernatants were collected and used in antigenic transfer assays as described above. Transwell experiments were performed as described for the antigen transfer assays with a 3 μm pore Transwell insert separating BMDMs and ZsGreen^+^ target cells or supernatant.

### Antibody opsonization and blockade

For opsonization experiments, B16-ZsGreen and MEF-ZsGreen target cells were pre-coated with 10 µg ml^–1^ Armenian hamster IgG (BD Pharmigen, 553969), 10 μg ml^–1^ rat IgG2ak (clone 2A3, invivoMab, BE0089), 10 μg ml^–1^ CD29 (eBioHmb1-1, eBioscience, 16-0291-85), 10 μg ml^–1^ anti-CD98 (RL388, BioLegend, 128202), normal mouse serum (Jackson ImmunoResearch, 015-000-120) or 500 μg ml^–1^ IgG from normal mouse serum at 37 °C for 30–60 min and washed with PBS before co-culture with BMDMs. IgG from normal mouse serum was isolated using Protein G Dynabeads (Invitrogen, 10003D) according to the manufacturer’s instructions and dialysed with PBS (Slide-A-Lyzer). The concentration of isolated IgG was quantified using IgG ELISA (Abcam, ab151276).

For antibody blockade, BMDMs were pre-treated with 10 μg ml^–1^ rat IgG2b (clone MPC-11, invivoMab, BE0086), CD16/32-blocking antibody (clone 2.4G2), CD11b-blocking antibody (clone M17/0, BioLegend, 101202, RRID: AB_312785), CD49e-blocking antibody (clone 5H10-27(MRF5), BioLegend, 103801, RRID: AB_313050), SIRPα-blocking antibody (clone P84, BioLegend, 144035, RRID: AB_2832516) or custom afucosylated mouse IgG2a monoclonal blocking antibody against TREM2 (provided by T. Courau, synthesized by Evitra) for 1 h before 16 h of co-culture antigen transfer assays.

### CRISPR editing of primary BMDMs

CRISPR editing of primary BMDMs were performed as previously described^36^. In brief, single guide RNA (sgRNA) targeting *Rosa26* (5′-ACUCCAGUCUUUCUAGAAGA-3′), *Fcgr1* (5′-GAUCACCUUGCAGCCUCCAU-3′), *Fcgr3* (5′-UGGUGAAACUGGACCCCCCA-3′), *Fcgr4* (5′-GGUGAACCUAGACCCCAAGU-3′), *Itgam* (5′-GAAGCCAUGACACAAGGCUA-3′), *Itgav* (5′-UUGAAUCAAACUCAAUGGGC-3′, 5′-CCUGUUGAAUCAAACUCAAU-3′), *Tlr2* (5′-UUGGCUCUUCUGGAUCUUGG-3′), *C5ar1* (5′-CAUGGAUCCUAACAUACCUG-3′, 5′-GAUCCUAACAUACCUGCGGA-3′, 5′-AUGGCAUUCACCUCCCGAAG-3′), *Cd93* (5′-CAGGAACAAACCAGUUGAGA-3′, 5′-AGAAGAAUGGCCAUCUCAAC-3′, 5′-CUGGUUUGUUCCUGCUGCUG-3′) and *Snx27* (5′-GGAACGGCGUGAAUGUUGAG-3′, 5′-UGAGGGGGCGACACACAAGC-3′, 5′-GUGGUGGACCUGAUCCGAGC-3′) were ordered from Synthego or IDT and reconstituted to 100 µM in TE (IDT). sgRNA and Cas9 (6.5 mg ml^–1^) was complexed in a 2:1 molar ratio at room temperature for at least 10 min. At day 3 after initial plating, BMDM differentiation cultures were collected, and cells were washed with PBS before electroporation with ribonucleoprotein containing gene-targeting sgRNA complexed in P3 primary cell solution with electroporation code CM137 using a Lonza 4D Nucleofector. After an additional 4 days of differentiation, gene-edited BMDMs were either stained with antibodies for validation of protein knockdown or co-cultured with ZsGreen target cells for 16 h before evaluation by flow cytometry. For indel quantification with ICE, DNA was extracted using QuickExtract for PCR of the targeted locus with Phusion Plus Green PCR master mix (Thermo Fisher Scientific, F632S) and primers (5′-CCCCATCTTTCCCACATGCT-3′, 5′-ATTACTGTAGGCCACCCCCT-3′). Guide number and selection for targeting each gene were empirically determined based on protein knockdown or indel frequency.

### NicheNet analysis to predict receptors

To identify potential BMDM surface proteins that mediate live sampling using NicheNet^22^, we used the following previously published datasets: BMDM datasets^37^, a B16 dataset^38^ and a MEF dataset^39^. The BMDM was set as the receiver population and B16 and MEF were set as sender populations. A threshold for expressed genes for each population was defined based on a Gaussian fit of gene expression, and BMDM receptors were prioritized if they were predicted to interact with both cell types, overlap with known phagocytic modulators and confirmed to be expressed at the protein level.

### Vesicle flow cytometry

#### Sample preparation and sorting

In brief, 16 h before sorting, BMDMs were cultured with live target cells (trogocytosis) in a 1:1 ratio, with staurosporine-treated target cells (phagocytosis) in a 1:1 ratio or with supernatant derived from target cells (endocytosis). Cells were collected, washed with FACS buffer, stained with CD45-BUV395 with FcBlock for 30 min on ice, washed with FACS buffer and resuspended in FACS buffer with 1 mg ml^–1^ propidium iodide at 10 × 10^6^ cells per ml. CD45^+^ cells were sorted on FACSAria and FACSFusion machines on ‘Purity’ into FACS buffer kept at 4 °C.

#### Surface biotinylation and lysis

Cells were washed 3 times in PBS, resuspended in PBS at 25 × 10^6^ cells per ml then biotinylated with 80 µl of 10 mM EZ-Link Sulfo-NHS-SS-Biotin (Thermo Fisher Scientific, 21331) per ml of reaction volume for 30 min at 4 °C. Cells were washed 3 times in PBS, resuspend in at least 500 μl homogenization buffer (250 mM sucrose, 200 mM PMSF, 250 mM sucrose, 3 mM imidazole and 1× protease inhibitor filtered through a 0.1 µm filter) at a concentration of 10 × 10^6^ cells per ml and drawn 15 times up and down through a 22 gauge needle. The cell homogenate was spun for 4 min at 150*g* and post-nuclear supernatant was spun at 3,000*g* for 5 min at 4 °C to collect vesicles.

#### Staining

All staining reagents were filtered using a 0.1 μm filter before use and vesicles were pelleted using spin speeds of 3,000*g* for 5 min at 4 °C for all steps. Vesicles were stained with 50 μM CellTraceViolet (Thermo Fisher Scientific, C34557) for 5 min at room temperature and washed once with PBS. Samples were fixed for 20 min at room temperature with fixation buffer (Invitrogen, 88-8824-00), washed with permeabilization buffer (Invitrogen, 88-8824-00) twice and stained in 50 μl of permeabilization buffer with 1% FCS with FcBlock for 30 min at room temperature. Vesicles were washed with permeabilization buffer and resuspend in 100 μl PBS for acquisition.

#### Sample acquisition and analysis

Aurora Cytek flow cells were soaked with contrad overnight with long clean before acquisition with filtered reagents. Calibration beads and samples were acquired on an Aurora Cytek with Enhanced Small Particle detection with SpectroFlo software.

### T cell stimulation assays

ZsGreen^+^ BMDMs were sorted from BMDMs after 16 h of co-cultures with DMSO-treated or staurosporine-pre-treated B16-zsGreen-minOVA cells. To induce MHC-II expression on BMDMs, sorted cells were stimulated with 20 ng ml^–1^ IFNγ for 2 h and then washed before co-culture with T cells. OT-I and OT-II T cells were isolated from spleens of TCR transgenic mice after red blood cell lysis using either a CD8 or CD4 EasySep enrichment kit (Stemcell Technologies), respectively. T cells were labelled with CellTraceViolet (Thermo Fisher Scientific) at 37 °C in PBS for 15 min and washed in RPMI before use. T cell stimulation assays were performed as previously described^14^. In brief, sorted BMDMs and T cells were added to the wells of a 96-well V-bottom plate at a 1:3 ratio in RPMI (Gibco) supplemented with 10% FCS (Benchmark), pen–strep–glut (Invitrogen) and 50 mM β-mercaptoethanol (Thermo Fisher Scientific). Cells were collected for analysis 3 days later. Dilution of the cell-permeable dye CellTraceViolet and expression of CD69 were used as indicators of T cell stimulation.

### Western blotting

Whole-cell protein lysates were obtained from BMDMs in Pierce RIPA buffer (Thermo Fisher Scientific, 89901) and ProteoGuard EDTA-free protease inhibitor cocktail (Takara, 635673). Lysates were extracted via sample agitation at 4 °C for 30 min, followed by 4 °C centrifugation at 16,000 rpm for 20 min. The protein concentration was determined using a Pierce BCA Protein Assay kit (Thermo Fisher Scientific). Lysates were denatured in NuPAGE LDS sample buffer (Invitrogen, NP0007) supplemented with NuPAGE sample reducing agent (Invitrogen, NP0009) by incubating at 95 °C for 5 min. Denatured samples were loaded onto a NuPAGE 10% (NP0302BOX) or 4–12% Bis-Tris (NP0323BOX) polyacrylamide gel (Thermo Fisher Scientific) with PageRuler Plus pre-stained protein ladder (Thermo Fisher Scientific, 26619) and electrophoresed in MES running buffer (Invitrogen). Gels were transferred to PVDF membranes using an iBlot 2 system (Invitrogen, IB21001), blocked with Pierce clear milk blocking buffer (Thermo Fisher Scientific, 37587) and incubated with primary antibodies according to the manufacturers’ directions. Primary antibodies, SNX27 (Abcam, clone: EPR218130-16, ab315897, 1:1,000), and β-actin (Invitrogen, PA1-183, RRID: AB_2539914, 1:5,000) were detected with goat anti-rabbit IgG HRP-conjugated secondary antibodies (Southern Biotech, 4050-05, RRID: AB_2795955, 1:1,000) and SuperSignal West Femot maximum sensitivity substrate (Thermo Fisher Scientific, 34095). Membranes were imaged using a Licor Odyssey XF system.

### Quantification and statistical analysis

Unless specifically noted, all data are representative of >3 separate experiments. Experimental group assignment was determined by genotype or, if all wild-type mice, by random designation. Statistical analyses were performed using GraphPad Prism software. Error bars represent the s.e.m. calculated using Prism and are derived from triplicate or greater experimental conditions. Specific statistical tests used were paired and unpaired *t*-tests, and *P* < 0.05 was considered significant. For pairwise comparisons, unpaired *t*-tests were used unless otherwise noted. For statistical measures between more than two groups, one-way ANOVA, two-way ANOVA and mixed-effect models were performed unless otherwise noted. Investigators were not blinded to group assignment during experimental procedures or analysis.

### Materials availability

Requests for resources and reagents should be directed to and will be fulfilled by the lead contacts, A.C.F. and M.F.K.

### Reporting summary

Further information on research design is available in the Nature Portfolio Reporting Summary linked to this article.

## Online content

Any methods, additional references, Nature Portfolio reporting summaries, source data, extended data, supplementary information, acknowledgements, peer review information; details of author contributions and competing interests; and statements of data and code availability are available at 10.1038/s41586-026-10435-5.

## Supplementary information

Supplementary Figures This file contains Supplementary Figs. 1–5.

Reporting Summary

Supplementary Table 1 Flow cytometry and imaging antibodies.

Peer Review File

Supplementary Video 1 NSPARC confocal live imaging captures trogocytosis-like sampling. Three-dimensional surface rendering of confocal live imaging of tdTomato^+^ BMDM and ZsGreen^+^ B16-F10 interactions using the NSPARC detector, visualizing the formation and separation of a vesicle from a ZsGreen^+^ B16 cells into a tdTomato^+^ BMDM. Time shown as min:s. *z*-stacks within series were captured in 30-s intervals.

Supplementary Video 2 Lattice light-sheet imaging captures trogocytosis-like sampling. Three-dimensional surface rendering of lattice light-sheet imaging of tdTomato^+^ BMDMs and ZsGreen^+^ B16-F10 cells capturing the stretching and separation of a ZsGreen^+^ vesicle from a target cell. Time shown as min:s.

## Source data

Source Data Fig. 1

Source Data Fig. 2

Source Data Fig. 3

Source Data Fig. 4

Source Data Fig. 5

Source Data Extended Data Fig. 1

Source Data Extended Data Fig. 2

Source Data Extended Data Fig. 4

Source Data Extended Data Fig. 5

Source Data Extended Data Fig. 6

Source Data Extended Data Fig. 7

Source Data Extended Data Fig. 8

Source Data Extended Data Fig. 9

Source Data Extended Data Fig. 10

## Data Availability

All data supporting the findings of this study are included in the Article and its associated Supplementary Information. Additional data supporting the findings of this study are available from the lead contacts upon reasonable request. Transcriptomic data analysed in this study were obtained from the NCBI Gene Expression Omnibus database (accession numbers GSE99759, GSE155972 and GSE171127). Source data are provided with this paper.
