# Supplementary material for: Submicrometre sampling of living cells by macrophages
Source: Nature. 2026 Apr 29;654(8118):495–503. doi: 10.1038/s41586-026-10435-5 (PMC13253326; doi:10.1038/s41586-026-10435-5)
Supplement: Supplementary file 2 — Reporting Summary [file 41586_2026_10435_MOESM2_ESM.pdf]

nature portfolio

Corresponding author(s): Matthew F. Krummel, Amy C. Fan

Last updated by author(s): Feb 17, 2026

Reporting Summary

Nature Portfolio wishes to improve the reproducibility of the work that we publish. This form provides structure for consistency and transparency in reporting. For further information on Nature Portfolio policies, see our Editorial Policies and the Editorial Policy Checklist.

Statistics

For all statistical analyses, confirm that the following items are present in the figure legend, table legend, main text, or Methods section.

n/a	Confirmed	
☐	☒	The exact sample size (n) for each experimental group/condition, given as a discrete number and unit of measurement
☐	☒	A statement on whether measurements were taken from distinct samples or whether the same sample was measured repeatedly
☐	☒	The statistical test(s) used AND whether they are one- or two-sided<br>*Only common tests should be described solely by name; describe more complex techniques in the Methods section.*
☐	☒	A description of all covariates tested
☐	☒	A description of any assumptions or corrections, such as tests of normality and adjustment for multiple comparisons
☐	☒	A full description of the statistical parameters including central tendency (e.g. means) or other basic estimates (e.g. regression coefficient) AND variation (e.g. standard deviation) or associated estimates of uncertainty (e.g. confidence intervals)
☐	☒	For null hypothesis testing, the test statistic (e.g. F, t, r) with confidence intervals, effect sizes, degrees of freedom and P value noted<br>*Give P values as exact values whenever suitable.*
☒	☐	For Bayesian analysis, information on the choice of priors and Markov chain Monte Carlo settings
☒	☐	For hierarchical and complex designs, identification of the appropriate level for tests and full reporting of outcomes
☒	☐	Estimates of effect sizes (e.g. Cohen's d, Pearson's r), indicating how they were calculated

Our web collection on statistics for biologists contains articles on many of the points above.

Software and code

Policy information about availability of computer code

Data collection	FacsDiva 9.0 and SpectroFlo 3.3 were used for collecting flow cytometry data.<br>MicroManager was used to collect two-photon data.<br>Nikon Elements was used to collect confocal imaging data.<br>Custom LabView acquisition software was used to collect lattice light-sheet microscopy data.
Data analysis	FlowJo v10 was used for analyzing flow cytometry data.<br>Statistical analysis performed in GraphPad Prism v10.<br>Image analysis performed in Fiji/ImageJ v2 and Imaris v9/10 software.

For manuscripts utilizing custom algorithms or software that are central to the research but not yet described in published literature, software must be made available to editors and reviewers. We strongly encourage code deposition in a community repository (e.g. GitHub). See the Nature Portfolio guidelines for submitting code & software for further information.

Data

Policy information about availability of data

All manuscripts must include a data availability statement. This statement should provide the following information, where applicable:

- Accession codes, unique identifiers, or web links for publicly available datasets
- A description of any restrictions on data availability
- For clinical datasets or third party data, please ensure that the statement adheres to our policy

All data supporting the findings of this study are included in the Article and its associated Supplementary. Source data underlying all main and supplementary figures are provided with this paper. Processed flow cytometry and imaging quantifications used for statistical analyses are included in the source data files. Additional data supporting the findings of this study are available from the lead contacts upon reasonable request. Transcriptomic data analyzed in this study were obtained from NCBI Gene Expression Omnibus (GEO) database, accession numbers GSE99759, GSE155972, and GSE171127.

Research involving human participants, their data, or biological material

Policy information about studies with human participants or human data. See also policy information about sex, gender (identity/presentation), and sexual orientation and race, ethnicity and racism.

Reporting on sex and gender	N/A
Reporting on race, ethnicity, or other socially relevant groupings	N/A
Population characteristics	N/A
Recruitment	N/A
Ethics oversight	N/A

Note that full information on the approval of the study protocol must also be provided in the manuscript.

Field-specific reporting

Please select the one below that is the best fit for your research. If you are not sure, read the appropriate sections before making your selection.

☒ Life sciences ☐ Behavioural & social sciences ☐ Ecological, evolutionary & environmental sciences

For a reference copy of the document with all sections, see nature.com/documents/nr-reporting-summary-flat.pdf

Life sciences study design

All studies must disclose on these points even when the disclosure is negative.

Sample size	No sample size calculations were performed prior to study. Sample size was determined to be acceptable based on magnitude of efffect size from previous or preliminary experiments with an effort to achieve a minimum of n=3 biological replicates or mice per experiment. Specific sample sizes and independent experiments can be found in the figures, their accompanying legends, or within the methods section.
Data exclusions	Data exclusion was restricted to technical limitations of live cell imaging. Because cell-cell interactions are transient and may not occur within short imaging windows, direct visualization of interacting cells was not always achievable. Images lacking clear visualization of these interactions were excluded from analysis.
Replication	Not all imaging runs yielded observations of antigen uptake but all technically-successful imaging runs (5) were examined and imaging figures representative of the observed dynamics in all conditions of antigen-capture. All other experiments were independently replicated with at least 3 biological replicates and demonstrated the same phenotype unless noted.
Randomization	No assignment of participants/animals to experimental groups. Randomization was not applicable to all other experiments as experimental conditions were applied within independently generated biological replicates.
Blinding	Investigators were not blinded to group allocation during data collection. Blinding was not required, as data collection and analysis was based on objective quantitative readouts. Experiments were independently repeated by different investigators to ensure reproducibility.

Reporting for specific materials, systems and methods

We require information from authors about some types of materials, experimental systems and methods used in many studies. Here, indicate whether each material, system or method listed is relevant to your study. If you are not sure if a list item applies to your research, read the appropriate section before selecting a response.

Materials & experimental systems

n/a	Involved in the study
☐	☒ Antibodies
☐	☒ Eukaryotic cell lines
☒	☐ Palaeontology and archaeology
☐	☒ Animals and other organisms
☒	☐ Clinical data
☒	☐ Dual use research of concern
☒	☐ Plants

Methods

n/a	Involved in the study
☒	☐ ChIP-seq
☐	☒ Flow cytometry
☒	☐ MRI-based neuroimaging

Antibodies

Antibodies used

β-Actin Invitrogen, Cat #PA1-183, RRID: AB_2539914
CD103 APC 2E7 Biolegend Cat# 121413; RRID: AB_1227503
CD103 BUV737 2E7 BD Optibuild Cat# 749393 RRID: AB_2873763
CD103 PE 2E7 Biolegend Cat# 121406; RRID: AB_1133989
CD11b AF647 M1/70 Biolegend Cat# 101218; RRID: AB_389327
CD11b BV605 M1/70 Biolegend Cat# 101257; RRID: AB_2565431
CD11b BV650 M1/70 Biolegend Cat# 101239; RRID: AB_11125575
CD11b M17/0, Biolegend Cat# 101202, RRID: AB_312785
CD11c BV650 N418 Biolegend Cat# 117339; RRID: AB_2562414
CD11c PE-Cy7 N418 Biolegend Cat# 117318; RRID: AB_493568
CD16 (Fcgr3) PE S17014E Biolegend Cat# 158004; RRID: AB_2876540
CD16.2 (Fcgr4) PE-Cy7 9E9 Biolegend Cat# 149516; RRID: AB_2632749
CD18 AF647 M18/2 Biolegend Cat# 101414; RRID: AB_2265032
CD24 BV421 M1/69 Biolegend Cat# 101826; RRID: AB_2563508
CD24 PE-Cy7 M1/69 Biolegend Cat# 101822; RRID: AB_756048
CD282 (TLR2) Biotin 6C2 Thermo Scientific Cat# 13-9021-82
CD4 PE-Cy7 RM4-5 eBioscience Cat# 25-0042-82; RRID: AB_469578
CD45 AF647 30-F11 Biolegend Cat# 103124; RRID: AB_493533
CD45 AF700 30-F11 Biolegend Cat# 103128; RRID: AB_493715
CD45 BUV395 30-F11 BD Horizon Cat# 564279; RRID: AB_2651134
CD45 BV510 30-F11 Biolegend Cat# 103138; RRID: AB_2563061
CD45 BV605 30-F11 BD Horizon Cat# 563053; RRID: AB_2737976
CD45R (B220) BV785 RA3-6B2 Biolegend Cat# 103246; RRID: AB_2737976
CD45R (B220) PerCP-Cy5.5 RA3-6B2 Biolegend Cat# 103236; RRID: AB_893354
CD49e 5H10-27(MRF5), Biolegend Cat# 103801, RRID: AB_313050
CD51 (ITGAV) PE RMV-7 Biolegend Cat# 104105; RRID: AB_313074
CD63 PerCP-Cy5.5 NVG-2 Biolegend Cat# 143912; RRID: AB_2565502
CD64 (Fcgr1) APC X54-5/7.1 Biolegend Cat# 139305; RRID: AB_11219205
CD69 PE H1.2F3 Biolegend Cat# 104508; RRID: AB_313111
CD88 (C5AR1) PE-Cy7 20/70 Biolegend Cat# 135809; RRID: AB_10900077
CD8a BUV737 53-6.7 BD Horizon Cat# 564297; RRID: AB_2722580
CD8a PerCP-Cy5.5 53-6.7 Biolegend Cat# 100734; RRID: AB_2075238
CD90.2 AF700 30-H12 Biolegend Cat# 105320; RRID: AB_493725
CD90.2 AF647 30-H12 Biolegend Cat# 105318; RRID: AB_492888
CD90.2 BV785 30-H12 Biolegend Cat# 105331; RRID: AB_2562900
CD93 BV650 AA4.1 Biolegend Cat# 136517; RRID: AB_3698911
EEA1 AF594 G-4 Santa Cruz Cat# sc-137130 AF594; RRID: AB_2246349
F4/80 Biotin BM8 eBioscience Cat# 13-4801-82, RRID: AB_893499
Goat Anti-Rabbit IgG HRP Southern Biotech, Cat# 4050-05, RRID: AB_2795955
LAMP1 (CD107a) PE-Cy7 eBio1D4B eBioscience Cat# 25-1071-82; RRID: AB_2848304
Ly6C BV711 HK1.4 Biolegend Cat# 128037; RRID: AB_2562630
Ly6G BV785 IA8 Biolegend Cat# 127645; RRID: AB_2566317
MHC I (H2Kb) AF647 AF6-88.5 Biolegend Cat# 166511
MHC I (H2Kb) APC-eF780 AF6-88.5 eBioscience Cat# 47-5958-82; RRID: AB_2815170
MHC II (I-A/I-E) AF700 M5/114.15.2 Biolegend Cat# 107622; RRID: AB_493727
MHC II (I-A/I-E) BV785 M5/114.15.2 Biolegend Cat# 107645; RRID: AB_2565977
MHC II (I-A/I-E) BV421 M5/114.15.2 Biolegend Cat# 107631; RRID: AB_10900075
NK1.1 BV785 PK136 Biolegend Cat# 108749; RRID: AB_2564304
RAB17 PE-Cy5 Abcore Inc Cat# AC21-1439-15
RAB7 AF647 EPR7589 Abcam Cat# ab198737
Rat IgG2b MPC-11, invivoMab Cat# BE0086
SiglecF BV785 E50-2440 BD Optibuild Cat# 740956; RRID: AB_2740581
SIRPa P84, Biolegend Cat# 144035, RRID: AB_2832516
SNX27 EPR218130-16 Abcam Cat# ab315897
Streptavidin BV510 Biolegend Cat# 405234

	Streptavidin BV650 Biolegend Cat# 405231<br>VEGFR3 AF647 AFL4 Novus Cat# NBP1-43259AF647; RRID: AB_3208062

Validation

Antibodies were used to recognize the species and antigen reactivity in in applications (flow cytometry, immunofluorescence microscopy, and Western blotting) for which they are validated by the manufacturer, unless otherwise noted as validated by investigators in this study. Specificity was supported by appropriate experimental controls, including isotype controls for flow cytometry, expected molecular weight detection in Western blotting, and staining patterns consistent with known subcellular localization. For selected targets, specificity was further confirmed by reduced or absent signal in corresponding genetic knockout samples. Antibody dilutions were empirically optimized for the vesicle flow application.

APC anti-CD103 (Cat# 121413; RRID: AB_1227503) validated by manufacturer to recognize Mouse CD103
BUV737 anti-CD103 (Cat# 749393 RRID: AB_2873763) validated by manufacturer to recognize Mouse CD103
PE anti-CD103 (Cat# 121406; RRID: AB_1133989) validated by manufacturer to recognize Mouse CD103
AF647 anti-CD11b (Cat# 101218; RRID: AB_389327) validated by manufacturer to recognize Mouse and other species' CD11b
BV605 anti-CD11b (Cat# 101257; RRID: AB_2565431) validated by manufacturer to recognize Mouse and other species' CD11b
BV650 anti-CD11b (Cat# 101239; RRID: AB_11125575) validated by manufacturer to recognize Mouse and other species' CD11b
BV650 anti-CD11c (Cat# 117339; RRID: AB_2562414) validated by manufacturer to recognize Mouse CD11c
PE-Cy7 anti-CD11c (Cat# 117318; RRID: AB_493568) validated by manufacturer to recognize Mouse CD11c
PE anti-CD16 (Fcgr3) (Cat# 158004; RRID: AB_2876540) validated by manufacturer to recognize Mouse CD16
PE-Cy7 anti-CD16.2 (Fcgr4) (Cat# 149516; RRID: AB_2632749) validated by manufacturer to recognize Mouse CD16.2
AF647 anti-CD18 (Cat# 101414; RRID: AB_2265032) validated by manufacturer to recognize Mouse CD18
BV421 anti-CD24 (Cat# 101826; RRID: AB_2563508) validated by manufacturer to recognize Mouse CD24
PE-Cy7 anti-CD24 (Cat# 101822; RRID: AB_756048) validated by manufacturer to recognize Mouse CD24
Biotin anti-CD282 (TLR2) (Cat# 13-9021-82) validated by manufacturer to recognize Mouse CD282
PE-Cy7 anti-CD4 (Cat# 25-0042-82; RRID: AB_469578) validated by manufacturer to recognize Mouse and Human CD4
AF647 anti-CD45 (Cat# 103124; RRID: AB_493533) validated by manufacturer to recognize Mouse CD45
AF700 anti-CD45 (Cat# 103128; RRID: AB_493715) validated by manufacturer to recognize Mouse CD45
BUV395 anti-CD45 (Cat# 564279; RRID: AB_2651134) validated by manufacturer to recognize Mouse CD45
BV510 anti-CD45 (Cat# 103138; RRID: AB_2563061) validated by manufacturer to recognize Mouse CD45
BV605 anti-CD45 (Cat# 563053; RRID: AB_2737976) validated by manufacturer to recognize Mouse CD45
BV785 anti-CD45R (B220) (Cat# 103246; RRID: AB_2737976) validated by manufacturer to recognize Mouse and other species' CD45R
PerCP-Cy5.5 anti-CD45R (B220) (Cat# 103236; RRID: AB_893354) validated by manufacturer to recognize Mouse and other species' CD45R
PE anti-CD51 (ITGAV) (Cat# 104105; RRID: AB_313074) validated by manufacturer to recognize Mouse CD51
PerCP-Cy5.5 anti-CD63 (Cat# 143912; RRID: AB_2565502) validated by manufacturer to recognize Mouse CD63
APC anti-CD64 (Fcgr1) (Cat# 139305; RRID: AB_11219205) validated by manufacturer to recognize Mouse CD64
PE anti-CD69 (Cat# 104508; RRID: AB_313111) validated by manufacturer to recognize Mouse CD69
PE-Cy7 anti-CD88 (C5AR1) (Cat# 135809; RRID: AB_10900077) validated by manufacturer to recognize Mouse CD88
BUV737 anti-CD8a (Cat# 564297; RRID: AB_2722580) validated by manufacturer to recognize Mouse CD8a
PerCP-Cy5.5 anti-CD8a (Cat# 100734; RRID: AB_2075238) validated by manufacturer to recognize Mouse CD8a
AF647 anti-CD90.2 (Cat# 105318; RRID: AB_492888) validated by manufacturer to recognize Mouse CD90.2
AF700 anti-CD90.2 (Cat# 105320; RRID: AB_493725) validated by manufacturer to recognize Mouse CD90.2
BV785 anti-CD90.2 (Cat# 105331; RRID: AB_2562900) validated by manufacturer to recognize Mouse CD90.2
BV650 anti-CD93 (Cat# 136517; RRID: AB_3698911) validated by manufacturer to recognize Mouse CD93
AF594 anti-EEA1 (Cat# sc-137130 AF594; RRID: AB_2246349) validated by manufacturer to recognize Mouse and other species' EEA1. Flow application was validated in this study using KO and isotype controls.
Biotin anti-F4/80 (Cat# 13-4801-82; RRID: AB_893499) validated by manufacturer to recognize Mouse F4/80
PE-Cy7 anti-LAMP1 (CD107a) (Cat# 25-1071-82; RRID: AB_2848304) validated by manufacturer to recognize Mouse LAMP1
BV711 anti-Ly6C (Cat# 128037; RRID: AB_2562630) validated by manufacturer to recognize Mouse Ly6C
BV785 anti-Ly6G (Cat# 127645; RRID: AB_2566317) validated by manufacturer to recognize Mouse Ly6G
AF647 anti-MHC I (H2Kb) (Cat# 116511) validated by manufacturer to recognize Mouse H2kb
APC-eF780 anti-MHC I (H2Kb) (Cat# 47-5958-82; RRID: AB_2815170) validated by manufacturer to recognize Mouse H2kb
AF700 anti-MHC II (I-A/I-E) (Cat# 107622; RRID: AB_493727) validated by manufacturer to recognize Mouse MHC II (I-A/I-E)
BV421 anti-MHC II (I-A/I-E) (Cat# 107631; RRID: AB_10900075) validated by manufacturer to recognize Mouse MHC II (I-A/I-E)
BV785 anti-MHC II (I-A/I-E) (Cat# 107645; RRID: AB_2565977) validated by manufacturer to recognize Mouse MHC II (I-A/I-E)
BV785 anti-NK1.1 (Cat# 108749; RRID: AB_2564304) validated by manufacturer to recognize Mouse NK1.1
PE-Cy5 anti-RAB17 (Cat# AC21-1439-15) validated by manufacturer to recognize Mouse RAB17
AF647 anti-RAB7 (Cat# ab198737) validated by manufacturer to recognize Human Rab7 and predicted to recognize Mouse Rab7. Species-specific reactivity and flow application was validated in this study KO and isotype controls.
BV785 anti-SiglecF (Cat# 740956; RRID: AB_2740581) validated by manufacturer to recognize Mouse SiglecF
AF647 anti-VEGFR3 (Cat# NBP1-43259AF647; RRID: AB_3208062) validated by manufacturer to recognize Mouse VEGFR3
β-Actin (Invitrogen Cat #PA1-183, RRID: AB_2539914) validated by manufacturer to recognize Mouse and other species' β-Actin.
SNX27 (Abcam Cat# ab315897) validated by manufacturer to recognize Mouse and other species' SNX27.

Eukaryotic cell lines

Policy information about cell lines and Sex and Gender in Research

Cell line source(s)	B16-F10 cells used in this study were purchased from ATCC(ATCC, CRL-6475). B16-ZsGreen, B16-GC3AI, and B16-ZsGreen-minOVA lines were derived in-house using the methods described.
Authentication	We did not independently authenticate the cell lines used in this study.

Mycoplasma contamination	Cell lines were tested and confirmed negative for mycoplasma.
Commonly misidentified lines (See ICLAC register)	No commonly misidentified lines used.

Animals and other research organisms

Policy information about studies involving animals; ARRIVE guidelines recommended for reporting animal research, and Sex and Gender in Research

Laboratory animals	Isolated bone marrow for bone marrow derived macrophage differentiation from 6-20 week old C57Bl/6, Balb/c, mTmG, or FcRg KO mice bred in-house.<br>CD4 OT-II and CD8 OT-I cells were isolated from 6-20 week old OTI or OTII mice, male or female, bred in house.
Wild animals	No wild animals were used in this study.
Reporting on sex	All animal experiments used age and sex-matched animals. Both male and female mice were used for experiments with no reported differences in results based on sex.
Field-collected samples	No field-collected samples were used in this study.
Ethics oversight	Approved by UCSF IACUC.

Note that full information on the approval of the study protocol must also be provided in the manuscript.

Plants

Seed stocks	*Report on the source of all seed stocks or other plant material used. If applicable, state the seed stock centre and catalogue number. If plant specimens were collected from the field, describe the collection location, date and sampling procedures.*
Novel plant genotypes	*Describe the methods by which all novel plant genotypes were produced. This includes those generated by transgenic approaches, gene editing, chemical/radiation-based mutagenesis and hybridization. For transgenic lines, describe the transformation method, the number of independent lines analyzed and the generation upon which experiments were performed. For gene-edited lines, describe the editor used, the endogenous sequence targeted for editing, the targeting guide RNA sequence (if applicable) and how the editor was applied.*
Authentication	*Describe any authentication procedures for each seed stock used or novel genotype generated. Describe any experiments used to assess the effect of a mutation and, where applicable, how potential secondary effects (e.g. second site T-DNA insertions, mosiacism, off-target gene editing) were examined.*

Flow Cytometry

Plots

Confirm that:

☒ The axis labels state the marker and fluorochrome used (e.g. CD4-FITC).

☒ The axis scales are clearly visible. Include numbers along axes only for bottom left plot of group (a 'group' is an analysis of identical markers).

☒ All plots are contour plots with outliers or pseudocolor plots.

☒ A numerical value for number of cells or percentage (with statistics) is provided.

Methodology

Sample preparation	Sample preparation for both whole-cell and small particle flow cytometry from cell culture and tissues is described extensively in the methods section.
Instrument	BD Fortessa, BD FACSAria, BD FACSAria Fusion, or Cytek Aurora
Software	Acquisition of flow cytometry data using BD FACSDiva or Cytek SpectroFlo
Cell population abundance	Post-sort purity was determined by flow cytometry and confirmed to be 100% when sorting live, CD45+ macrophages for vesicle flow (see representative plots in Extended Data Figure 7) or greater than 95% when sorting live, CD11b+, ZsGreen+ macrophages for T cell stimulation assays (see representative plots in Extended Data Figure 9).
Gating strategy	For whole cell gating from tissues:<br>Single cells were gated according to their FSC/SSC profile. Live cells were gated according to negative signal in the live/dead channel.

Immune cells were gated based on positive CD45+ signal.
Lymphocytes were excluded as CD90.2+B220+NK1.1+ to focus analysis on myeloid cells.
Neutrophils: CD11b+Ly6G+
Alveolar macrophages: Ly6G+ CD11c+
Dendritic cells were distinguished by CD24+, MHCII+, CD11c+. Tissue resident dendritic cells were distinguished by CD103+ cDC1 and CD11b+ cDC2. For lymph nodes, migrating DCs were identified as MHCIIhiCD11cmid and resident DCs were identified as MHCIImidCD11chi.
Non-dendritic cells were distinguished by CD24-. Tissue-resident macrophages, including interstitial macrophages, were identified as CD11c+MHCII+. From non-macrophage populations, Ly6C+ conventional monocytes were identified in all tissues and and CD11c+ patrolling monocytes were identified in lung.

For gating of antigen transfer assays:
Single cells were gated according to their FSC/SSC profile. Live cells were gated according to negative signal in the live/dead channel.
Macrophages were gated on CD45 or CD11b positivity.
ZsGreen antigen positivity was gated on no-co-culture controls.

For gating of vesicle flow cytometry:
Single particles were gated according to their FSC/SSC profile.
Protein-associated vesicles were gated based on positive CellTraceViolet signal.
Intracellular vesicles were gated based on negative signal in the Strepaviding-BV650 channel.
ZsGreen antigen-containing vesicles were gated based on positive ZsGreen signal compared to no-co-culture controls.
Vesicle populations were identified by the following markers: EEA1+ early endosomes, RAB17+ recycling endosomes, RAB7+ maturing endosomes, LAMP1+ lysosomes, LAMP1+RAB7+CD63+ late endosomes.
MHC I and MHC II positivity were gated using FMO controls.

For gating of T cell assays:
Single cells were gated according to their FSC/SSC profile. Live cells were gated according to negative signal in the live/dead channel.
T cells were distinguished by CD90.2+CD11b- and subsequently gated on CD4+ or CD8+.
Proliferation was gated on loss of CellTraceViolet compared to no-stimulation controls.

☒ Tick this box to confirm that a figure exemplifying the gating strategy is provided in the Supplementary Information.
