# Supplementary material for: Submicrometre sampling of living cells by macrophages
Source: Nature. 2026 Apr 29;654(8118):495–503. doi: 10.1038/s41586-026-10435-5 (PMC13253326; doi:10.1038/s41586-026-10435-5)
Supplement: Supplementary file 4 — Peer Review File [file 41586_2026_10435_MOESM4_ESM.pdf]

nature portfolio

Peer Review File

Submicron-Sampling of Living Cells by Macrophages

Corresponding Author: Dr Matthew Krummel

Any redactions in this file are there to maintain patient confidentiality, the confidentiality of unpublished data, or to remove third-party material.

This file contains all reviewer reports in order by version, followed by all author rebuttals in order by version.

Version 1:

Reviewer comments:

Referee #1

(Remarks to the Author)
This study by Fan, Thota, Serwas et al. provides an elegant demonstration of a previously uncharacterised pathway of uptake of live cells fragments by myeloid cells, notably macrophages. The authors provide extensive descriptions of this transfer process using flow cytometry and advanced microscopy, showing that the transfer occurs through stretching of the target plasma membrane into the macrophage, before formation of sub-micron vesicles. They develop an assay to characterize intracellular vesicles by flow cytometry, distinguishing plasma membrane-derived from intracellular compartments and follow endosome maturation, which allows them to show that traffic of the trogocytosis-like particles to late endosomes/lysosomes is reduced compared to other uptake pathways. Finally, they propose that this trogocytosis-like pathway favours cross-presentation over MHC-II presentation, in agreement with reduced access to endosomal proteases.

Altogether, this manuscript describes a trogocytosis-like transfer pathway from tumor cells to macrophages. This is original and interesting. Nevertheless, the contribution of this pathway to the uptake of tumor or normal tissue, or to antigen cross presentation, in vivo were not analyzed (because of the lack tools to interfere specifically with the pathway). Nevertheless, in absence of this information, it is difficult to estimate the importance and relevance of this pathway in vivo.

Major points:
- In figure 1, the authors report the degree tumor-ZsGreen labelling in different types of immune cells. What about reverse? Do immune cell types also provide, if labelled with ZsGreen, transferred material to MO? In tumours most myeloid cells are ZsGreen+. Is this also the case for B and T lymphocytes? The authors should make clear that this in vivo transfer could be the result of dead cell phagocytosis or exosomes in tissues (as shown in multiple previous studies, including some cited in the manuscript).

- Figure 2 is critical to the message of the paper, but it is also not the most convincing. First, because there is no evidence that the in vitro assay mimics the in vivo transfer of observed in figure 1. In comparable in vitro assays, both exosomes and apoptotic bodies can be taken up by macrophages). Nevertheless, the in vitro assay used here provides evidence that live cells can transfer membrane materiel to MO. This evidence would be more convincing, by including the following controls:
o Include positive controls and titration for each drug. In panels e to g, the authors conclude that the transfer of ZsGreen does not occur through exosome, endocytosis or apoptosis, but they do not show that the drugs are actually affecting the corresponding functions (in these cells at the chosen concentrations).
o Cell death in drug-treated cells should also be closely monitored (to avoid toxicity issues).
In panel g, the authors use a GFP caspase 3 activity indicator, which is not transferred to MO under the used conditions (again a negative result). A positive control could be to use apoptotic tumor cells to show that the transfer is readily detected using this marker.

- The link to cross-reactive antibodies is very interesting, but the data provided are not sufficient to understand how opsonisation contributes to trogocytosis-like uptake in vivo. Could the authors analyse ZsGreen labelling in B-cell-deficient animals or in mice lacking Fc receptors on myeloid cells?

- The authors report that self Ags can effectively be taken up by macrophages from live cells. Are MHC-peptide complexes also captured by this pathway? (cross-dressing) The difference observed in OT-I vs OT-II activation described in the final

figure could result from the lack of MHC-II expression by tumor cells. Several strategies could be applied to test this: use MHC-I-deficient B16 OVA cells as targets, or MHC-II expressing ZsGreen+ cells as donors.

Minor points:
- Independent confirmation of the lack of lysosomal delivery after transfer should be obtained using, for example, conventional confocal microscopy. I absence of additional characterization of the "alternative compartment" the authors should not claim anything else than reduced lysosomal delivery.

- Between fig 2D (normalized uptake) and extended fig 2C (% ZsGreen BMDM), the picture looks quite different when analysing ZsGreen labelling from supernatant. Could the authors comment on their normalisation strategy? The claim that uptake does not come from extracellular vesicles is central in this paper and this point should be clarified.

Referee #2

(Remarks to the Author)
The manuscript by Fan et al. discribes a new antigen sampling mechanism, trogocytosis, by which live cells are sampled by in particular macrophages, to enhance crosspresentation to CD8 T cell responses, and not effecting any altered CD4 T cell response.
The paper uses high end microscopy and flow cytometry, cell labeling technique to show this in mouse model B16 OVA with BMDC in coculture systems with also read/outs of OTI and OTII.
The new antigen presentation route is interesting, relatively new, and shown, interestingly a predominant contribution of macrophages in this antigen sampling route and not dendritic cells. It therefore is novel and goes beyond our understanding of the dominating role of dendritic cells that are classically described as the best cross/presentating cells. The hypothesis is that this trogocytosis sampling route would mainly be involved in sampling auto/antigen for silencing responses, and homeostatic control
There are several questions still remaining that could be answered or improved;
1. BMDC, is a mix of cells more DC like and more macrophage like, do authors see that in the BMDC culture a subgroup of cells is involved in trogocytosis.
2. The authors show enhanced proliferation of CD8 T cells in figure 4, if this sampling would mainly play a role in homeostasis, where does this proliferation come from, how are these T cells activated.
3. What is exactly the proof that only cytoplasma is transfered and not organels
4. The authors talk about a new vesicle, are there any markers to identify these
5. in Figure 4 f, j and m it is not clear where in the quadrante the trogocytosis like up take is found, this should be better highlighted in the figures
6. It is not clear what MEF is in figure 4k
7. The trogocytosis could be identified with cellular co-cultures of murine cells, can this mechanism also be identified in human macrophages as co-culture, or identified in tissues as you need to have proper high intensity binding of macrophages and T cells.
8. The part of antibody opsonisation and integrin is rather vague and is confirmed only with extended data, would this mean that FcR are involved in creating and requirement to enhance this cell sampling. This part of receptors being involved like CR3 etc could be further extended and included in the figure within the paper not as extended. Furthermore this needs to be explained better how receptor mediated cell/cell contact results in the cytoplasma of one cell taken up by the macrophage.

Referee #3

(Remarks to the Author)
Fan et al start off with the in vivo observation that tumor and tissue associated antigens are often taken up by myeloid cells in small packages - different from the whole-cell engulfment often seen in efferocytic processes. The use several transgenic systems to convincingly make this point. They then set up an in vitro assay where the investiagate the sampling of BMDMs of target cell lines and replicate the phenomenon: the cells take up fragments of live cells that have not undergone apoptosis in a process similar to trgocytosis. They then set up a flow cytometry based assay to characterize through which compartments the material taken up via endocytosis / Phago(effero)cytosis / vs. trogocytosis traffics and find that these are processes that can be distinguished by the method. Finally they measure MHCII and MHC I presentation and find that the trogocytosed material is most efficiently cross-presented.

Although the phenomenology of this study is interesting it has to be said that Figs 1-3 are not very novel and not too surprising. Trogocytosis or nibbling has been described in myeloid cells for many decades and sophisticated studies showed transcellular flux of surface material (cross dressing) and its precise physiological consequences. The process Fan et all is interesting, as it is in between trogocytosis and phagocytosis and ultimately results in cross presentation of antigen. In this sense it is novel. However, there is no functional experiment that either investigates the process on a cell biological level (what determines this type of uptake on a molecular level and can it be manipulated by gain or loss of function experiments) or on a physiological level (what is the immunological consequence of antigens being cross-presented by this pathway, what happens if it is selectively eliminated). At least one of these two levels of questioning would have to be addressed to make a high profile paper.

Version 2:

Reviewer comments:

Referee #1

(Remarks to the Author)
The authors have addressed my comments and the revised manuscript is improved, Sebastian Amigorena

Referee #2

(Remarks to the Author)
The authors addressed all remarks and improved the paper considerably.
For me the only question that remains is that it seems that the receptors involved in the binding-uptake of life cells via trogocytosis such as CD93 are the same as phagocytosis for dead cells, where these receptors have been described intensively. If these receptors are the same for the initial interaction but consequently instruct differential routing remains difficult.
Also if this proces will happen simultanteously would the phagocytosis of dead cells hamper the trogo/like uptake of live cells that induces CD8 T cell proliferation?

Referee #3

(Remarks to the Author)
The authors added a substantial number of experiments that clarified many of the specific questions asked by Rev 1 and Rev 2. The most substantial addition was the SNX27Ko, that shifted the lysosomal trafficking pathway. However, my problem with the manuscript is still that it does neither describe a fundamentally new phenomenon, nor clarify a cell biological mechanism on a molecular level, nor assign a clear immunological function to the sampling mechanism. The authors acknowledge these unknowns in their discussion.
I find it especially unsatisfying that the authors now have a molecular target (SNX27) that they could employ to test if the sampling mechanism leads to tolerance or immunity, e.g. in a tumor setting.

Open Access This Peer Review File is licensed under a Creative Commons Attribution 4.0 International License, which permits use, sharing, adaptation, distribution and reproduction in any medium or format, as long as you give appropriate credit to the original author(s) and the source, provide a link to the Creative Commons license, and indicate if changes were made.
In cases where reviewers are anonymous, credit should be given to 'Anonymous Referee' and the source.
The images or other third party material in this Peer Review File are included in the article’s Creative Commons license, unless indicated otherwise in a credit line to the material. If material is not included in the article’s Creative Commons license and your intended use is not permitted by statutory regulation or exceeds the permitted use, you will need to obtain permission directly from the copyright holder.
To view a copy of this license, visit https://creativecommons.org/licenses/by/4.0/

We thank all three reviewers for their helpful and supportive reviews. We have strengthened many points as requested. As is often the case, there are a few directions that are suggested that are significantly orthogonal to one another. Within the confines of a single manuscript, we've largely focused on the 'mechanism' question related to the live-sampling derived compartment (see added manuscript main **Figure 5**). We've also added data on the in vivo emergence of this compartment in tumors, in addition to ~two dozen new experiments in duplicate (for in vivo experiments) or triplicate (for in vitro experiments) to build on our work, all of which are detailed below. In some cases, in the interest of keeping a coherent manuscript focused on this finding, we've suggested where some of this data may be 'reviewer only' but in most cases are happy to reconsider if editors and reviewers concur that these are essential for a final manuscript describing this biology. Thank you so much for your contributions to the work. All specific comments are addressed below and correspondingly in the revised text/figures.

Referee #1 (Remarks to the Author):

This study by Fan, Thota, Serwas et al. provides an elegant demonstration of a previously uncharacterised pathway of uptake of live cells fragments by myeloid cells, notably macrophages. The authors provide extensive descriptions of this transfer process using flow cytometry and advanced microscopy, showing that the transfer occurs through stretching of the target plasma membrane into the macrophage, before formation of sub-micron vesicles. They develop an assay to characterize intracellular vesicles by flow cytometry, distinguishing plasma membrane-derived from intracellular compartments and follow endosome maturation, which allows them to show that traffic of the trogocytosis-like particles to late endosomes/lysosomes is reduced compared to other uptake pathways. Finally, they propose that this trogocytosis-like pathway favours cross-presentation over MHC-II presentation, in agreement with reduced access to endosomal proteases.

Altogether, this manuscript describes a trogocytosis-like transfer pathway from tumor cells to macrophages. This is original and interesting. Nevertheless, the contribution of this pathway to the uptake of tumor or normal tissue, or to antigen cross presentation, in vivo were not analyzed (because of the lack tools to interfere specifically with the pathway). Nevertheless, in absence of this information, it is difficult to estimate the importance and relevance of this pathway in vivo.

Thank you for appreciating the originality and interest. We address your latter points in specific responses, below.

Major points:

- In figure 1, the authors report the degree tumor-ZsGreen labelling in different types of immune cells. What about reverse? Do immune cell types also provide, if labelled with ZsGreen, transferred material to MO? In tumours most myeloid cells are ZsGreen+. Is this also the case for B and T lymphocytes? The authors should make clear that this

in vivo transfer could be the result of dead cell phagocytosis or exosomes in tissues (as shown in multiple previous studies, including some cited in the manuscript).

To determine if ZsGreen$^+$ immune cells can transfer material to macrophages, we co-cultured CD45. 1 BMDMs with ZsGreen$^+$ splenocytes isolated from Vav-iCre;Ai6 mice. In these highly viable cultures, we observed an increase in ZsGreen signal in BMDMs, indicating that ZsGreen can be transferred from immune cells to macrophages (**Reviewer Fig. 1a, b**).

Next, we evaluated if lymphocytes acquire ZsGreen in vivo in our B16-ZsGreen tumor model. While we detected low-level ZsGreen uptake by some lymphocyte populations, the majority remain ZsGreen- and the ZsGreen signal was substantially lower than that of tumor-associated myeloid cells (**Reviewer Fig. 1c, d**).

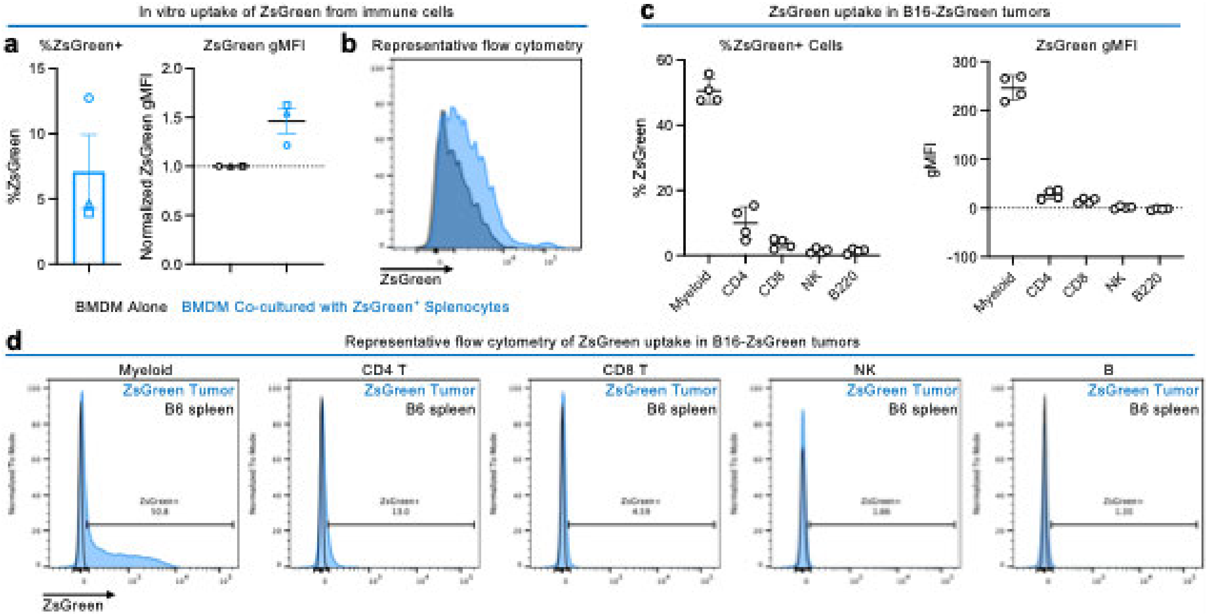

Reviewer Fig. 1: ZsGreen uptake from immune cells or by lymphocytes.
a-b, CD45.1 BMDMs were co-cultured with ZsGreen$^+$ splenocytes isolated from CD45.2 Vav-iCre;Ai6 mice for 2 hours in D10 supplemented with 10ug/mL αIgM Fab, 20 ng/mL BAFF, 10 ng/mL IL-7, 10 ng/mL IL-15, 20 ng/mL GM-CSF, 10 ng/mL IL-3, and 20 nM zVAD and subsequently evaluated for ZsGreen positivity by flow cytometry. **a**, Left, quantification of ZsGreen$^+$ BMDMs in co-culture; right, ZsGreen gMFI normalized to BMDM cultured alone. n = 3 mice. Shown are mean +/- s.e.m. **b,** Representative flow cytometry histogram of BMDMs cultured alone (black) or with ZsGreen$^+$ splenocytes (blue). **c-d,** Myeloid cells, CD4$^+$ T cells (CD4), CD8$^+$ T cells (CD8), natural killer (NK) cells, and B cells were evaluated for ZsGreen positivity in B16-ZsGreen tumors. **c**, n = 4 mice representative of 2 experiments. Shown are mean +/- s.d. **d**, Representative flow cytometry histogram of immune cells isolated from ZsGreen tumors or control B6 spleens.

Finally, to make explicit that in vivo transfer can also result from dead cell phagocytosis or exosomes, we have modified our text (indicated in blue, **Lines 104-105**):
Several mechanisms of uptake might contribute to myeloid cell sampling from tissues in vivo, with the most heavily studied to date being phagocytosis of dead cells and endocytosis of exosomes.

- Figure 2 is critical to the message of the paper, but it is also not the most convincing. First, because there is no evidence that the in vitro assay mimics the in vivo transfer of observed in figure 1. In comparable in vitro assays, both exosomes and apoptotic bodies can be taken up by macrophages). Nevertheless, the in vitro assay used here provides evidence that live cells can transfer membrane materiel to MO. This evidence would be more convincing, by including the following controls:
    - Include positive controls and titration for each drug. In panels e to g, the authors conclude that the transfer of ZsGreen does not occur through exosome, endocytosis or apoptosis, but they do not show that the drugs are actually affecting the corresponding functions (in these cells at the chosen concentrations).
    - Cell death in drug-treated cells should also be closely monitored (to avoid toxicity issues).
    - In panel g, the authors use a GFP caspase 3 activity indicator, which is not transferred to MO under the used conditions (again a negative result). A positive control could be to use apoptotic tumor cells to show that the transfer is readily detected using this marker.

We appreciate the reviewer's thoughtful comments and we thank this reviewer for recommending controls to strengthen the evidence for live-cell material transfer in the isolated cell (in vitro) situation of Figure 2. This assay studies live-sampling in a tractable system and allows us to ask specific questions, to which the reviewer has rightly made some excellent suggestions. In response, we have performed and incorporated the following additions:

First, we now include titration curves examining the toxicity and the activity of exosome inhibitors (DMA, calpeptin), endocytosis inhibitor (Dynasore, Dynole 34-2), and caspase inhibitor (zVAD) (**Reviewer Fig. 2a-f**). In doing so, we found that calpeptin affected B16 exosome production much less than DMA and barely significantly and dynasore treatment did not significantly reduce uptake of B16-derived exosomes (data not shown) and Dynole (see below) was much more effective. As such, we have removed these data from our manuscript and focused on the effect of treatment with DMA and Dynole 34-2.

More to the reviewer's point, we identified non-toxic concentrations for inhibitors of these processes, validated their function for the supplemental figures, and then included in the main **Fig. 2** the study of live-sampling assay in the context of the non-lethal/effective dose. Specifically:

- To target cell exosome release, we found 10uM DMA is non-toxic and significantly decreased ZsGreen extracellular vesicle production (**Reviewer Fig. 2a, d**).
- To target BMDM endocytosis of such endosomes, we identified that 5uM Dynole 34-2, an alternative and irreversible dynamin inhibitor, was non-toxic and ablated uptake of ZsGreen exosomes (**Reviewer Fig. 2b, e**)
- For assessing modifiers of target cell apoptosis, we validated that 20uM and 30uM zVAD are effective for B16-ZsGreen and MEF-ZsGreen target cells (**Reviewer Fig. c, f**).

We then repeated our antigen transfer assays for main figure with 5uM Dynole 34-2 (**Reviewer Fig. 2g, h**) and MEF-ZsGreen co-cultures assays using 30uM zVAD (**Reviewer Fig. 2h**). Neither inhibiting endocytosis nor caspase reduced ZsGreen uptake. Together, these data support our original results (done with slightly different concentrations and a different dynamin inhibitor) that exosomes and cell death are not the predominant contributors ZsGreen sample in our system (and indeed that dynamin function is not essential for live-sampling).

To provide further assurances that inhibitors were used at non-toxic concentrations in the experiments reported in the manuscript, we quantified total BMDM, B16-ZsGreen target cell, and MEF-ZsGreen target cell numbers and found that cell number was not significantly reduced (**Reviewer Fig. 2f**).

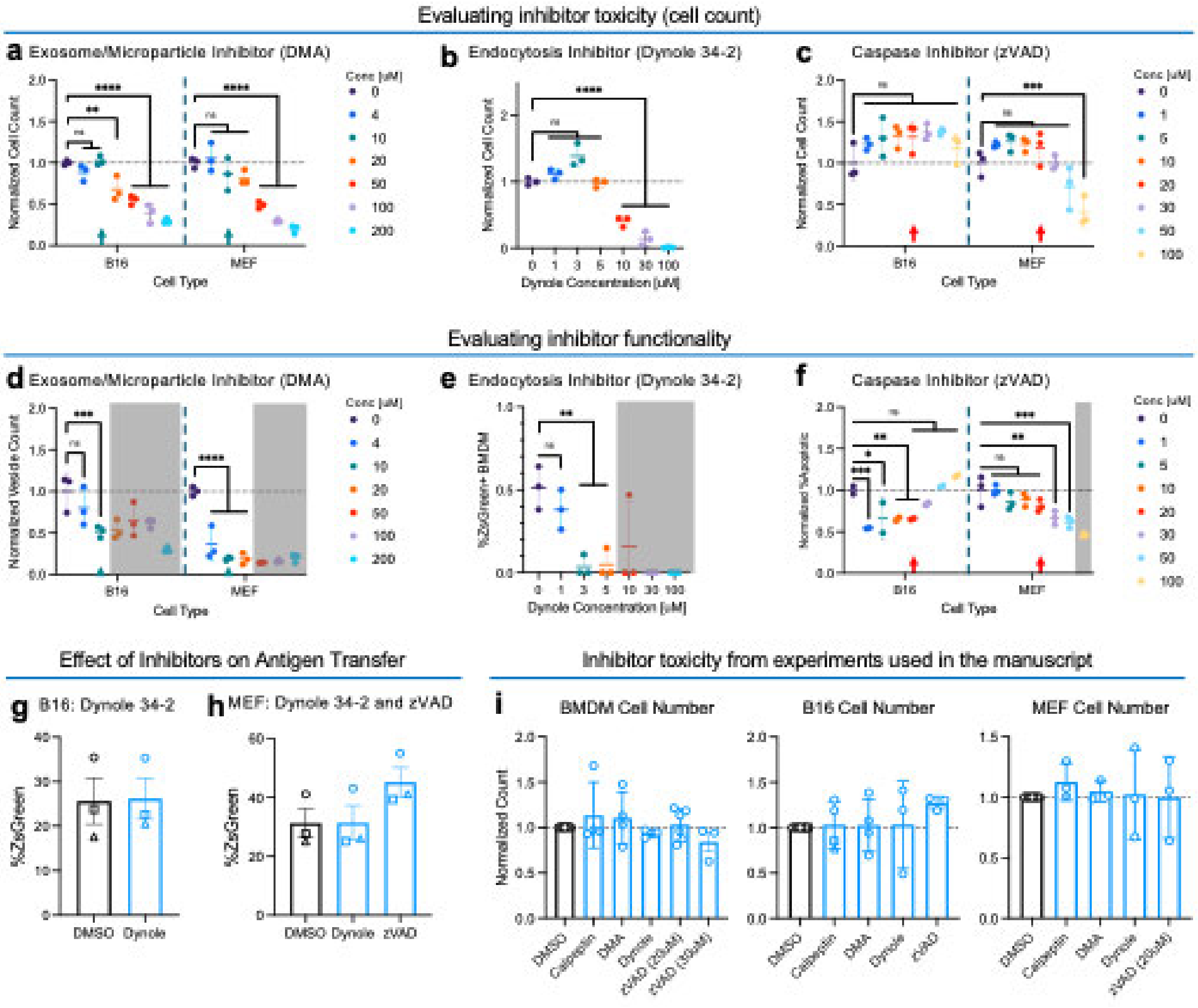

Reviewer Fig. 2: Drug titrations against cell viability and inhibitor function to validate conclusions in main figure concerning role of various pathways.
a-c, Indicated cell types were cultured with the exosome/microparticle release inhibitor DMA (**a**), irreversible dynamin inhibitor Dynole 34-2 (**b**), caspase inhibitor zVAD (**c**). Cell number was evaluated by flow cytometry using CountBright beads to monitor toxicity.

d, B16-ZsGreen or MEF-ZsGreen target cells were cultured with the exosome/microparticle release inhibitor DMA supernatant was collected to quantify ZsGreen+ vesicles by small particle flow cytometry. Teal arrows indicate concentration used in this study (10 uM DMA) and toxic concentrations are grayed out for functional studies in main figures. Shown are n = 3 technical replicates, mean +/- s.d.
e, BMDMs were cultured with ZsGreen exosomes isolated using the ExoQuick kit with indicated concentrations of irreversible dynamin inhibitor Dynole 34-2. Exosome endocytosis was measured by flow cytometry by quantifying %ZsGreen+. Toxic concentrations are grayed out for functional studies. Shown are n = 3 technical replicates, mean +/- s.d.
f, B16-ZsGreen or MEF-ZsGreen target cells were cultured with indicated concentrations of caspase inhibitor zVAD in the presence of Staurosporine. %AnnexinV^{+}DAPI$^{+/-}$ apoptotic cells were evaluated by flow cytometry. Red arrows indicate concentration used in this study (20uM zVAD) and toxic concentrations are grayed out for functional studies. Shown are n = 3 technical replicates, mean +/- s.d.
g, B16-ZsGreen antigen transfer assay in the presence of DMSO or 5uM Dynole 34-2. Shown are n = 3 biological replicates, mean +/- s.e.m. **h,** MEF-ZsGreen antigen transfer assay in the presence of DMSO, 5uM Dynole 34-2, or 30 uM zVAD. Shown are n = 3 biological replicates, mean +/- s.e.m. **i,** Total BMDM, B16, and MEF numbers in the assays reported in this manuscript or in the revision experiments in (**d, e**). n = 3-6 biological or experimental replicates. Shown are mean +/- s.e.m.

Finally, we now highlight in the text the control that we had performed wherein we can detect GFP in macrophages that have been co-cultured with Staurosporine-treated apoptotic tumor cells expressing the GFP caspase 3 indicator, showing that a distinct population of GFP^{+} macrophages would have been detectable in the live-sampling assay had they sampled from cells undergoing caspase activation. We reproduce the text (**Lines 171-174**) and extended data (**Extended Data Fig. 2h**) for the reviewer below:

> When these reporter-expressing cells were co-cultured with BMDM, we found that the majority of the mCherry-positive cells (those that had taken up material from the donor cells) lacked GFP expression compared to uptake following apoptosis (1% for live sampling**,** versus 11% following staurosporine; **Fig. 2j, Extended Data Fig. 2h**).

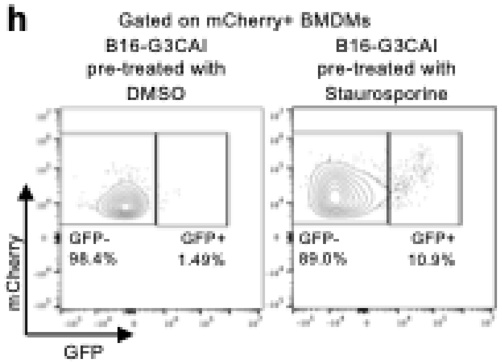

Extended Data Fig. 2h: BMDM co-culture with B16-F10 expressing GFP caspase 3 activity indicator (B16-GC3AI) demonstrates uptake is predominantly from live cells.

Together with the data in **Fig. 2** and **Extended Data 2**, these additional experiments strengthen the overall conclusion that live-cell, contact-dependent sampling contributes substantially to the transfer of material to macrophages in our in vitro system. We have incorporated these data as revised **Fig. 2g**, **Extended Data 2c-g,** and **Supplementary Fig. 4.**

- The link to cross-reactive antibodies is very interesting, but the data provided are not sufficient to understand how opsonisation contributes to trogocytosis-like uptake in vivo. Could the authors analyse ZsGreen labelling in B-cell-deficient animals or in mice lacking Fc receptors on myeloid cells?

  Thank you for this comment. We preface our reply in noting that while we can show that nibbling of live-cells is boosted by antibodies/FcR engagement, our data shows that this just one of what is likely a constellation of receptor-ligand pairs that can facilitate the process (see also our data also on additional receptors, that also contribute).

  To the point of antibody contributions, we agree that it is important to determine how opsonization may contribute to uptake in vivo. To test this, we evaluated ZsGreen labeling in B16-ZsGreen tumors in muMT mice, which lack mature B cells and circulating antibodies. We found reduced ZsGreen uptake in tumor-associated myeloid cells in muMT mice compared to wild-type B6 mice. Specifically, the proportion of ZsGreen^{+} monocytes and cDC2s was decreased, and the amount of ZsGreen uptake (by gMFI) was reduced in monocytes, macrophages, and cDC2s. These findings suggest that antibody-mediated opsonization facilitates uptake of tumor-derived material by myeloid cells in vivo. We were unsure whether include these data in our revised manuscript and ultimately elected not to as ZsGreen signal in vivo can be derived from multiple sampling methods, although, these data match our in vitro results (see **Fig. 3e-f**, **Extended Data Fig. 4**).

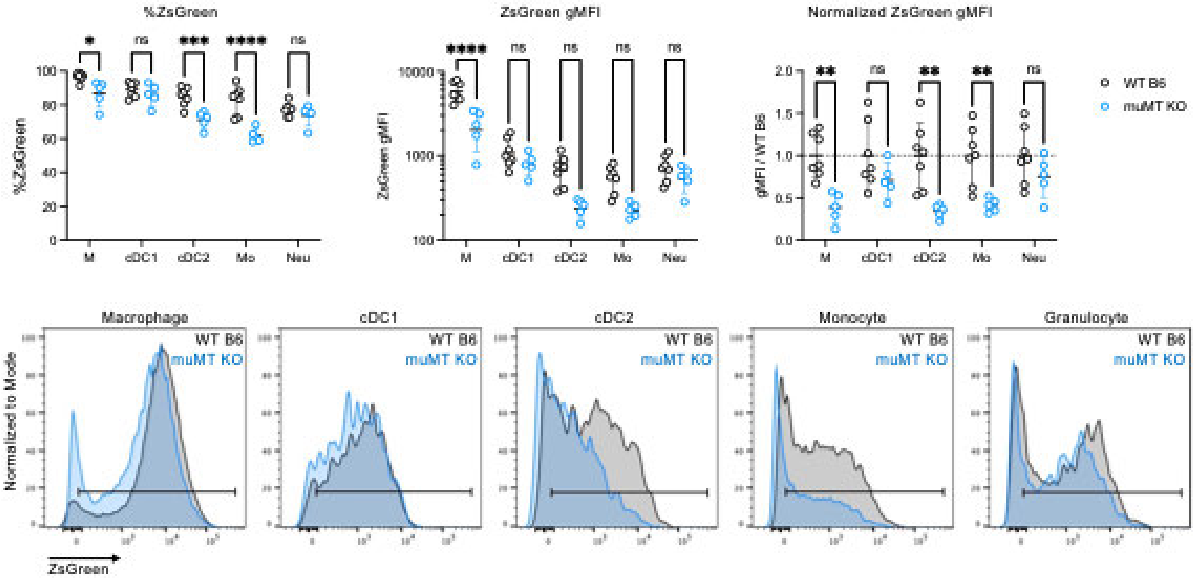

Reviewer Fig. 3: muMT KO mice lacking mature B cells display decreased ZsGreen labeling of tumor associated myeloid cells in B16-ZsGreen tumors in mice
B16-ZsGreen tumor cells were subcutaneously transplanted into wild-type B6 (WT B6, black) or muMT knockout (muMT KO, blue) and tumor-associated myeloid cells were isolated to evaluate ZsGreen labeling. cDC1, conventional dendritic cell 1; cDC2, conventional dendritic cell 2. Shown are means +/- s.d. n = 5-7 mice per condition, representative of 2 experiments. Two-way ANOVA with Sidak's multiple comparison. ns not significant, * $p < 0.05$, *** $p < 0.001$, **** $p < 0.0001$.

- The authors report that self Ags can effectively be taken up by macrophages from live cells. Are MHC-peptide complexes also captured by this pathway? (cross-dressing) The difference observed in OT-I vs OT-II activation described in the final figure could result from the lack of MHC-II expression by tumor cells. Several strategies could be applied to test this: use MHC-I-deficient B16 OVA cells as targets, or MHC-II expressing ZsGreen+ cells as donors.

  We appreciate the reviewer's insightful comment regarding the possibility that MHC-peptide complexes from the target cell can be transferred onto the macrophage surface and contribute to T cell activation. This has been suggested in other settings but it is not clear whether it happens in the cell biology system that we've carefully set up and validated in Figure 2 and which now focuses on live-sampling as a solid mechanism for macrophages.

  As the reviewer suggested, we tested whether this process depends on target cell MHC-I expression by using MHC-I-deficient target cells. To this point, we show that the B6-derived B16-ZsGreen-minOVA line used in our T cell experiments lacks detectable levels of surface MHC-I expression in vitro (**Reviewer Fig. 4b**) suggesting immediately that none of what we are measuring would be such cross-dressing.

  Nonetheless, there could be small quantities still present, and so, to evaluate if peptide-MHC is transferred onto the macrophage surface, we performed antigen transfer and T cell stimulation assays using Balb/c-derived BMDMs that likewise lack the H2-Kb alleles (**Reviewer Fig. 4a**). Staining revealed that MHC-I allele H2Kb was not detected on either Balb/c BMDMs cultured alone or Balb/c BMDMs that had ingested ZsGreen from B16-ZsGreen-minOVA (**Reviewer Fig. 4b**). Further, Balb/c BMDMs that had taken up ZsGreen were unable to upregulate CD69 on T cells and did not drive substantial T cell proliferation (**Reviewer Fig. 4c**). Thus, while limited peptide-MHC transfer may occur in our setting (and certainly appears to in some in vivo assays done by others), the majority of CD8 T cell activation must be due to BMDM processing of ingested cytosolic antigen. We have included these data in **Extended Data Fig. 9** in our revised manuscript.

  The reviewer makes an independent point about tumors lacking Class II. Given that MHC-I is not transferred, we consider it unlikely that lack of MHC-II on the tumor contributes to the difference in OT-I and OT-II activation. In any case, we found CD4 T cells to be poorly stimulated by our macrophages (**Fig. 4o**), likely a result of their low overall Class II expression, and our data points to live-sampling as profoundly feeding class I presentation.

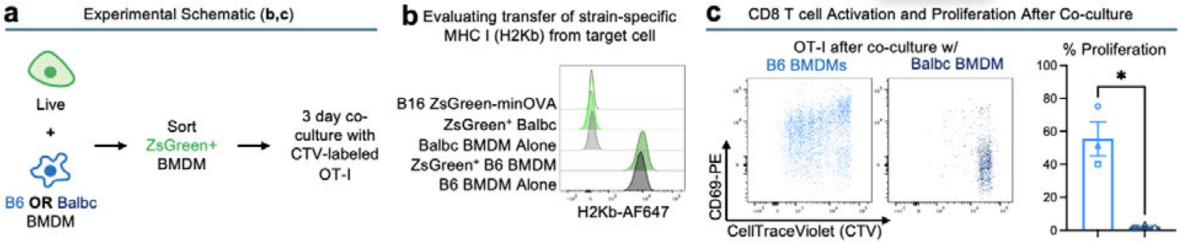

Reviewer Fig. 4: Peptide-MHC transfer does not account for the majority of T cell activation by macrophages that have sampled cytosolic antigen using a trogocytosis-like mechanism
a, Experimental schematic to evaluate strain specific MHC-I transfer and T cell activation by ZsGreen$^+$ B6 or Balbc-derived BMDMs. **b**, H2-Kb expression on B16 ZsGreen-minOVA target cells; ZsGreen+ BMDMs after co-culture with B16 ZsGreen-minOVA cels, and BMDMs cultured alone. **c**, OT-I CD8 T cell proliferation after 3-day co-culture with FACS-isolated ZsGreen+ B6 or Balb/c BMDMs. n = 3 experiments. Shown are mean of technical replicates +/- s.e.m. Paired t-test.

Minor points:

- Independent confirmation of the lack of lysosomal delivery after transfer should be obtained using, for example, conventional confocal microscopy. I absence of additional characterization of the “alternative compartment” the authors should not claim anything else than reduced lysosomal delivery.

  We appreciate the reviewers’ suggestion to provide additional validation of reduced delivery to the lysosome. In response, we have performed confocal microscopy and quantitative co-localization analysis, which confirm that ZsGreen antigen acquired through live-cell sampling have decreased overlap with LAMP1 compared to antigen acquired from phagocytosis (**Reviewer Fig. 5a, b**). These results are consistent with our vesicle flow analysis and included in our revised manuscript as **Extended Data Fig. 5**.

  To further characterize this compartment, we evaluated additional markers and trafficking regulators for which flow-functional antibodies were available. ZsGreen$^+$ vesicles from live-sampling are largely negative for RAB11A and SEC22B (**Reviewer Fig. 5c**), which have previously been suggested to mark cross-presentation compartments in dendritic cells (PMID 36804685). The fact that these do not overlap may be taken as a result that the ‘alternative compartment’ that we describe is not actively involved in presenting (i.e. more of a holding compartment) or that these previous studies focused on a form of cross-presentation in response to phagocytic material that led to antigens being presented on Class I through these mechanisms in a way that is not the same as what happens with live-sampling by macrophages.

  An important finding that was made during our review period is that knockout of the “sorting nexin” SNX27 increases trafficking of live-sampled ZsGreen+ material to LAMP1$^+$ lysosomes. We believe that this relief from lysosomal fusion further emphasizes that what we refer to as an ‘alternative compartment’ is indeed protected from lysosomal delivery, or to the reviewer’s focus, is in fact normally not effectively delivered to LAMP1$^+$ vesicles (**Reviewer Fig. 5d-h**)

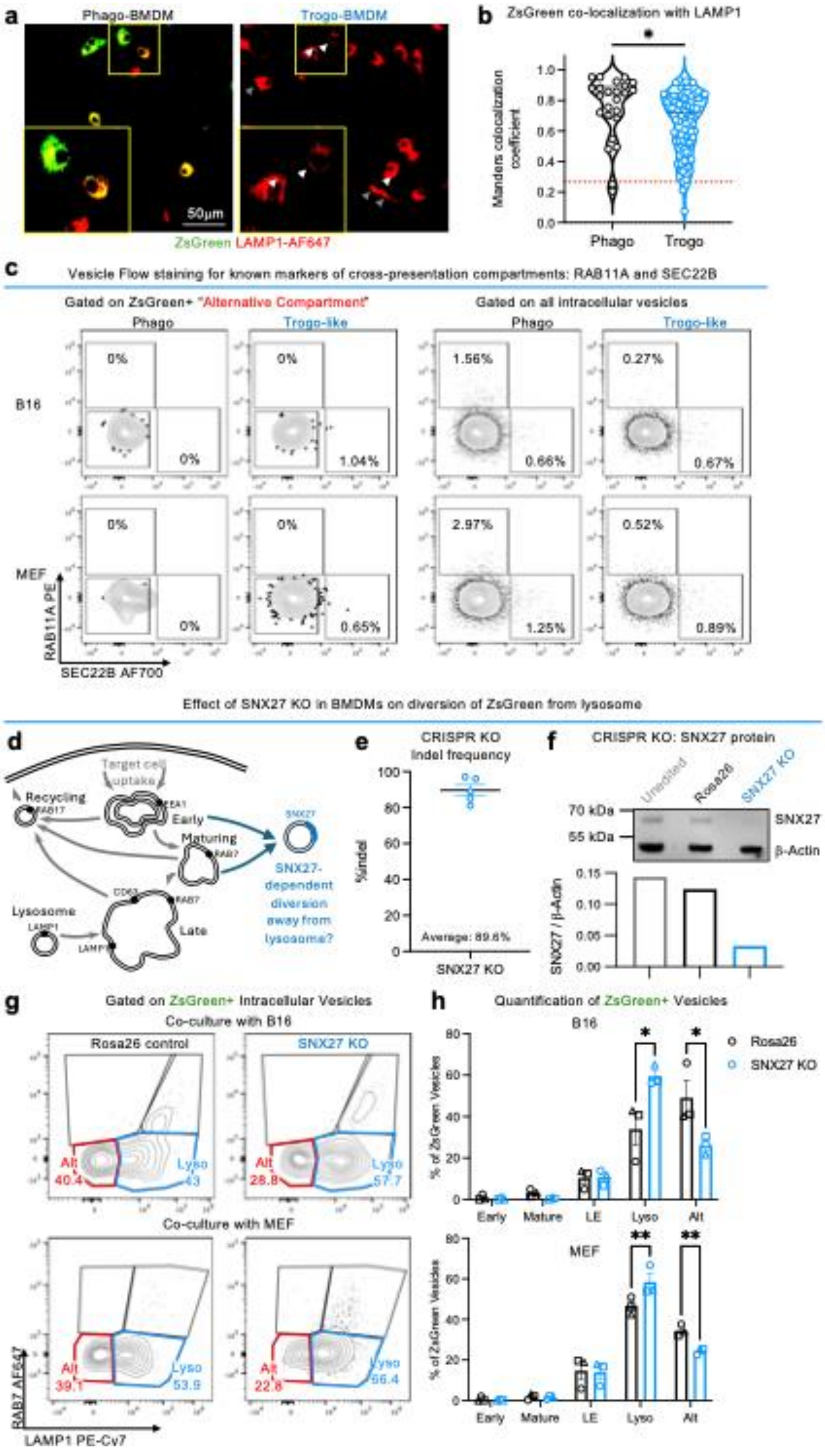
a
Phago-BMDM
Trogo-BMDM
50µm
ZsGreen LAMP1-AF647
b
ZsGreen co-localization with LAMP1
Manders colocalization coefficient
Phago
Trogo
c
Vesicle Flow staining for known markers of cross-presentation compartments: RAB11A and SEC22B
Gated on ZsGreen+ "Alternative Compartment"
Gated on all intracellular vesicles
Phago
Trogo-like
B16
MEF
RAB11A PE
SEC22B AF700
Effect of SNX27 KO in BMDMs on diversion of ZsGreen from lysosome
d
Target cell uptake
Recycling
Early
Maturing
Lysosome
Late
SNX27-dependent diversion away from lysosome?
e
CRISPR KO Indel frequency
%indel
Average: 89.6%
SNX27 KO
f
CRISPR KO: SNX27 protein
Unedited
Rosa26
SNX27 KO
70 kDa
55 kDa
SNX27
β-Actin
SNX27 / β-Actin
g
Gated on ZsGreen+ Intracellular Vesicles
Co-culture with B16
Rosa26 control
SNX27 KO
Co-culture with MEF
RAB7 AF647
LAMP1 PE-Cy7
h
Quantification of ZsGreen+ Vesicles
B16
MEF
% of ZsGreen Vesicles
Early
Mature
LE
Lyso
Alt
Rosa26
SNX27 KO

Reviewer Figure 5: Validation and characterization of ZsGreen antigen trafficking downstream of live-cell sampling

a-b Sorted ZsGreen+ BMDMs after co-culture with killed B16-ZsGreen (Phago-BMDM) and live B16-ZsGreen (Trogo-BMDM) were fix, permeabilized, and stained with LAMP1-AF647. Representative images are shown in (**a**) and quantification per field of view is shown in (**b**). Phago, n = 24; Trogo, n=96 student's t-test. **c**, Vesicle flow analysis of ZsGreen$^+$ vesicles and all intracellular vesicles for RAB11A and SEC22B. **d,** Schematic illustrating SNX27-dependent diversion of material away from lysosomal maturation into alternative endocytic pathways. **e-f** Validation of SNX27 KO in BMDMs by (**e**) indel quantification using ICE analysis (mean +/- s.d.) and (**f**) western blot (representative of 2 experiments). **g-h**, CRISPR-Cas9-targeted BMDMs were co-cultured with ZsGreen target cells to evaluate ZsGreen trafficking. Shown are mean +/- s.e.m. n = 3 biological replicates. Two-way ANOVA with Sidak's multiple comparison. *, $p < 0.05$, ** $p < 0.01$.

Together, these results strengthen our conclusion that antigen trafficking downstream of live-sampling is distinct from canonical endocytic degradation pathways and known cross-presentation compartments. We acknowledge that the precise and unique molecular markers for this compartment remains to be defined and obviously this will be subject of much future work (ongoing). Also, while we maintain the usage of "alternative compartment" to refer to vesicles not labeled by the markers we have included in our vesicle flow panel, and we have modified our language in the discussion to focus on antigen trafficking (line **448-451**), highlighted in blue below:

> A key distinction here is in revealing that a very low-volume trogocytosis-like sampling may be ongoing at steady state in a variety of cells and may be a significant source of self-antigen from healthy cells, giving macrophages a distinct endocytic trafficking mechanism with which to present healthy-self antigens, notably to CD8 T cells.

- Between fig 2D (normalized uptake) and extended fig 2C (% ZsGreen BMDM), the picture looks quite different when analysing ZsGreen labelling from supernatant. Could the authors comment on their normalisation strategy? The claim that uptake does not come from extracellular vesicles is central in this paper and this point should be clarified.

We thank the reviewer for raising this important point regarding our normalization strategy and its impact on data presentation. To calculate normalized ZsGreen uptake, we first averaged the %ZsGreen$^+$ for the technical replicates for DMSO controls within each biological replicate and then divided each experimental value by this control average. We reported the mean normalized value for each biological replicate. In other words, uptake is effectively a fold-change between the control and drug-treatment conditions.

In **Fig. 2d** (now **Fig. 2e**), we show the normalized values for uptake in direct co-culture or transwell conditions with B16-ZsGreen target cells. In former **Extended Data Fig. 2c** (now **Extended Data Fig. 2b**), we show the unnormalized values for uptake under the same conditions with MEF-ZsGreen target cells. To aid in comparison, we include below the corresponding unnormalized B16-ZsGreen target cells. In these unnormalized data, transwell co-culture decreases uptake from B16-ZsGreen target cells, supporting our conclusion that uptake from extracellular vesicles is not the dominant contributor to antigen transfer in our system.

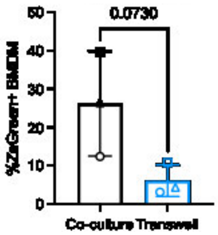

Reviewer Fig. 6: Unnormalized B16-ZsGreen supernatant uptake by BMDMs, related to Fig. 2e

Referee #2 (Remarks to the Author):

The manuscript by Fan et al. discribes a new antigen sampling mechanism, trogocytosis, by which live cells are sampled by in particular macrophages, to enhance crosspresentation to CD8 T cell responses, and not effecting any altered CD4 T cell response.

The paper uses high end microscopy and flow cytometry, cell labeling technique to show this in mouse model B16 OVA with BMDC in coculture systems with also read/outs of OTI and OTII.

The new antigen presentation route is interesting, relatively new, and shown, interestingly a predominant contribution of macrophages in this antigen sampling route and not dendritic cells. It therefore is novel and goes beyond our understanding of the dominating role of dendritic cells that are classically described as the best cross/presentating cells. The hypothesis is that this trogocytosis sampling route would mainly be involved in sampling auto/antigen for silencing responses, and homeostatic control

Thank you for these comments that highlight the novelty and importance of this work.

There are several questions still remaining that could be answered or improved;

1. BMDC, is a mix of cells more DC like and more macrophage like, do authors see that in the BMDC culture a subgroup of cells is involved in trogocytosis.

   In our study, we use BMDM (macrophage cultures) and not BMDC but understand that this reviewer is interested in the possibility that different myeloid cells do live-sampling differently, perhaps motivated by the observation that macrophages typically do this more in the non-inflamed steady-state (i.e everything except tumor in **Fig.** 1 and **Extended Data Fig. 1**).

   Thus, while we primarily used M-CSF to generate bone marrow derived macrophages in our study, we recognize that GM-CSF bone marrow cultures in particular yield a heterogenous population of dendritic cell (DC) and macrophage subsets (PMID: 26084029). In our GM-CSF-derived cultures, we identified a MHC-II^{-} macrophage population in addition to the 4 populations described by Helft et al (PMID: 26084029): CD11c-MHC-, MHCII+ macrophages, dendritic cells (GM-DCs), and Csf1r-Flt3- cells (GM-double negative, GM-DN) (**Reviewer Fig. 7a**).

[REDACTED]

[REDACTED]

2. The authors show enhanced proliferation of CD8 T cells in figure 4, if this sampling would mainly play a role in homeostasis, where does this proliferation come from, how are these T cells activated.

   We appreciate the opportunity to explore the homeostatic relevance of our findings. Because the model antigens that we use in the manuscript (i.e. ovalbumin) are not selected for during T cell development, transgenic OT-I CD8 T cells retain high-affinity TCRs that response robustly to even low levels of antigen presentation. In vivo, high-affinity self-antigen specific T cells are typically eliminated in the thymus. So, we use these transgenic T cells to reveal the enhanced cross-presentation downstream of live sampling of antigen rather than as a direct model of physiological homeostatic T cell maintenance and tuning. To clarify this point, we added the following statement into our discussion section (**lines 450-453**):
   While we use model antigen and transgenic T cells in our study to reveal the capability of macrophages to cross-present live-cell-associated antigen by stimulating T proliferation, the functional role of this cross-presentation for maintaining or tuning homeostatic T cell populations that have undergone thymic self-tolerance requires further investigation.

3. What is exactly the proof that only cytoplasma is transfered and not organels

   While we focus on transfer of cytosolic ZsGreen and model antigens in our study, we do not claim that only cytoplasm is transferred. To formally evaluate whether other

[REDACTED]

[REDACTED] [redacted]

4. The authors talk about a new vesicle, are there any markers to identify these

   We thank the reviewer for this insightful question regarding molecular markers that define the newly described compartment. Our revision experiments indicate that this “alternative” compartment is distinct from known cross-presentation compartments such as RAB11A and SEC22B. Specifically, we find that the ZsGreen$^+$ vesicles in the “alternative compartment” are largely negative for the cross-presentation markers described in dendritic cells, RAB11A and SEC22B (PMID 36804685), although vesicles positive for these markers are present when gating on all intracellular vesicles (**Reviewer Fig. 9a**). The fact that these do not overlap may be taken as a result that either the ‘alternative compartment’ that we describe is not actively involved in presenting (i.e. more of a holding compartment) or that these previous studies focused on a form of cross-presentation in response to phagocytic material that led to antigens being presented on Class I in a way that distinct from live-sampling.

   Furthermore, perturbation of the sorting nexin SNX27 increases antigen trafficking to LAMP1$^+$ lysosomes, suggesting that this compartment normally avoids lysosomal fusion through SNX27-dependent trafficking. (**Reviewer Fig. 9b-f**)

   These results suggest that the alternative compartment is distinct from classical phagosomal and endosomal pathways, though additional molecular characterized will be required to define unique markers for this structure.

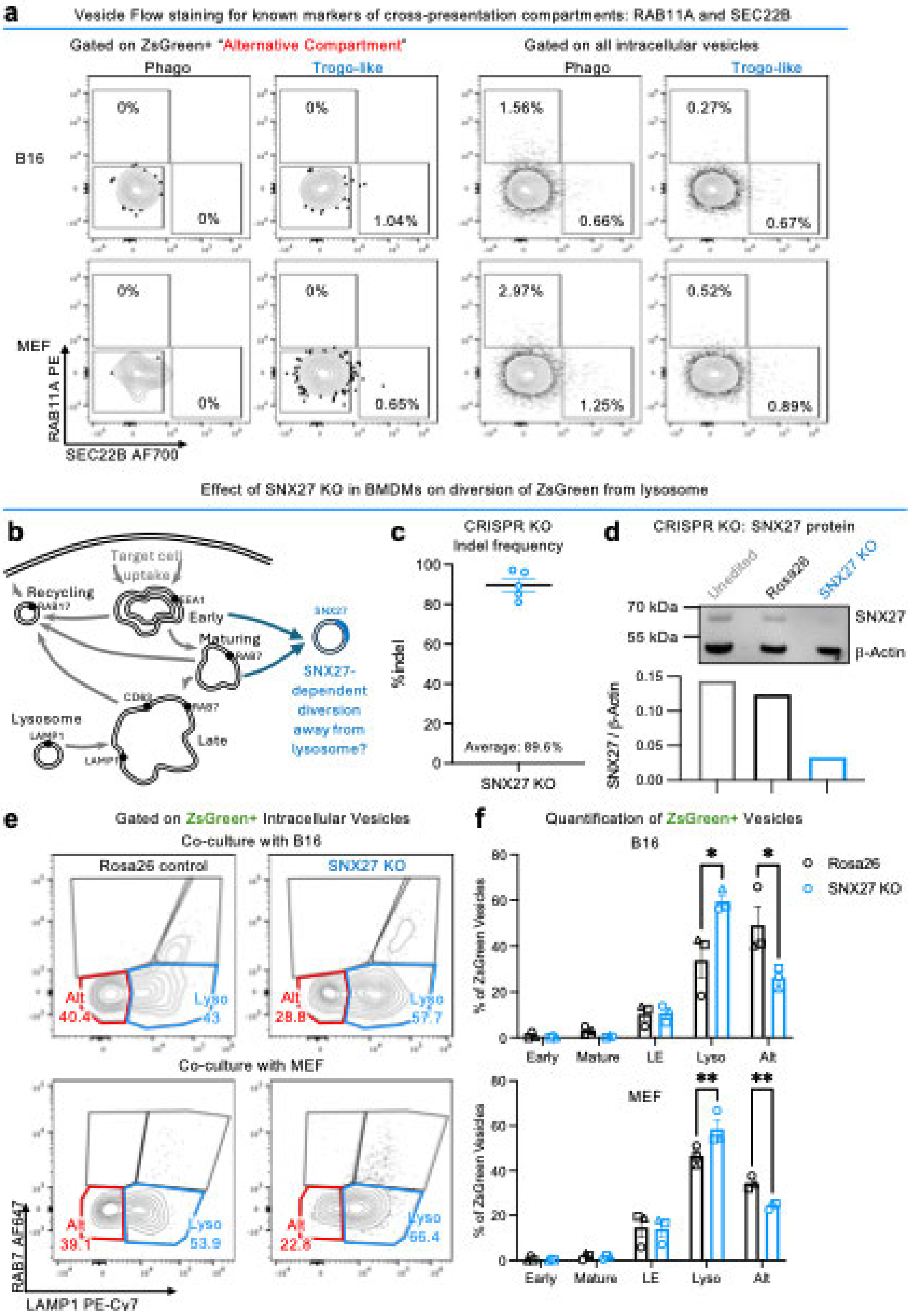

Reviewer Figure 9: Characterization of marker expression and trafficking properties of the alternative compartment

a, Vesicle flow analysis of ZsGreen+ vesicles and all intracellular vesicles for RAB11A and SEC22B. **b**, Schematic illustrating SNX27-dependent diversion of material away from lysosomal maturation into alternative endocytic pathways. **c-d**, Validation of SNX27 KO in BMDMs by (**c**) indel quantification using ICE analysis (mean +/- s.d.) and (**d**) western blot (representative of 2 experiments). **e-f**, CRISPR-Cas9-targeted BMDMs were co-cultured with ZsGreen target cells to evaluate ZsGreen trafficking. Shown are mean +/- s.e.m. n = 3-6 experiments. Two-way ANOVA with Sidak's multiple comparison. *, $p < 0.05$, ** $p < 0.01$.

5. in Figure 4 f, j and m it is not clear where in the quadrante the trogocytosis like up take is found, this should be better highlighted in the figures

   We thank the reviewer for this helpful suggestion to clarify the localization of this vesicle population. While **Figure 4f** outlines the vesicle gating scheme independent of uptake mechanism, we have now highlighted the "alternative" compartment that is enriched downstream of trogocytosis-like sampling in **Figures 4j** and **4m** of the revised manuscript (reproduced below as **Reviewer Fig. 10**).

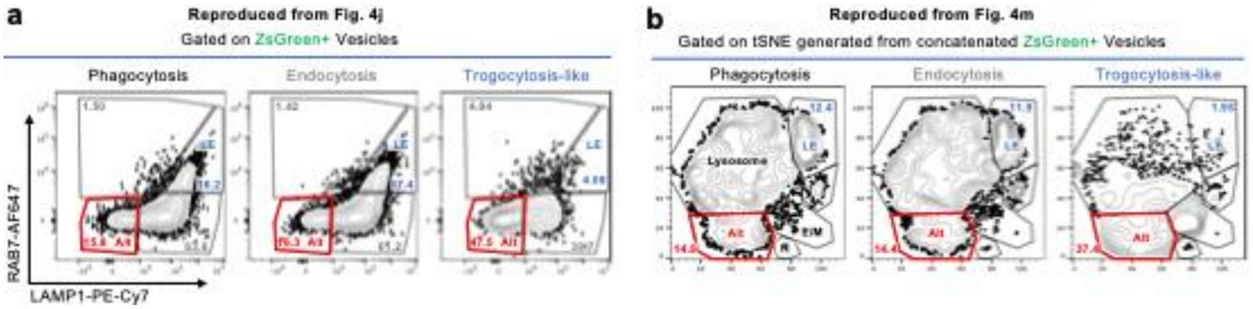

Reviewer Fig. 10: ZsGreen antigen is enriched in a marker-negative alternative compartment (red) downstream of trogocytosis-like sampling.
a-b, Representative flow plots (**a**) or tSNE (**b**) of ZsGreen+ vesicles derived from different sampling mechanism with alternative compartment enriched downstream of trogocytosis-like sampling highlighted in red. LE, late endosome; Alt, alternative compartment; E/M, early/maturing endosomes; R, recycling endosomes.

6. It is not clear what MEF is in figure 4k

   We thank the reviewer for catching this omission. We have added the full definition for the abbreviation MEF, "mouse embryonic fibroblast", to the figure legends for Figures 2 and 4 in the revised manuscript.

7. The trogocytosis could be identified with cellular co-cultures of murine cells, can this mechanism also be identified in human macrophages as co-culture, or identified in tissues as you need to have proper high intensity binding of macrophages and T cells.

   We thank the reviewer for this important suggestion to explore whether this trogocytosis-like process occurs in human macrophages or in tissue contexts. While our study primarily focuses on murine macrophages to define the cell biology of live-cell sampling and subsequent cross-presentation, we have made substantial efforts to extend these findings and report these here.

[REDACTED]

These results suggest that analogous uptake phenomena may occur in other systems, but comprehensive validation in human cells and tissues will require future studies optimized for those contexts.

8. The part of antibody opsonisation and integrin is rather vague and is confirmed only with extended data, would this mean that FcR are involved in creating and requirement to enhance this cell sampling. This part of receptors being involved like CR3 etc could be further extended and included in the figure within the paper not as extended. Furthermore this needs to be explained better how receptor mediated cell/cell contact results in the cytoplasma of one cell taken up by the macrophage.

   We agree with the reviewer that this aspect warranted clarification and additional mechanistic data. In the revised manuscript, we added a suite of loss-of-function and perturbation experiments to define contributing receptors and signaling/cytoskeletal requirements for ZsGreen sampling in our system. We have incorporated these data into the main **Figure 3** as requested as well as new **Extended Data Fig. 4-6.**

   First, using blocking antibodies and genetic KO, we show that antibody-dependent enhancement of ZsGreen uptake requires FcγR binding and signaling through the ITAM-containing adaptor FCER1G (**Reviewer Fig. 13**). These data are incorporated into **Figure 3** and **Extended Data Fig. 4** of the revised manuscript.

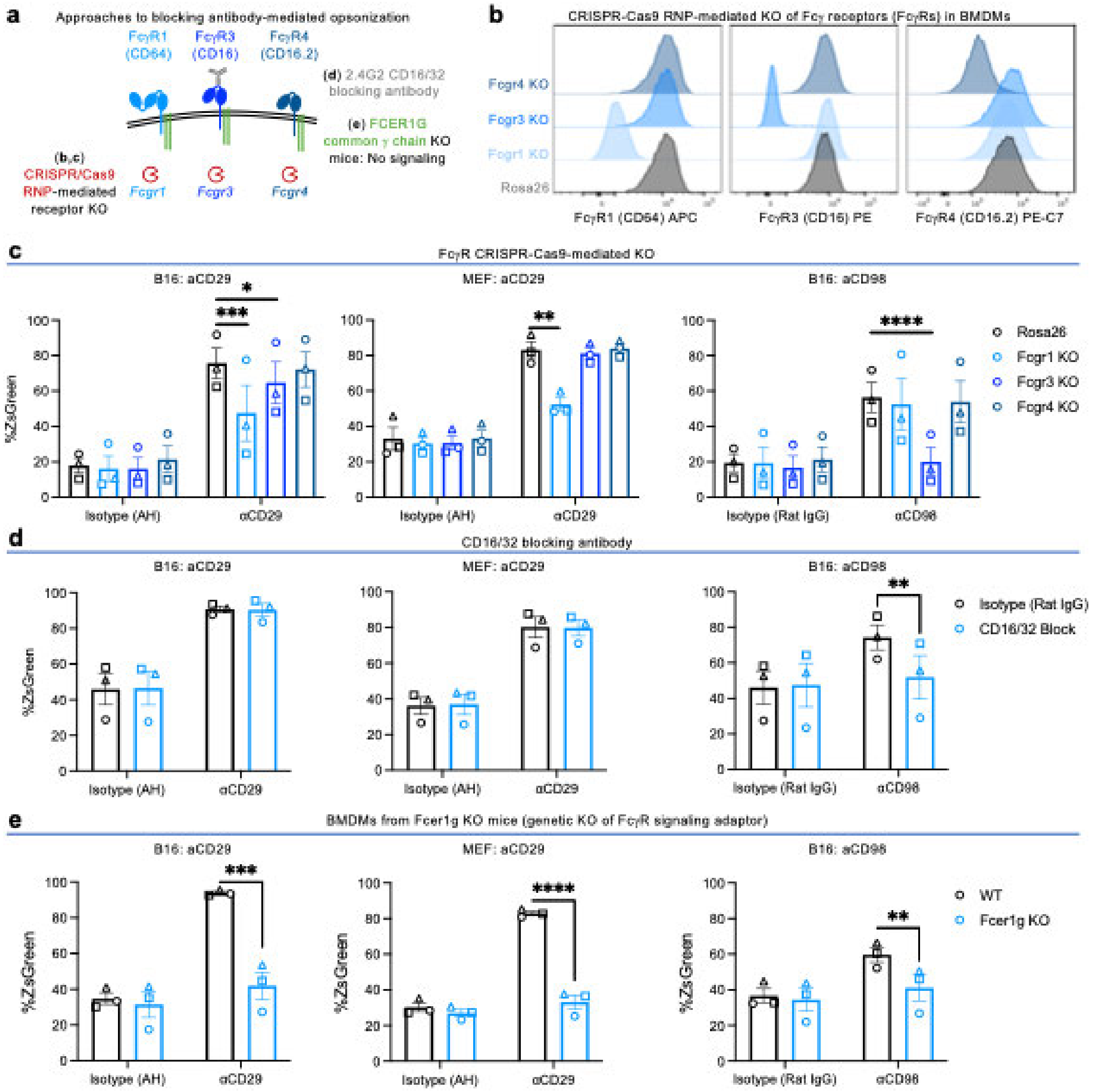

Reviewer Fig. 13: Increased uptake after antibody opsonization depends on FcγR binding and signaling

a, Schematic outlining methods used to block or knock-out (KO) FcγR binding and signaling. **b-c**, BMDMs targeted at the *Rosa26* control locus or receptor loci using CRISPR-Cas9 RNP and co-cultured with antibody-opsonized B16-ZsGreen or MEF-ZsGreen target cells for 16 hours prior to flow cytometric analysis for receptor expression (**b**, representative of 3 experiments) or ZsGreen uptake (**c**). **d,** BMDMs were pre-incubated with 10 ug/mL 2.4G2 CD16/32 blocking antibody or Rat IgG2b prior to co-culture with antibody-opsonized B16-ZsGreen or MEF-ZsGreen target cells for 16 hours prior to flow cytometry analysis for ZsGreen uptake. **e**, 16-hour antigen transfer assay with BMDMs isolated from wild-type B6 mice or Fcer1g KO mice and antibody-opsonized B16-ZsGreen or MEF-ZsGreen target cells. n = 3 biological replicates with 2-3 technical replicates. Shown are mean +/- s.e.m. Two-way ANOVA with Sidak's multiple comparison. * $p < 0.05$, ** $p < 0.01$, *** $p < 0.001$, **** $p < 0.0001$.

Next, to identify other surface proteins beyond CD11b and FcR involved in this process, we used NicheNet to predict ligand-receptor interactions between our two model target cells and BMDMs (**Reviewer Fig. 14a**). Because CD11b also has established

roles in phagocytosis, we prioritized shared receptors with known phagocytic functions (PMID 34177884), confirmed their protein, and evaluated their contribution to ZsGreen uptake. Two candidates (CD29 and CD91) were excluded due to inefficient protein KO. Of the eight candidate receptors that we examined, only KO of CD93, a C-type lectin receptor, showed a modest but significant change (decrease) in ZsGreen uptake (**Reviewer Fig. 14b-d**). These results indicate that multiple surface interactions contribute to uptake, with CD11b and CD93 playing roles. We have included these data in **Extended Data Fig. 5** in our revised manuscript.

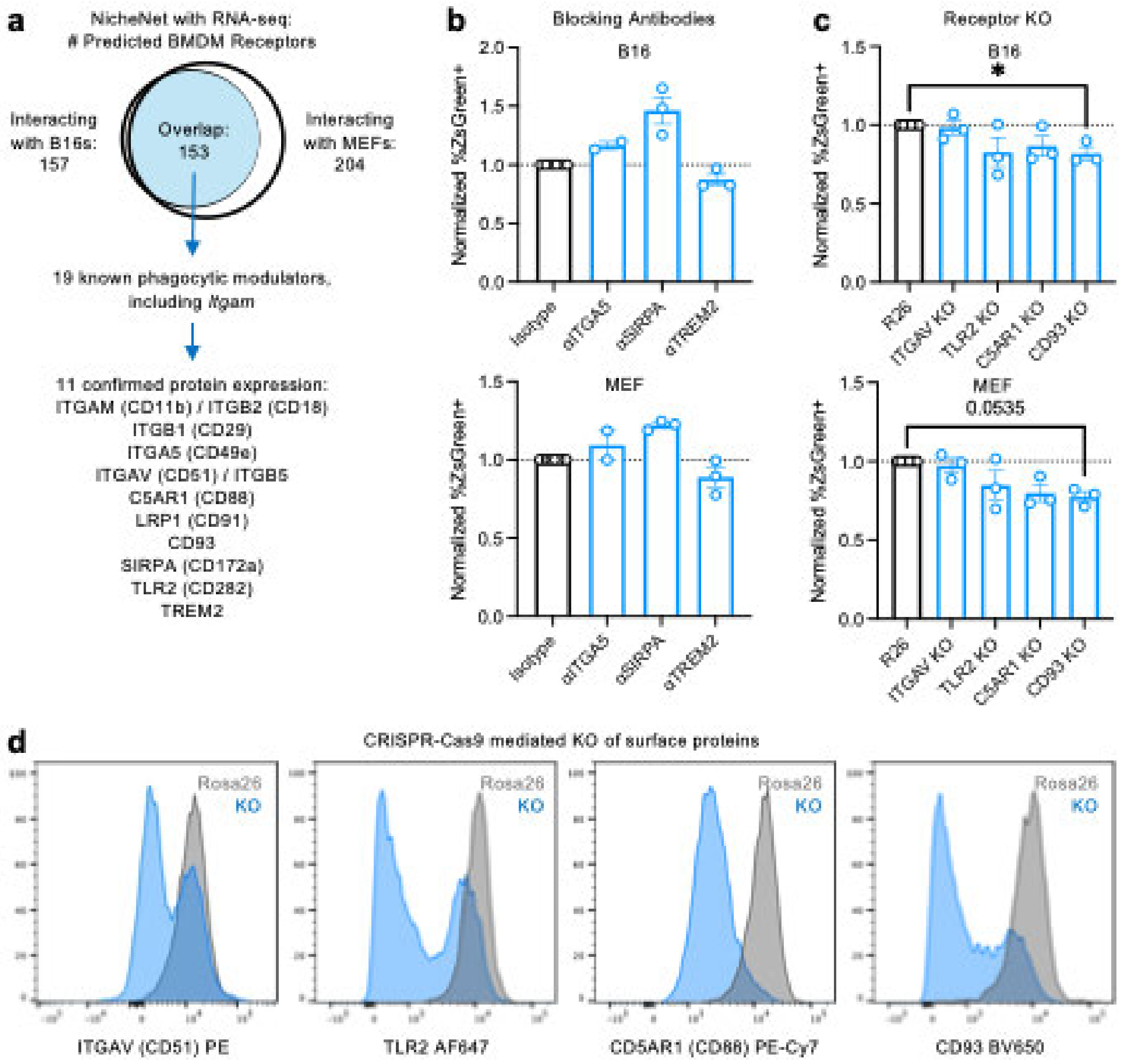

Reviewer Fig. 14: Knockout of C-type lectin receptor CD93 decreases live sampling from B16-ZsGreen target cells

a, Schematic outlining strategy to identify potential modulators of live sampling. **b**, BMDMs were co-cultured with B16-ZsGreen or MEF-ZsGreen target cells for 16 hours with 5 ug/mL blocking antibody or the appropriate isotype control prior to flow cytometric analysis of ZsGreen uptake. **c-d**, BMDMs targeted at the *Rosa26* control locus or receptor loci using CRISPR-Cas9 RNP and co-cultured with B16-ZsGreen or MEF-ZsGreen target cells for 16 hours prior to flow cytometric analysis of ZsGreen uptake (**c**) and receptor expression (**d**). Shown are mean +/- s.e.m. n = 2-3 experiments per receptor,

2-3 technical replicates per experiment. Kruskal-Wallis test with Dunn's multiple comparisons test. * p < 0.05.

We next examined signaling pathways that link receptor engagement to uptake. Using small-molecule inhibitors, we targeted signaling downstream of CD11b (Src, Syk, PI3K), small GTPases important for vesicle trafficking (Arf6, Cdc42, Rac), and actin nucleation proteins (Arp2/3, formins). Inhibiting Src signaling (PP1), PI3K signaling (GDC-0941), and Arp2/3 (CK-666) decreased but did not completely abrogate uptake (**Reviewer Fig.15**). These results suggest that receptor mediated activation of Src and PI3K, leading to Arp2/3-driven branched actin assembly, supports live-sampling. In contrast, Syk, small GTPases, and formin-driven linear actin may be dispensable, or redundant in our system. We have included these data as **Extended Data Fig. 6** for our revised manuscript.

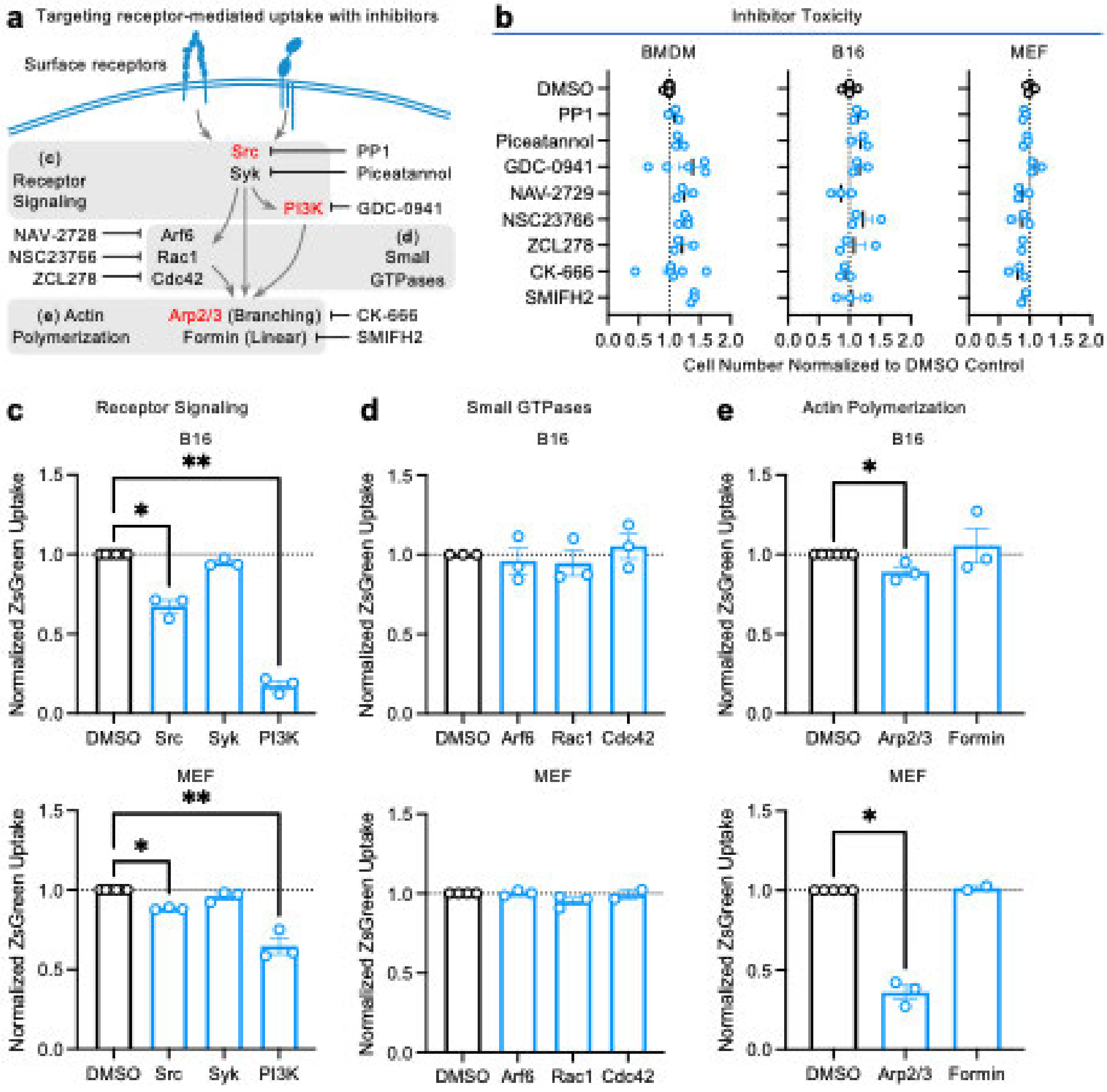

Reviewer Fig. 15: Src, PI3K, and Arp2/3 inhibition decrease live sampling
a, Schematic illustrating inhibitors used to target components of receptors-mediated uptake. **b-e,** BMDMs, B16-ZsGreen target cells, and MEF-ZsGreen target cells were cultured alone or co-culture for antigen transfer assay and treated with DMSO control, 10 uM PP1, 5 uM Piceatannol, 10 uM GDC-0941, 1.35 uM NAV-2729, 25 uM NSC23766, 10 uM ZCL278, 200 uM CK-666, or 5 uM SMIFH2 for 16 hours prior to flow cytometry analysis for cell number (**b**) or ZsGreen uptake (**c-e**). Data are normalized to DMSO control and compiled over 3-6 experiments. Shown are mean +/- s.e.m. Kruskal Wallis test and Dunn's multiple comparisons. *, $p < 0.05$; **, $p < 0.01$.

.

Referee #3 (Remarks to the Author):

Fan et al start off with the in vivo observation that tumor and tissue associated antigens are often taken up by myeloid cells in small packages - different from the whole-cell engulfment often seen in efferocytic processes. The use several transgenic systems to convincingly make this point. They then set up an in vitro assay where the investiagate the sampling of BMDMs of target cell lines and replicate the phenomenon: the cells take up fragments of live cells that have not undergone apoptosis in a process similar to trgocytosis. They then set up a flow cytometry based assay to characterize through which compartments the material taken up via endocytosis / Phago(effero)cytosis / vs. trogocytosis traffics and find that these are processes that can be distinguished by the method. Finally they measure MHCII and MHC I presentation and find that the trogocytosed material is most efficiently cross-presented.

Although the phenomenology of this study is interesting it has to be said that Figs 1-3 are not very novel and not too surprising. Trogocytosis or nibbling has been described in myeloid cells for many decades and sophisticated studies showed transcellular flux of surface material (cross dressing) and its precise physiological consequences. The process Fan et all is interesting, as it is in between trogocytosis and phagocytosis and ultimately results in cross presentation of antigen. In this sense it is novel. However, there is no functional experiment that either investigates the process on a cell biological level (what determines this type of uptake on a molecular level and can it be manipulated by gain or loss of function experiments) or on a physiological level (what is the immunological consequence of antigens being cross-presented by this pathway, what happens if it is selectively eliminated). At least one of these two levels of questioning would have to be addressed to make a high profile paper.

We appreciate the reviewer's recognition that this trogocytosis-like process resulting in cross-presentation is interesting and novel, and we agree that deeper mechanistic insight is helpful, particularly as this reviewer's proposal that this may be the same as cross-dressing proves not to be the case (reviewed below and included now as **Extended Data Fig. 9**). As requested, in the revised manuscript, we added a suite of loss-of-function and perturbation experiments to investigate the cellular mechanisms that mediate antigen uptake and trafficking for cross-presentation to T cells. Importantly, we also found a way to re-couple the non-lysosomal "alternative compartment" to the lysosome and find that this reduces cross-presentation (new **Fig. 5**). These new data amongst other additions to this manuscript demonstrate the pathway is manipulable (see your two queries) and distinct from previously described antigen sampling and handling processes.

To this reviewer's point that Figure 1-3 may be non-novel, we note especially that:

- Figure 2 is the first report to our knowledge that specifically shows that sampling can be extremely substantial (nearly a tenth the intensity of a single ZsGreen cell per macrophage) yet does not lead to cell death (and is demonstrated caspase activation negative);
- Figure 3 and 4 both address something very important: the size of these events, which as the reviewer points out is the output of an interesting process between

trogocytosis and phagocytosis, ultimately resulting in cross presentation. This biology was likely not captured in the many previous studies looking for MHCI 'cross-presentation' because, in part, they used other assays. To wit, dozens of previous papers had investigators trying (unsuccessfully in many cases) to get phagocytosed (big vesicle) material to be 'cross presented'. The list of studies is long but included the use of large beads (to 'burst' the ingested vesicle) or even addition of various drugs to divert material to Class I presentation. Beyond the biological advances, this work has built sophisticated tools to see and study these small events: Figure 3 uses imaging that can resolve these 200nm or smaller events to discover nibbling/trogocytosis occurring at a size-scale nearly an order of magnitude lower than previous mentions of 'trogocytosis' with the associated stretching and pinching mechanism (in other words, we're now in a position to see that this happens much more often, with less material ingested per bite). In Figure 4, we are able for the first time to study the distribution of ingested materials (in these v. small vesicles) into compartments, via vesicle flow at levels of resolution (tens of thousands of vesicles at a time) not previously achieved. We believe both of these greatly enrich the understanding of this biology, in addition to the subsequent figures that read out its significance.

For many in the organelle and cell-biology fields, these novelties are, we believe, other important reasons for this being a "high-profile paper".

Below, we'll highlight the new data we've added to the revision specifically answering to the requests of this particular reviewer and providing the mechanism and associated "alternative compartment" with answers to both questions posed (understanding of receptors, and consequences of loss of the alternative compartment).

Direct peptide-MHC transfer in our system

As the reviewer noted, nibbling resulting in trans-cellular membrane transfer and T cell activation has been described in myeloid cells, which we reference in our discussion. To determine if this is occurring in our system, we performed antigen transfer and T cell stimulation assays using Balb/c-derived BMDMs (**Reviewer Fig. 16a**), which should lack the H2kb allele. Staining reveal that MHC-I allele H2Kb was not detected on the B6-derived B16-ZsGreen-minOVA target cell line, Balb/c BMDMs cultured alone, or Balb/c BMDMs that had ingested ZsGreen from B16-ZsGreen-minOVA (**Reviewer Fig. 16b**). Further, Balb/c BMDMs that had taken up ZsGreen were unable to upregulate CD69 on T cells and did not drive substantial T cell proliferation (**Reviewer Fig. 16c**). Thus, while limited peptide-MHC transfer may occur in our setting (and certainly appears to in some in vivo assays done by others), the majority of CD8 T cell activation must be due to BMDM processing of ingested cytosolic antigen. We have included these data in **Extended Data Fig. 9** in our revised manuscript.

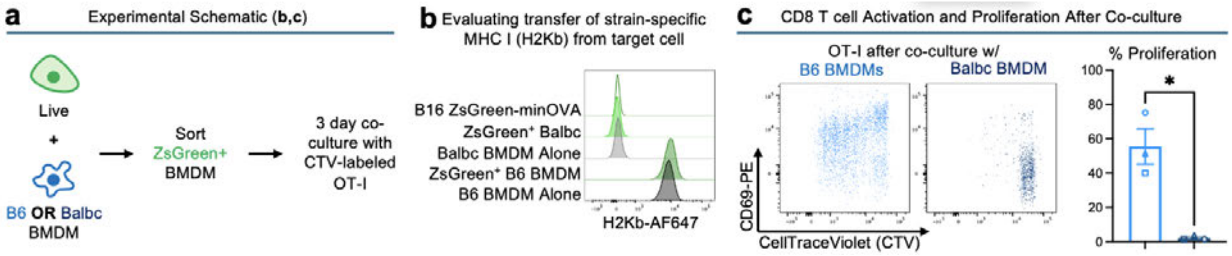

Reviewer Fig. 16: Peptide-MHC transfer does not account for the majority of T cell activation by macrophages that live-sample cytosolic antigen

a, Experimental schematic to evaluate strain specific MHC-I transfer and T cell activation by ZsGreen^{+} B6 or Balbc-derived BMDMs. **b**, H2-Kb expression on B16 ZsGreen-minOVA target cells; ZsGreen+ BMDMs after co-culture with B16 ZsGreen-minOVA cells, and BMDMs cultured alone. **c**, OT-I CD8 T cell proliferation after 3-day co-culture with FACS-isolated ZsGreen+ B6 or Balb/c BMDMs. n = 3 experiments. Shown are mean of technical replicates +/- s.e.m. Paired t-test.

Identification of receptors mediating live-sampling

As noted in our preamble, our data points to a selection of receptors and pathways that can enhance live-nibbling and we believe our revised manuscript highlights this firmly. With respect to receptors, in our original submission, we found that uptake is mediated through CD11b (CR3) through both blocking antibody experiments and genetic knockout (KO) experiments (**Extended Data Fig. 5**). To identify other surface proteins involved in this process, we used NicheNet to predict ligand-receptor interactions between our two model target cells and BMDMs (**Reviewer Fig. 17a**). Because CD11b also has established roles in phagocytosis, we prioritized shared receptors with known phagocytic functions (PMID 34177884), confirmed their protein, and evaluated their contribution to ZsGreen uptake. Two candidates (CD29 and CD91) were excluded due to inefficient protein KO. Of the eight candidate receptors that we examined, only KO of CD93, a C-type lectin receptor, showed a modest but significant change (decrease) in ZsGreen uptake (**Reviewer Fig. 17b-d**). These results indicate that multiple surface interactions contribute to uptake, with CD11b and CD93 playing partial roles. We have included these data in **Extended Data Fig. 5** in our revised manuscript.

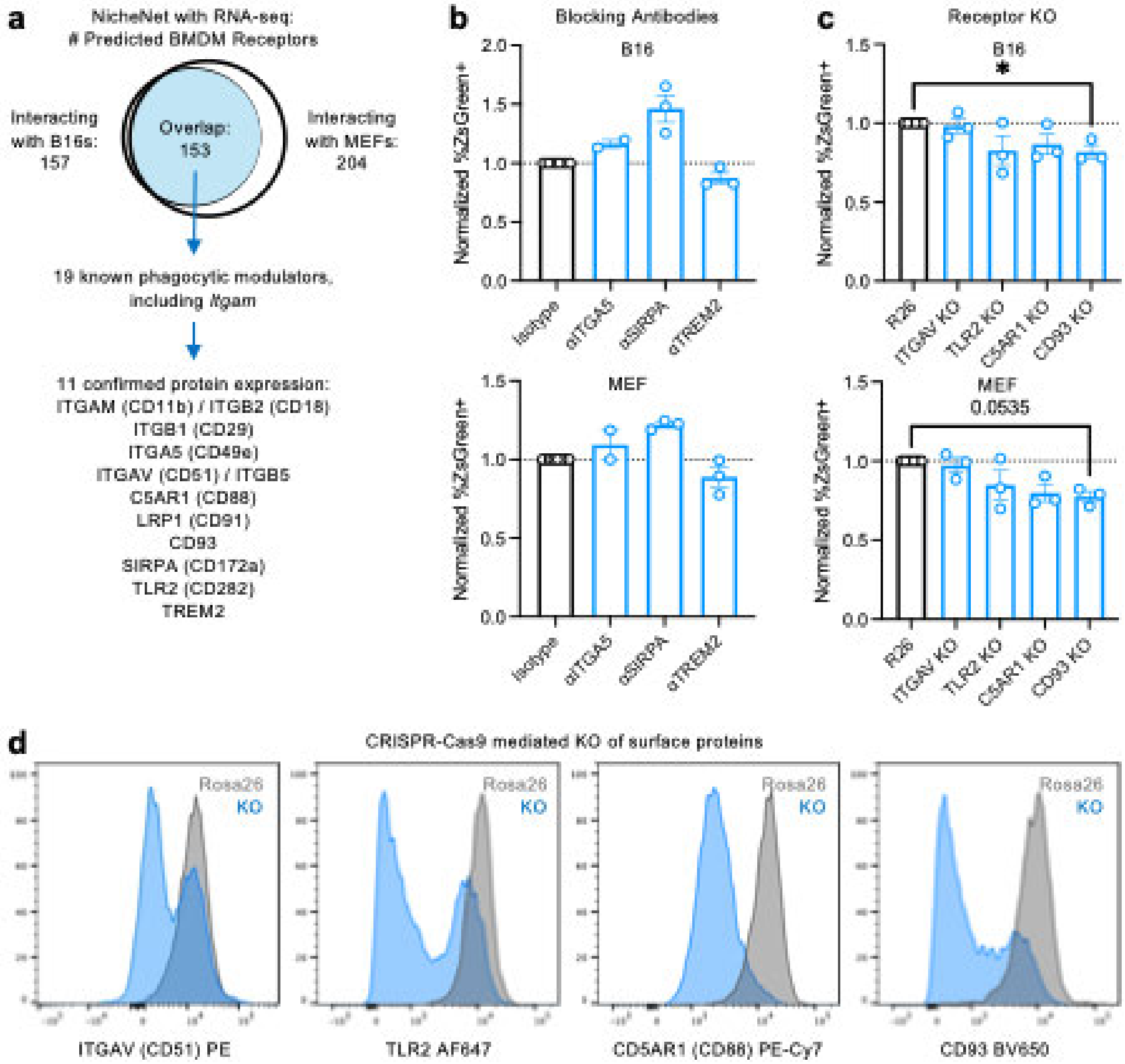

Reviewer Fig. 17: Knockout of C-type lectin receptor CD93 decreases live sampling from B16-ZsGreen target cells

a, Schematic outlining strategy to identify potential modulators of live sampling. **b**, BMDMs were co-cultured with B16-ZsGreen or MEF-ZsGreen target cells for 16 hours with 5 ug/mL blocking antibody or the appropriate isotype control prior to flow cytometric analysis of ZsGreen uptake. **c-d**, BMDMs targeted at the *Rosa26* control locus or receptor loci using CRISPR-Cas9 RNP and co-cultured with B16-ZsGreen or MEF-ZsGreen target cells for 16 hours prior to flow cytometric analysis of ZsGreen uptake (**c**) and receptor expression (**d**). Shown are mean +/- s.e.m. n = 2-3 experiments per receptor, 2-3 technical replicates per experiment. Kruskal-Wallis test with Dunn's multiple comparisons test. * $p < 0.05$.

FcγR-dependent antibody enhancement of uptake

In addition, we reported that antibody opsonization of target cells can enhances live sampling (**Extended Data Fig. 4**). Using blocking antibodies and genetic KO, we show that this enhancement is dependent on FcγR binding and signaling through the ITAM-containing adaptor FCER1G (**Reviewer Fig. 18**). These data are incorporated into **Figure 3** and **Extended Data Fig. 4** of the revised manuscript. We likewise demonstrate

decrease in sampling in muMT KO mice lacking antibodies, suggestive of serum antibodies contributing to live-sampling in vivo (**Reviewer Fig. 19**).

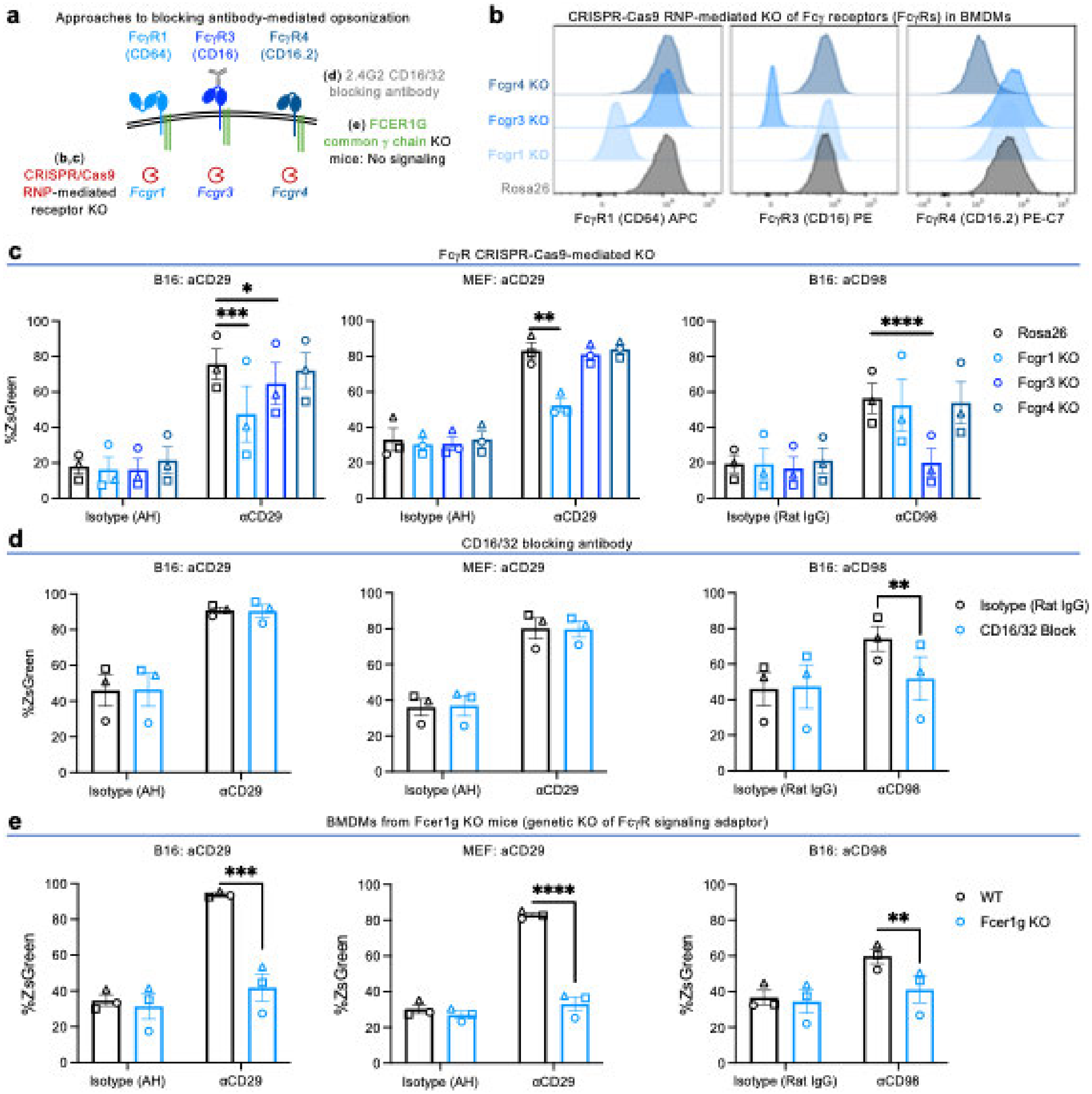

Reviewer Fig. 18: Increased uptake after antibody opsonization depends on FcγR binding and signaling

a, Schematic outlining methods used to block or knock-out (KO) FcγR binding and signaling. **b-c**, BMDMs targeted at the *Rosa26* control locus or receptor loci using CRISPR-Cas9 RNP and co-cultured with antibody-opsonized B16-ZsGreen or MEF-ZsGreen target cells for 16 hours prior to flow cytometric analysis for receptor expression (**b**, representative of 3 experiments) or ZsGreen uptake (**c**). **d**, BMDMs were pre-incubated with 10 ug/mL 2.4G2 CD16/32 blocking antibody or Rat IgG2b prior to co-culture with antibody-opsonized B16-ZsGreen or MEF-ZsGreen target cells for 16 hours prior to flow cytometry analysis for ZsGreen uptake. **e**, 16-hour antigen transfer assay with BMDMs isolated from wild-type B6 mice or Fcer1g KO mice and antibody-opsonized B16-ZsGreen or MEF-ZsGreen target cells. n = 3 biological replicates

with 2-3 technical replicates. Shown are mean +/- s.e.m. Two-way ANOVA with Sidak's multiple comparison. * p < 0.05, ** p < 0.01, *** p < 0.001, **** p < 0.0001.

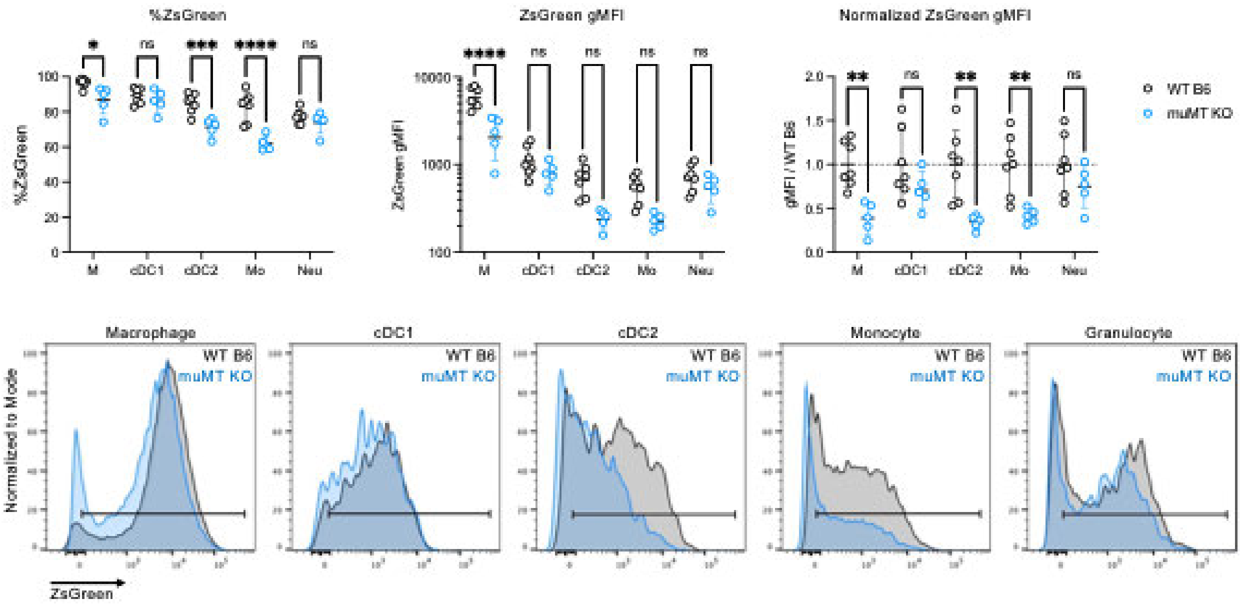

Reviewer Fig. 19: muMT KO mice lacking mature B cells display decreased ZsGreen labeling of tumor associated myeloid cells in B16-ZsGreen tumors in mice
B16-ZsGreen tumor cells were subcutaneously transplanted into wild-type B6 (WT B6, black) or muMT knockout (muMT KO, blue) and tumor-associated myeloid cells were isolated to evaluate ZsGreen labeling. cDC1, conventional dendritic cell 1; cDC2, conventional dendritic cell 2. Shown are means +/- s.d. n = 5-7 mice per condition, representative of 2 experiments. Two-way ANOVA with Sidak's multiple comparison. ns not significant, * p < 0.05, *** p < 0.001, **** p < 0.0001.

Signaling and cytoskeletal contributors to uptake

We determined the contribution of candidate signaling pathways using small-molecule inhibitors. We targeted signaling downstream of CD11b (Src, Syk, PI3K), small GTPases important for vesicle trafficking (Arf6, Cdc42, Rac), and actin nucleation proteins (Arp2/3, formins). Inhibiting Src signaling (PP1), PI3K signaling (GDC-0941), and Arp2/3 (CK-666) decreased but did not completely abrogate uptake (**Reviewer Fig. 20**). These results suggest that receptor mediated activation of Src and PI3K, leading to Arp2/3-driven branched actin assembly, supports vesicle formation. In contrast, Syk, small GTPases, and formin-driven linear actin may be dispensable, redundant, or incompletely inhibited in our system. We have included these data as **Extended Data Fig. 6** for our revised manuscript.

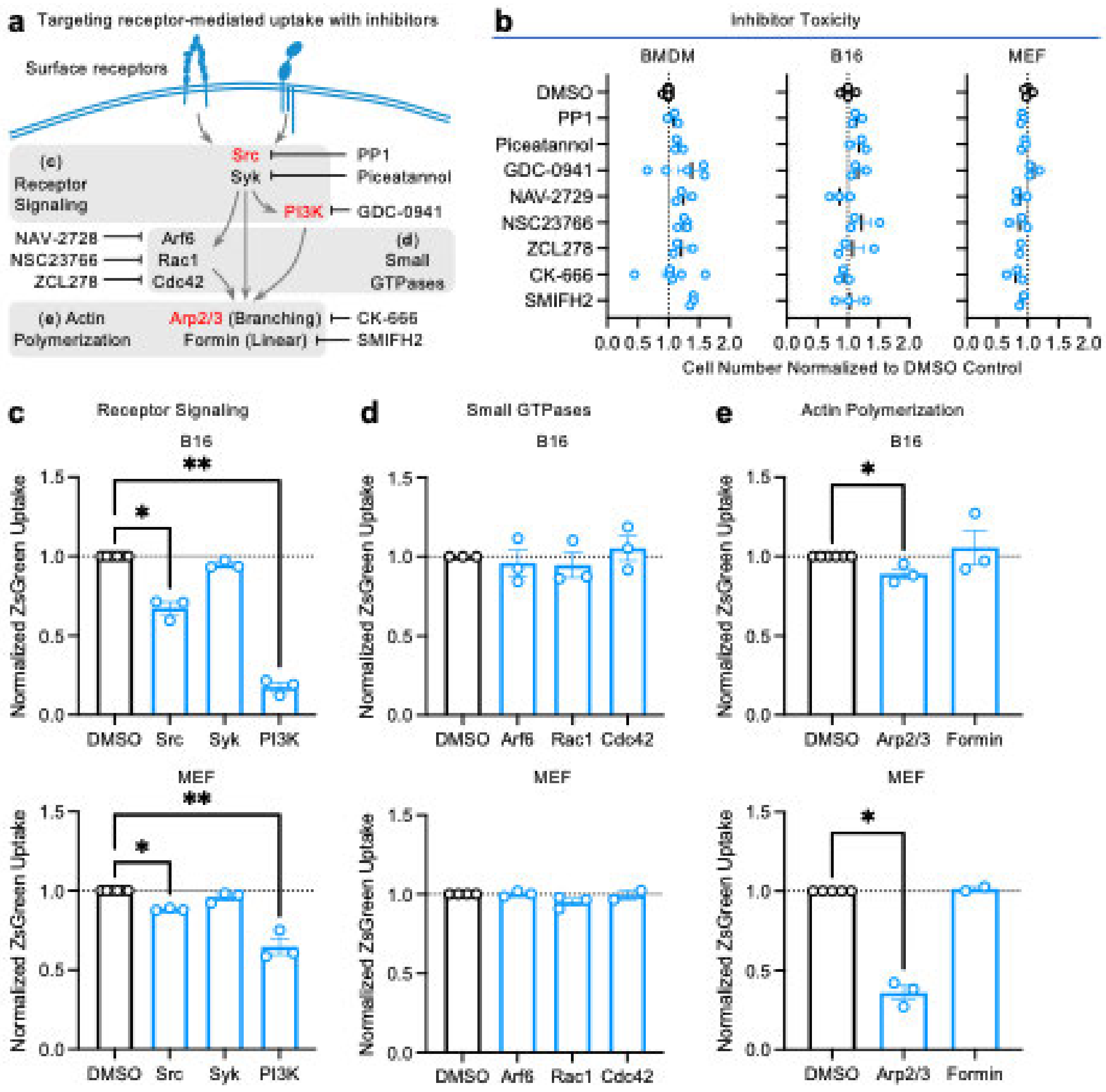

Reviewer Fig. 20: Src, PI3K, and Arp2/3 inhibition decrease live sampling

a, Schematic illustrating inhibitors used to target components of receptor-mediated uptake. BMDMs, B16-ZsGreen target cells, and MEF-ZsGreen target cells were cultured alone or co-culture for antigen transfer assay and treated with DMSO control, 10 uM PP1, 5 uM Piceatannol, 10 uM GDC-0941, 1.35 uM NAV-2729, 25 uM NSC23766, 10 uM ZCL278, 200 uM CK-666, or 5 uM SMIFH2. **b-e,** Cell numbers (**b**) and ZsGreen uptake (**c-e**) were analyzed by flow cytometry'. Data are normalized to DMSO control and compiled over 3-6 experiments. Shown are mean +/- s.e.m. Kruskal Wallis test and Dunn's multiple comparisons. *, $p < 0.05$; **, $p < 0.01$.

Diverting antigen trafficking from the alternative compartment to the lysosome

Finally, we found a way to redirect antigen trafficking from the alternative compartment to LAMP1^{+} compartments and demonstrate a subsequent loss of T cell activation. Specifically, after a panel of candidate approaches, we found that compared to Rosa26-targeted controls, genetic KO of the sorting nexin 27 (SNX27) in BMDMs redirected ingested ZsGreen from the unmarked alternative compartment to LAMP1+ lysosomes

and decreased CD8 T cell activation (**Reviewer Fig. 21**). This links the formation of the alternative compartment with T cell priming capacity in macrophages that live-sample. We present these data as **Figure 5** and **Extended Data Fig. 10** in our revised manuscript.

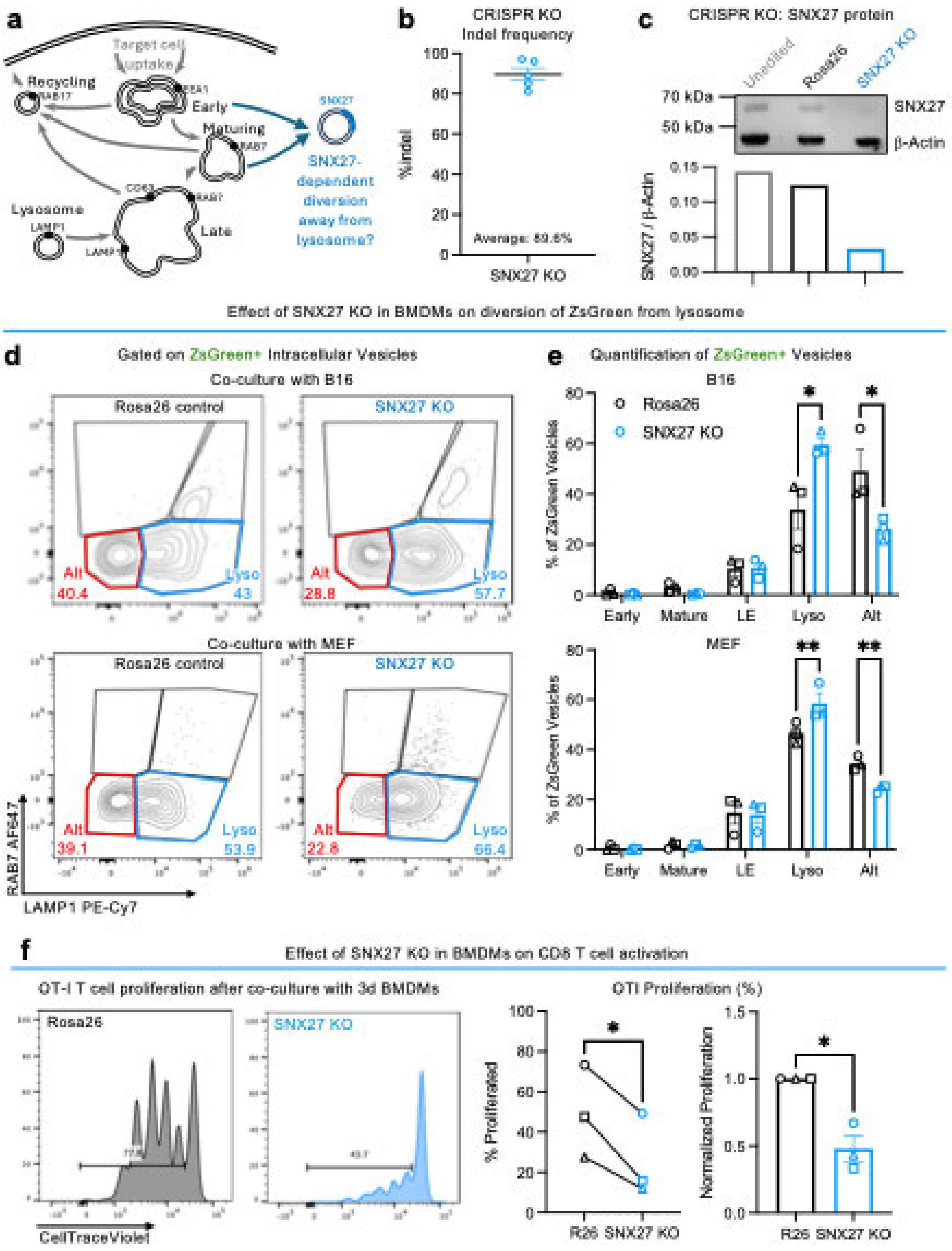

Reviewer Fig. 21: SNX27 KO in macrophages increases antigen trafficking to lysosomes and decreases CD8 T cell activation
a, Schematic illustrating SNX27-dependent diversion of material away from lysosomal maturation into alternative endocytic pathways. **b-c**, Validation of SNX27 KO in BMDMs by (**b**) indel quantification by ICE analysis (mean +/- s.d.) and (**c)** western blot (representative of 2 experiments). **d-e**, CRISPR-Cas9-targeted BMDMs were co-cultured with ZsGreen target cells to evaluate ZsGreen trafficking. Shown are mean +/- s.e.m. n = 3 biological replicates. **f**, OT-I CD8 T cell proliferation after 3-day co-culture with FACS-isolated ZsGreen+ Rosa26-targeted and SNX27 KO BMDMs that live-sampled B16-ZsGreen-minOVA target cells. Shown are mean +/- s.e.m. n = 3 biological replicates. Two-tailed paired t-test or two-way ANOVA with Sidak's multiple comparison. *, $p < 0.05$, ** $p < 0.01$.

We appreciate the reviewers' guidance. We believe these additions substantially drive a change in our understanding of antigen-sampling and cross-presentation by identifying manipulable determinants of entry, signaling, and trafficking in live-cell sampling and linking this pathway with cross-priming of CD8 T cells.

We thank all three reviewers for contribution to this work and their recognition of the substantial improvement in our manuscript. All specific comments are addressed below.

Referee #1 (Remarks to the Author):

The authors have addressed my comments and the revised manuscript is improved, Sebastian Amigorena

We thank the reviewer for his helpful comments in improving our manuscript.

Referee #2 (Remarks to the Author):

The authors addressed all remarks and improved the paper considerably. For me the only question that remains is that it seems that the receptors involved in the binding-uptake of life cells via trogocytosis such as CD93 are the same as phagocytosis for dead cells, where these receptors have been described intensively. If these receptors are the same for the initial interaction but consequently instruct differential routing remains difficult.
Also if this proces will happen simultanteously would the phagocytosis of dead cells hamper the trogo/like uptake of live cells that induces CD8 T cell proliferation?

We thank the reviewer for these thoughtful questions. As noted, the mechanisms governing phagocytosis and trogocytosis are largely overlapping. Indeed, several receptors previously implicated in trogocytosis by macrophage-like cells are also well-characterized phagocytic receptors, namely CD11b, LRP, and FcγR. We therefore agree that receptor identity alone is unlikely to fully explain differential routing.

We speculate that the extent, combination, or spatial organization of receptor engagement, together with non-receptor-associated factors, may influence routing decisions. For example, recent reports published after our initial submission suggest that mechanical forces can promote macrophage trogocytosis versus phagocytosis decisions (PMIDs 41387582, 41042177), raising the possibility that biophysical features of uptake may also shape antigen trafficking.

Additionally, we agree that the consequences of simultaneous uptake of dead- and live-associated material remain an important open question. Whether such material is maintained as separate antigen pools or intermixed, or whether these mechanisms exert trans effects on macrophage priming capability is not yet known. We view these questions as important directions for future investigation.

Referee #3 (Remarks to the Author):

The authors added a substantial number of experiments that clarified many of the specific questions asked by Rev 1 and Rev 2. The most substantial addition was the SNX27Ko, that shifted the lysosomal trafficking pathway. However, my problem with the manuscript is still that it does neither describe a fundamentally new phenomenon, nor clarify a cell biological mechanism on a molecular level, nor assign a clear immunological function to the sampling mechanism. The authors acknowledge these unknowns in their discussion. I find it especially unsatisfying that the authors now have a molecular target (SNX27) that they could employ to test if the sampling mechanism leads to tolerance or immunity, e.g. in a tumor setting.

We appreciate the reviewer's thoughtful assessment. We agree that understanding the precise molecular mechanisms and whether this sampling mechanism ultimately promotes tolerance or immunity are important and exciting questions.

The goal of this work is to characterize a distinct and previously underappreciated mode of material acquisition that we now understand to lie between classical trogocytosis and phagocytosis and that can result in efficient cross-presentation. As the reviewer previously noted, this process is novel in that regard. In our revision experiments, we have used perturbation experiments to uncover components of the mechanism governing the uptake and trafficking of the sampled material, including via knockout of SNX27.

We believe the principal advances of this study are the identification and quantitative characterization of this sampling mechanism, the demonstration that it is distinct from previously described antigen acquisition pathways, and the development of imaging and vesicle flow approaches that enable resolution of these small, previously inaccessible events. While the identification of SNX27 provides a molecular handle on this pathway, determining how modulation of this process biases tolerance versus immunity in vivo would require substantial additional work beyond the scope of the current manuscript.

We have therefore aimed to clearly delineate what is established here and what remains to be addressed in future studies, and we believe that defining this process and its manipulability represents an important conceptual and technical advance that lays the ground for subsequent functional investigations.
